# Clinical and Economic Consequences of a First Major Bleeding Event in Patients Treated with Direct Factor Xa Inhibitors in Spain: A Long-Term Observational Study

**DOI:** 10.3390/jcm13144253

**Published:** 2024-07-21

**Authors:** Carlos Escobar, Beatriz Palacios, Miriam Villarreal, Martín Gutiérrez, Margarita Capel, Unai Aranda, Ignacio Hernández, María García, Laura Lledó, Juan F. Arenillas

**Affiliations:** 1 Cardiology Department, University Hospital La Paz, 28046 Madrid, Spain; 2 BioPharmaceuticals Medical, AstraZeneca, 28050 Madrid, Spain; beatriz.palacios@astrazeneca.com (B.P.); miriam.villarreal@astrazeneca.com (M.V.); martin.gutierrez@astrazeneca.com (M.G.); 3 BioPharmaceuticals Corporate Affairs & Market Access, AstraZeneca, 28050 Madrid, Spain; margarita.capel@astrazeneca.com; 4 Global Medical Affairs, BioPharmaceuticals Medical, AstraZeneca, Gaithersburg, MD 20878, USA; unai.aranda1@astrazeneca.com; 5 Atrys Health, 28002 Madrid, Spain; ihernandez@atryshealth.com (I.H.); mgmarquez@atryshealth.com (M.G.); llledo@atryshealth.com (L.L.); 6 Neurology Department, Comprehensive Stroke Center, Hospital Clínico Universitario, 47003 Valladolid, Spain; juanfarenillas@gmail.com; 7 Clinical Neurosciences Research Group, Department of Medicine, University of Valladolid, 47003 Valladolid, Spain

**Keywords:** atrial fibrillation, cost, direct oral anticoagulants, healthcare resource utilization, major bleeding, reversal agents, venous thromboembolism

## Abstract

**Aims:** Our aims were to describe the clinical characteristics, adverse clinical events, healthcare resource utilization (HCRU) and costs of patients with major bleeding during direct Factor Xa inhibitor (FXai) use. **Methods:** This is a retrospective cohort study that included secondary data from computerized health records of seven Spanish Autonomous Communities. Patients with a first major bleeding during treatment with a direct FXai were analyzed during a 3-year period. **Results:** Of 8972 patients taking a direct FXai, 470 (5.24%) had major bleeding (mean age (SD) 77.93 (9.71) years, 61.06% women). The most frequent indications for using FXais were atrial fibrillation (78.09%) and venous thromboembolism (17.66%). Among those with major bleeding, 88.94% presented with gastrointestinal bleeding, 6.81% intracranial bleeding, 2.13% trauma-related bleeding and 4.26% other major bleeding. Prothrombin complex concentrates were used in 63.19%, followed by transfusion of blood products (20.21%) and Factor VIIa (7.66%). In total, 4.26% of patients died in the hospital due to the first major bleeding. At the study end (after 3-year follow-up), 28.94% of the patients had died, 12.34% had a myocardial infarction and 9.15% an ischemic stroke. At year 3, overall bleeding cost was EUR 5,816,930.5, of which 79.74% accounted for in-hospital costs to treat the bleeding episode. **Conclusions:** Despite the use of replacement agents being high, major events were common, with a 29% mortality at the end of the follow up, and HCRU and costs were high, evidencing the need for new reversal treatment strategies.

## 1. Introduction

Anticoagulation is the key treatment for the management of patients with atrial fibrillation (AF), acute venous thromboembolism (VTE) and some patients soon after bioprosthetic valve implantation to prevent the development of arterial and/or venous thromboembolic complications [1,2,3,4].

The use of direct oral anticoagulants (DOACs) in clinical practice has revolutionized the treatment of these patients, compared to the traditional approach (vitamin K antagonists in patients with AF and low-molecular-weight heparins, followed by VKAs in patients with VTE), providing a better benefit–risk profile [5,6,7]. Unfortunately, although DOACs are safer than VKAs, the incidence of bleeding remains an important problem in real-life settings. In fact, major bleeds are the most disturbing complication associated with anticoagulation, leading to an increased morbidity and mortality, as well as the increased healthcare resource utilization (HCRU) and associated costs [8,9]. 

The management of patients with major bleeding is complex and challenging [10,11], and guidelines have provided practical algorithms on the management of patients with major bleeding treated with oral anticoagulants, including direct Factor Xa inhibitors (FXais). Of note, while idarucizumab is available for dabigatran, there is not yet a specific reversal agent for direct FXais (i.e., andexanet alfa) available in Spain in clinical practice at this moment. As a result, it is necessary to ascertain how these patients are being treated in clinical practice, as well as the clinical consequences, HCRU and associated costs after a major bleed has occurred. Although other studies have analyzed outcomes after a major bleeding event, these data are lacking in Spain. As there may be relevant differences across countries due to the way major bleeds are managed, it is important to generate the Spanish epidemiological data for the local medical community [1,12,13,14]. 

In a recent study, we presented the results of a cohort study, showing that in Spain, among patients taking a direct FXai, the incidence rates of major total, gastrointestinal, intracranial and fatal bleeding events were 10.1, 9.0, 0.7 and 0.4 per 100 person-years, respectively [15], but further information when major bleeding occurs is warranted. The objective of this study was to analyze the clinical characteristics of subjects with a major bleed during direct FXai use, and to estimate the incidence of and the risk factors for the development of adverse clinical events (total mortality, cardiovascular death, acute myocardial infarction, ischemic stroke, acute kidney and liver failure), as well as HCRU and associated costs following major bleeding in patients with a first major bleeding event in temporal association with direct FXai treatment.

## 2. Methods

This was an observational cohort study, with a retrospective design that analyzed secondary data from electronic health records in seven Spanish Autonomous Communities from the BIG-PAC^®^ database (initiated in 2012). Different studies have demonstrated the representativeness of this database for the Spanish population [16,17,18]. 

In the previous study, adults with a prescription of an oral direct FXai (i.e., apixaban, edoxaban or rivaroxaban) at a therapeutic dose, for the management of VTE, AF or non-mechanical cardiac–valve replacement, between 1 January 2013 and 31 December 2022 were analyzed [15]. Additionally, to be included, patients had to have at least 12 months of data available in the database before the prescription of anticoagulant. The present study included those patients who had a first-ever major bleeding event during their treatment with a direct FXai.

Major bleeding was defined as all critical site bleeding (defined with the International Classification of Diseases (ICD)-10 codes based on the International Society on Thrombosis and Haemostasis definitions and anemia ICD-10 codes) and for other locations, if the bleeding was fatal or led to hospitalization. Trauma-related ICD-10 bleeding codes were a subset of the intracranial or other bleeding codes. Other major bleeding included genitourinary, respiratory and other bleeding not included elsewhere [15]. The exclusion criteria included therapeutic non-FXai anticoagulation use or prophylactic anticoagulation use in the 60 days before the major bleeding event, the history of a major bleeding event before the start of treatment with a direct FXai, palliative care initiation any time before the major bleeding event of interest and pregnancy any time between the direct FXai treatment episode start and the major bleeding event of interest. 

The index date (start of observation in these patients) was defined as the day of the first major bleed and the end of the observation period occurred when the first of any of the following events happened: 3 years after the index bleeding event, recording of pregnancy, completion of the study period, disenrollment from the database, end of data collection, death or start of palliative care. Of note, for analyses on HCRU and bleeding events, follow-up did not end when a clinical outcome of interest had occurred. No specific diagnostic or therapeutic procedures were performed for inclusion in the study, due to the retrospective observational nature of the study. The study was authorized by the Research Ethics Committee of Consorci Sanitari de Terrassa, Barcelona, Spain. The need for obtaining written informed consent was waived by the Research Ethics Committee, because the study collected secondary anonymized data.

At baseline (the day prior to index day), data from biodemographics, cardiovascular risk factors, vascular disease and other comorbidities were recorded. Clinical conditions were defined by means of the medical codes entered by practices, with the ICD, Ninth Revision (ICD-9), and Tenth Revision (ICD-10) (all converted to ICD-10 in the database). Laboratory tests closest to the index date were collected. Concomitant treatments (within 120 days prior to enrolment, 180 days in the case of anticancer drugs) were also captured. Treatments were derived from issued prescriptions coded utilizing anatomical therapeutic chemical codes (ATC medication code: “A10”). Data were analyzed in the whole study population, and according to the type of major bleeding, the use of prothrombin complex concentrates, the type of FXai and the FXai indication.

The analyzed outcomes were the development of a first and subsequent clinical event (death from any cause, cardiovascular death, acute myocardial infarction, ischemic stroke, acute kidney failure or acute liver failure). Incidence and event rates of clinical events were calculated. Incidence rates were defined as the total number of incident events of interest divided by the total person time at risk and event rates, as the total number of events, including recurrent events divided by the total person time of follow-up, during the entire follow-up. Incidence and event rates were estimated in the whole study population, and according to the type of major bleeding, the use of prothrombin complex concentrates, the type of FXai and the FXai indication. Cumulative incidence curves for events (acute myocardial infarction, stroke, cardiovascular death and overall death) were calculated. In addition, risk factors for the development of these clinical events were calculated.

HCRU included the number and associated healthcare costs of all healthcare resources, including the number and length of hospital admissions, inpatient procedures and investigations (laboratory and radiology), outpatient [general practitioner (GP) and specialist] visits, prescriptions, general practice visits and referrals. Information on cost data was inferred from the eSalud database [19] and the pharmaceutical prescription costs were based on the full price of products [20]. Costs of absence from work were estimated by multiplying the number of days of absence from work due to illness by the mean daily salary of a worker in Spain [21] (Supplementary Table S1). All-cause and related to bleeding total and per patient costs were calculated at 6 months, 1 year, 2 years and 3 years after the index episode of major bleeding. HCRU and costs were estimated in the whole study population, and according to the type of major bleeding, the type of FXai and the FXai indication. 

## 3. Statistical Analysis

Demographic and clinical data were reported with descriptive statistics. Qualitative data were shown by their absolute (n) and relative (%) frequencies. Quantitative data were reported with the mean and standard deviation (SD). To compare continuous variables between prothrombin complex concentrates users vs. non-users, a two-sample *t*-test was used for variables normally distributed, and the Mann–Whitney U test was used for those non-normally distributed. The chi-square test was used for categorical variables. Wald contrast was used for the incident rates of clinical outcomes. Incidence and event rates for clinical events were described per 100 person-years with their 95% confidence intervals. Cumulative incidence curves for events (acute myocardial infarction, stroke, cardiovascular death and overall death) were calculated. The area under the curve (AUC) of these curves was calculated using the trapezoidal rule. The strength of association between potential risk factors (all baseline clinical characteristics) and the risk of clinical events (for acute myocardial infarction, stroke, cardiovascular death and all-cause death) was calculated using Cox regression models calculating the hazard ratio with the 95% confidence intervals. To investigate HCRU following major bleeding, cumulative HCRU rates were calculated, and respective costs were estimated by multiplying event numbers with costs per event. A *p*-value less than 0.05 was considered to be statistically significant. All statistical techniques were calculated using Stata MP Version 14.2 (StataCorp LLC., College Station, TX, USA). 

## 4. Results

**Figure 1:**
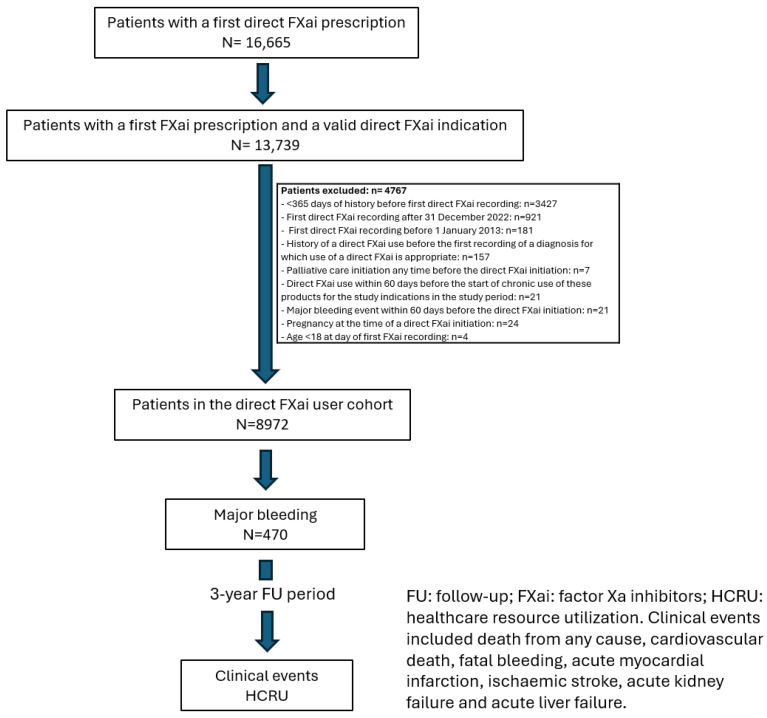
Flow chart of the study.

Out of 1.9 million patients included in the BIG-PAC^®^ database, 16,665 patients had received their first direct FXai prescription, of whom 13,739 had a study indication. After exclusion of 4767 patients for different causes, 8972 patients were finally analyzed. Of these, 470 (5.24%) had had a major bleed and were followed-up during a 3-year period (Figure 1). In this study, the data of patients with a major bleed are presented.

The clinical characteristics at baseline of the whole study population are reported in Table 1. The mean age (SD) was 77.93 (9.71) years old, 61.06% were females, 78.72% had arterial hypertension, 33.83% type 2 diabetes, 23.83% heart failure, 17.23% chronic kidney disease, 15.32% ischemic heart disease and 8.09% cerebrovascular disease. The most frequent indications for the use of FXai were AF (78.09%), VTE (17.66%) and non-mechanical cardiac–valve replacement (4.26%). When a major bleeding occurred, prothrombin complex concentrates were used in 63.19% of patients, followed by transfusion of blood products (20.21%) and Factor VIIa (7.66%). Surgical procedures used to correct bleeding were performed in 18.72% of the cases of major bleeding. Twenty (4.26%) patients died in hospital due to the first major bleeding event. 

Overall, 418 (88.94%) patients presented with gastrointestinal bleeding, 32 (6.81%) intracranial bleeding, 20 (4.26%) other major bleeding and 10 (2.13%) trauma-related bleeding (included intracranial and other major bleeding). Clinical characteristics at baseline were also analyzed according to the type of major bleeding, showing that trauma-related bleeding was more common in the elderly, and prothrombin complex concentrates were more frequently used in cases of intracranial bleeding, whereas transfusion of blood products was common in cases of gastrointestinal bleeding. 

In-hospital mortality during the first bleeding episode was higher in cases of trauma-related bleeding (70.0%), followed by intracranial hemorrhage (28.13%), other bleeding (10.00%) and gastrointestinal bleeding (2.15%) (Table 2). Baseline characteristics were also analyzed according to the use of prothrombin complex concentrates, the type of FXai and the FXai indication (Table 1 and Supplementary Table S2). No significant differences were found in the baseline clinical profile according to the use of prothrombin complex concentrates.

Mean CHA_2_DS_2_-VASc and HAS-BLED scores in the overall group and by FXai bleeding type are shown in Supplementary Table S3. 

**Figure 2:**
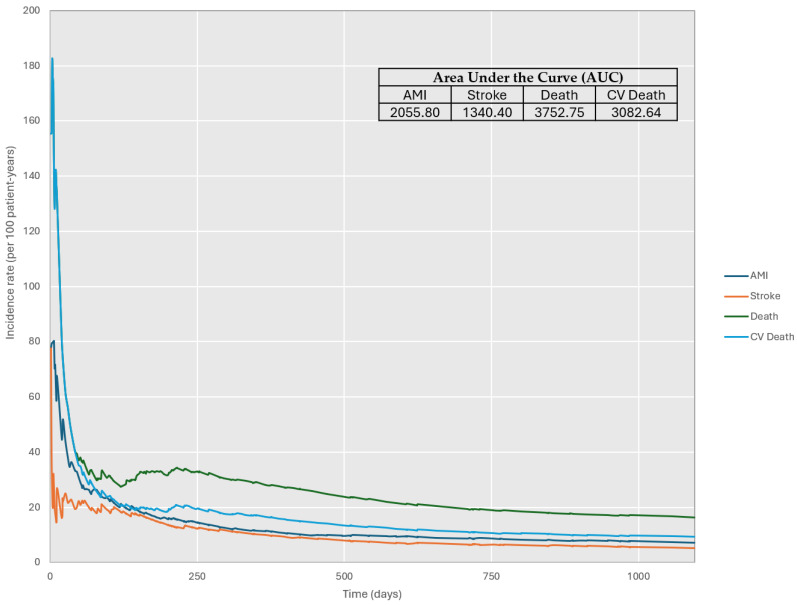
Cumulative incidence (per 100 patient-years) curves for events and corresponding AUCs. AMI: acute myocardial infarction; CV: cardiovascular.

Cumulative clinical outcomes during the study period are presented in Table 2. The incidence rates per 100 person-years during the index hospitalization were death from any cause 18.17 (14.68–21.66), cardiovascular death 18.17 (14.68–21.66), acute myocardial infarction 4.64 (2.74–6.54), ischemic stroke 0.93 (0.06–1.8) and acute kidney failure 0.92 (0.06–1.78) (corresponding to 4.26, 4.26, 1.06, 0.21 and 0.21%, respectively). These numbers were 17.2 (12.91–21.49), 17.20 (12.91–21.49), 2.94 (1.02–4.86), 1.47 (0.10–2.84) and 0, respectively, among those patients treated with prothrombin complex concentrates during hospitalization. After 3 years of follow-up, incidence rates per 100 person-years were death from any cause 16.42 (13.07–19.77), cardiovascular death 9.3 (6.67–11.93), acute myocardial infarction 7.34 (4.98–9.7), ischemic stroke 5.42 (3.37–7.47), acute kidney failure 4.17 (2.36–5.98) and acute liver failure 0.97 (0.08–1.86). The incidence rates of clinical events decreased over time. There were few recurrent events, with acute myocardial infarction being the most common (with five additional cases reported). Cumulative incidence curves for events (acute myocardial infarction, stroke, cardiovascular death and overall death) were calculated (Figure 2). Cumulative clinical outcomes were evaluated according to the type of major bleeding, the use of prothrombin complex concentrates, the type of FXai and the FXai indication (Table 2 and Supplementary Table S4). Except for a higher incidence rate of acute myocardial infarction during the index hospitalization, no significant differences were found in cumulative clinical outcomes according to the use of prothrombin complex concentrates. Mortality incidence rates were higher in those patients with trauma-related bleeding, followed by intracranial hemorrhage, other major bleeding and gastrointestinal bleeding. In addition, mortality rates were higher in those patients who were not treated with prothrombin complex concentrates.

The cumulative HCRU for 3 years from the index date was analyzed, including outpatient (GP and specialist) visits and hospitalizations, length of hospital stays, laboratory/radiology investigations, number of prescriptions and work absences (Table 3 and Table 4). At the study end, all-cause HCRU rates of outpatient visits, laboratory/radiology investigations and hospitalization were 1437.8 (1120.59–1755.01), 346.02 (303.01–389.03) and 83.31 (79.94–86.68) per 100 person-years, respectively, with a decrease over time from the 6-month time point following the index date. These numbers were 898.73 (871.46–926.00), 216.35 (179.12–253.58) and 63.99 (59.65–68.33) per 100 person-years, respectively, for bleeding-related HCRU, following the same pattern of decrease (with 53% of all bleeding-related outpatient visits and 92% of hospitalizations occurring in the initial 6 months after the first major bleed). When considering all patients in the cohort, bleeding-related length of hospital stay and mean number of days (SD) due to work absences per patient were 12.20 (4.81) and 2.49 (8.65) days, respectively. The HCRU was also analyzed according to the type of major bleeding, the type of FXai and the FXai indication (Supplementary Tables S5 and S6), with hospitalization rates being higher in those patients who presented with trauma-related bleeding, followed by intracranial hemorrhage and, to a lesser extent, other bleeding and gastrointestinal bleeding.

Cumulative costs for 3 years from the index date are shown in Table 5. The total overall cost reached EUR 7,843,056.4 at year 3 (EUR 5,780,203.3 [73.70%] in the first 6 months), of which 76.99% accounted for hospitalizations, 18.02% for outpatient care and 4.99% for indirect costs (cost of absence from work). Overall prescriptions accounted for 8.72% of the total cost. In the case of bleeding-related costs, overall bleeding cost was EUR 5,816,930.5 (EUR 4,930,469.4 [84.76%] in the first 6 months) of which 79.74% accounted for hospitalizations, 14.75% for outpatient care and 5.52% for indirect costs. Overall prescriptions accounted for 7.86% of the total cost. When considering all patients, mean (SD) cost per patient was EUR 16,687.35 (7661.35) and EUR 12,376.45 (4259.28) for overall and bleeding-related costs (EUR 9868.51 for inpatient bleeding costs), respectively. Cumulative costs for 3 years from the index date were also calculated according to the type of the first major bleed, the type of FXai and the FXai indication (Supplementary Table S7). With regard to the type of bleeding, mean total cost per patient was higher in the case of gastrointestinal bleeding, followed by intracranial hemorrhage, other bleeding and trauma-related bleeding. In the case of bleeding-related cost, the order was gastrointestinal bleeding, trauma-related bleeding, other bleeding and intracranial hemorrhage. 

Risk factors for the development of clinical events were specifically analyzed using Cox regression models (myocardial infarction, stroke, cardiovascular mortality and all-cause death). A history of type 2 diabetes increased the risk of myocardial infarction, previous major bleeding the risk of stroke, and a history of coronary artery disease the risk of all-cause death (Supplementary Table S8).

## 5. Discussion

Our data showed that in patients initiating treatment with FXais in clinical practice in Spain, mainly for AF or VTE, around 5% developed a major bleed during follow-up. Despite the high use of replacement agents, the incidence of clinical events was high and one in twenty patients died. Moreover, bleeding-related HCRU and costs were substantially high, hospitalizations being the main determinant.

In Spain, a marked increase has been observed in the use of oral FXais in the last decade (from less than 2% in 2013 to nearly 25% in 2022), but use is lower compared to other European countries, likely due to the restrictions on reimbursement of DOACs, limited to only some specific situations among patients with AF and the lack of reimbursement in the case of patients with VTE [22,23]. 

Our patients were old (mean age 78 years), mainly women (61%) and with many comorbidities (79% had hypertension, one third type 2 diabetes, and 24% heart failure). In the phase 3 clinical trials comparing direct FXai vs. warfarin in an AF population (ROCKET-AF, ARISTOTLE and ENGAGE AF-TIMI 48), the mean age ranged from 70 to 73 years, 35–40% of patients were females, 90–94% had arterial hypertension, 25–40% type 2 diabetes and 35–63% heart failure [24,25,26]. In contrast, patients with VTE included in the phase 3 clinical trials comparing direct FXais vs. standard therapy (EINSTEIN, AMPLIFY and Hokusai-VTE) were younger, and had fewer comorbidities [27,28,29]. In this context, real-life population data are mandatory in order to extend the information given by clinical trials into clinical practice [30]. Of note, the baseline clinical characteristics of our study are in line with those reported by other international studies [27,28,29], confirming the generalizability of our results.

In our study, out of 8972 patients taking direct FXais, 5.2% had a major bleed. In the ROCKET-AF, ARISTOTLE and ENGAGE AF-TIMI 48 trials, 5.6%, 3.6% and 7.5% of patients taking rivaroxaban, apixaban and edoxaban, respectively, had a major bleeding event during follow-up [24,25,26]. In other studies performed in a real-life population, this proportion seemed lower [27,28,29]. In our case, the proportion of patients with major bleeding was similar to that found in clinical trials, but higher than that reported in real-life studies, likely due to a less restrictive definition of major bleeding in our study [31]. The use of the new DOAC score could be useful to stratify patients on the basis of expected bleeding risk [32].

Of note, 70% and 28.1% of patients died after a major trauma-related and intracranial bleeds, respectively, whereas gastrointestinal bleeding was more common (89% of the total) but was associated with lower mortality (2.2%). The mortality related to other major bleeding was also low (10.0%). In the phase 3 clinical trials in an AF population, among patients with a major bleeding event, 32–57% and 14–25% of cases had a gastrointestinal and intracranial origin, respectively [24,25,26]. As a result, although the majority of patients survived after major bleeding in our study, there was still a substantial proportion of patients that presented a fatal evolution, especially patients with trauma and intracranial hemorrhage, despite replacement agents being administered in most cases during the bleeding event (prothrombin complex concentrates [63%], transfusion of blood products [20%] and Factor VIIa [8%]). In addition, surgical procedures used to correct bleeding were performed in nearly 19% of the cases of major bleeding. Remarkably, mortality rates were higher in those patients who were not treated with prothrombin complex concentrates, which were more frequently used in the case of intracranial bleeding. Guidelines recommend in case of non-life-threatening major bleeding among patients treated with FXais, supportive measures (i.e., mechanical compression, endoscopic or surgical hemostasis, fluid replacement, including red transfusion, platelet replacement or consideration of adjuvant tranexamic acid) and the treatment of factors and comorbidities contributing to bleeding. In the case of life-threatening bleeding or bleeding into critical sites, these guidelines recommend the use of andexanet alfa if available, otherwise the use of replacement agents, particularly prothrombin complex concentrates [1,12,33]. Different studies have shown that in cases of major bleeding related to the use of FXais, the use of andexanet alfa can reduce anti-FXa activity, with high hemostatic efficacy in most subjects [34], including a prospective, randomized trial comparing andexanet alfa to usual care (mostly PCCs), resulting in improved hemostatic efficacy and better control of hematoma expansion in FXa inhibitor-related intracerebral hemorrhage [35]. More recently, the ANNEXA-I trial has shown that among patients with intracerebral hemorrhage who were receiving FXai, compared to usual care, andexanet alfa resulted in better control of hematoma expansion. However, an increased risk of thrombotic events was also observed. Despite that, the net clinical benefit clearly favored the use of andexanet alfa in this population [35]. Furthermore, although there may be some differences between reversal agents, it has been reported that the use of andexanet alfa for the reversal of anticoagulation in patients with FXai-related intracranial hemorrhage may be a cost-effective approach [36,37]. In our study, rates of thrombotic events were also high among those patients treated with prothrombin complex concentrates. Unfortunately, at the time we performed our study, andexanet alfa was not available in Spain. Although specific studies are warranted, it is likely that the introduction of andexanet alfa in clinical practice could be associated with a reduction in mortality rates.

Our study showed that after a major bleeding event occurred, not only mortality but also morbidity rates increased. This was particularly relevant in patients with some conditions, such as type 2 diabetes, chronic kidney disease or prior coronary artery disease. Although the incidence rates of clinical events decreased over time, these data indicate that patients with AF or VTE, taking direct FXais, who present with major bleeding are at particularly high risk for clinical events and require a comprehensive management of all comorbidities to reduce the risk of developing complications during follow-up [1,2,3]. 

Our study showed that HCRU, including outpatient visits, laboratory/radiology investigations and hospitalization, was high after major bleeding, with a mean bleeding-related length of hospital stay of 12 days. Previous studies have shown that among patients taking DOACs, HCRU is high after a major bleeding event, but lower when compared to patients taking warfarin that present with a major bleed [13]. In this context, although specific studies are required, the use of a specific reversal agents, such as andexanet alfa with a rapid mechanism of action, could be useful to reduce HCRU after major bleeding. 

The development of major bleeding in anticoagulated patients with either AF or VTE is associated with high healthcare costs [14,38,39]. In our study, the total overall cost reached EUR 7,843,056.4 at year 3, and EUR 5,816,930.5 in the case of bleeding-related costs (mean cost per patient of EUR 16,687.35 and EUR 12,376.45, respectively). Since nearly 80% of bleeding-related costs were accounted for by hospitalizations, and overall prescriptions for only 8% of the total cost, better management of major bleeding, including the use of specific reversal agents, and also the use of DOACs (vs. VKAs), may reduce total costs associated with major bleeding [40]. On the other hand, for comparison purposes, modern data on the clinical and economic burden of patients suffering from major bleeding without anticoagulation would be desirable and are currently lacking.

Our study has some limitations. First, since this was a cohort study, with a retrospective design, only those variables included in the electronic health record could be collected, and some data could be lacking. In addition, as this was a retrospective study, there could be some confounders that may have impacted on the results, limiting the generalizability of our data. Despite that, the high sample size could decrease this limitation. Furthermore, no control group was available, and only indirect comparisons could be performed with other studies. Lastly, our results can only be applied to patients with similar clinical characteristics and in similar healthcare systems.

In conclusion, around 5% of subjects initiating treatment with a direct FXai, mainly for AF or VTE, developed a major bleeding event during follow-up. The management of these patients is challenging. In fact, despite the extended use of replacement agents, the incidence of major clinical events, bleeding-related HCRU and costs during the study period were high, and one in twenty patients died, evidencing the need for new reversal treatment strategies. 

## Figures and Tables

**Table 1:** Baseline characteristics on the day prior to index day 1, according to the type of major bleeding and the use of prothrombin complex concentrates.

	All FXai (N = 470)	Type of Major Bleeding				Use of Prothrombin Complex Concentrates		
		GIB (N = 418)	ICH (N = 32)	Other Bleeding (N = 20)	Trauma Bleeding (N = 10)	With (N = 297)	Without (N = 173)	*p*
Biodemographic data								
Age, years	77.93 (9.71)	77.93 (9.64)	76.48 (9.89)	80.19 (10.79)	79.26 (14.68)	77.51 (10.07)	78.64 (9.04)	0.47
Sex, female	287 (61.06)	254 (60.77)	22 (68.75)	11 (55.00)	7 (70.00)	181 (60.94)	106 (61.27)	0.99
Cardiovascular risk factors								
Hypertension	370 (78.72)	326 (77.99)	27 (84.38)	17 (85)	8 (80.00)	233 (78.45)	137 (79.19)	0.92
Type 1 diabetes	13 (2.77)	12 (2.87)	1 (3.13)	0 (0)	1 (10.00)	8 (2.69)	5 (2.89)	0.98
Type 2 diabetes	159 (33.83)	142 (33.97)	12 (37.5)	5 (25.00)	3 (30.00)	105 (35.35)	54 (31.21)	0.86
Vascular disease								
Heart failure	112 (23.83)	96 (22.97)	7 (21.88)	9 (45.00)	2 (20.00)	73 (24.58)	39 (22.54)	0.85
Chronic kidney disease	81 (17.23)	69 (16.51)	9 (28.13)	3 (15.00)	1 (10.00)	56 (18.86)	25 (14.45)	0.80
Coronary artery disease	72 (15.32)	64 (15.31)	5 (15.63)	3 (15.00)	1 (10.00)	50 (16.84)	22 (12.72)	0.87
Myocardial infarction	38 (8.09)	34 (8.13)	3 (9.38)	1 (5.00)	0 (0)	29 (9.76)	9 (5.20)	0.88
Peripheral artery disease	45 (9.57)	40 (9.57)	2 (6.25)	3 (15.00)	1 (10.00)	23 (7.74)	22 (12.72)	0.84
Cerebrovascular disease	38 (8.09)	33 (7.89)	4 (12.5)	1 (5.00)	1 (10.00)	24 (8.08)	14 (8.09)	0.98
FXai use during the follow-up								
FXai indications								
VTE	83 (17.66)	75 (17.94)	4 (12.5)	4 (20.00)	1 (10.00)	53 (17.85)	30 (17.34)	0.99
AF	367 (78.09)	325 (77.75)	27 (84.38)	15 (75.00)	8 (80.00)	230 (77.44)	137 (79.19)	0.95
Non-mechanical cardiac-valve replacement	20 (4.26)	18 (4.31)	1 (3.13)	1 (5.00)	1 (10.00)	14 (4.71)	6 (3.47)	0.97
Concomitant treatments (within 120 days prior to index)								
Gastroprotective agents	359 (76.38)	318 (76.08)	23 (71.88)	18 (90.00)	8 (80.00)	230 (77.44)	129 (74.57)	0.77
NSAIDs	158 (33.62)	140 (33.49)	10 (31.25)	8 (40.00)	4 (40.00)	108 (36.36)	50 (28.9)	0.72
Antiplatelet drugs	70 (14.89)	60 (14.35)	3 (9.38)	7 (35.00)	1 (10.00)	48 (16.16)	22 (12.72)	0.84
Biochemical parameters								
Hemoglobin, g/dL	11.75 (1.55)	11.75 (1.55)	11.85 (1.48)	11.43 (1.68)	11.34 (1.75)	11.74 (1.51)	11.75 (1.61)	0.99
Platelet count, ×10^3^/μL	234.69 (112.16)	233.08 (110.42)	256.41 (142.57)	233.59 (94.45)	235.79 (105.41)	238.95 (113.96)	227.37 (108.92)	0.02
eGFR, mL/min/1.73 m^2^	93.57 (12.44)	93.66 (12.46)	93.07 (14.96)	92.6 (6.62)	96.17 (5.16)	93.21 (13.86)	94.2 (9.52)	0.78
Creatinine clearance, mL/min	48.42 (22.12)	48.85 (22.05)	42.11 (20.01)	49.68 (26.24)	45.64 (10.8)	48.45 (21)	48.37 (24)	0.97
Actions taken to control bleeding								
Prothrombin complex concentrates	297 (63.19)	263 (62.92)	23 (71.88)	11 (55.00)	7 (70.00)	297 (100)	0 (0)	--
Transfusion of blood products	95 (20.21)	87 (20.81)	6 (18.75)	2 (10.00)	1 (10.00)	48 (16.16)	47 (27.17)	0.57
Factor VIIa	36 (7.66)	32 (7.66)	3 (9.38)	1 (5.00)	0 (0)	26 (8.75)	10 (5.78)	0.91
Fresh frozen plasma	23 (4.89)	21 (5.02)	2 (6.25)	0 (0)	0 (0)	17 (5.72)	6 (3.47)	0.95
Tranexamic acid	15 (3.19)	12 (2.87)	2 (6.25)	1 (5.00)	0 (0)	9 (3.03)	6 (3.47)	0.99
Surgical procedures used to correct bleeding	88 (18.72)	78 (18.66)	7 (21.88)	3 (15.00)	1 (10.00)	63 (21.21)	25 (14.45)	0.95

Quantitative variables are presented as mean and (standard deviation); qualitative variables are presented as absolute and relative (%) frequencies. AF: atrial fibrillation; eGFR: estimated glomerular filtration rate; FXai; Factor Xa inhibitors; GIB: gastrointestinal bleeding; ICH: intracranial hemorrhage; NSAIDs: non-steroidal anti-inflammatory drugs; VTE: venous thromboembolism.

**Table 2:** Cumulative clinical outcomes in the overall study population and according to the use of prothrombin complex concentrates.

Time Window ^1^	All FXais (N = 470)			All FXais with Prothrombin Complex Concentrates (N = 297)			All FXais without Prothrombin Complex Concentrates (N = 173)			*p* for Incidence Rates
	n	%	Incidence Rates (95% CI) ^2^	n	%	Incidence Rates (95% CI) ^2^	n	%	Incidence Rates (95% CI) ^2^	
3 Months since index date										
Death from any cause	36	7.66	32.71 (28.47–36.95)	22	7.41	31.54 (26.26–36.82)	14	8.09	34.74 (27.64–41.84)	0.33
Within index hospitalization	20	4.26	18.17 (14.68–21.66)	12	4.04	17.20 (12.91–21.49)	8	4.62	19.85 (13.91–25.79)	0.22
Cardiovascular death	28	5.96	25.44 (21.5–29.38)	17	5.72	24.37 (19.49–29.25)	11	6.36	27.29 (20.65–33.93)	0.28
Within index hospitalization	20	4.26	18.17 (14.68–21.66)	12	4.04	17.20 (12.91–21.49)	8	4.62	19.85 (13.91–25.79)	0.22
AMI	25	5.32	23.22 (19.4–27.04)	15	5.05	22.03 (17.31–26.75)	10	5.78	25.28 (18.8–31.76)	0.20
Within index hospitalization	5	1.06	4.64 (2.74–6.54)	2	0.67	2.94 (1.02–4.86)	3	1.73	7.59 (3.64–11.54)	0.04
Ischemic stroke	22	4.68	20.42 (16.78–24.06)	14	4.71	20.58 (15.98–25.18)	8	4.62	20.14 (14.16–26.12)	0.85
Within index hospitalization	1	0.21	0.93 (0.06–1.8)	1	0.34	1.47 (0.10–2.84)	0	0.00	0 (0–0)	--
Acute kidney failure	11	2.34	10.07 (7.35–12.79)	7	2.36	10.09 (6.66–13.52)	4	2.31	10.05 (5.57–14.53)	0.98
Within index hospitalization	1	0.21	0.92 (0.06–1.78)	0	0.00	0 (0–0)	1	0.58	2.51 (0.18–4.84)	0.09
Acute liver failure	1	0.21	0.91 (0.05–1.77)	1	0.34	1.43 (0.08–2.78)	0	0.00	0 (0–0)	--
Within index hospitalization	0	0.00	0 (0–0)	0	0.00	0 (0–0)	0	0.00	0 (0–0)	--
3 Years since index date										
Death from any cause	136	28.94	16.42 (13.07–19.77)	88	29.63	16.95 (12.68–21.22)	48	27.75	15.53 (10.13–20.93)	0.62
Cardiovascular death	77	16.38	9.3 (6.67–11.93)	46	15.49	8.86 (5.63–12.09)	31	17.92	10.03 (5.55–14.51)	0.56
AMI	58	12.34	7.34 (4.98–9.7)	35	11.78	7.07 (4.15–9.99)	23	13.29	7.78 (3.79–11.77)	0.72
Ischemic stroke	43	9.15	5.42 (3.37–7.47)	31	10.44	6.31 (3.54–9.08)	12	6.94	3.98 (1.07–6.89)	0.18
Acute kidney failure	33	7.02	4.17 (2.36–5.98)	21	7.07	4.23 (1.94–6.52)	12	6.94	4.07 (1.13–7.01)	0.91
Acute liver failure	8	1.70	0.97 (0.08–1.86)	4	1.35	0.78 (0–1.78)	4	2.31	1.30 (0–2.99)	0.47

Incidence rates (95 confidence interval): per 100 person-years; qualitative variables are presented as absolute and relative (%) frequencies; AMI: acute myocardial infarction; FXai; Factor Xa inhibitor. 1. Time window: cumulative events from index date (day of the first major bleeding); 2. Incidence rate defined as the total number of incident events of interest divided by the total person time at risk.

**Table 3:** Cumulative outpatient visits and hospitalization for 3 years from index date.

Time Window ^1^	All FXais (N = 470)			
	Patients (n)	Visits (n)	% of Patients	Rate, Number of Visits per 100 Patient-Years (95 CI)
	Cumulative HCRU			
	6 Months since index date			
	All-cause HCRU			
Outpatient visits ^2^	444	4313	94.47	1993.62 (1632.42–2354.82)
GPs visits	432	3234	91.91	1494.87 (1172.5–1817.24)
Specialist visits	382	1079	81.28	498.75 (453.55–543.95)
Investigations ^3^	470	1271	100.00	587.50 (542.99–632.01)
Hospitalization	470	579	100.00	267.63 (227.6–307.66)
	Bleeding-related HCRU			
Outpatient visits	444	3953	94.47	1827.22 (1477.85–2176.59)
GPs visits	432	2894	91.91	1337.71 (1029.96–1645.46)
Specialist visits	382	1059	81.28	489.51 (444.32–534.7)
Investigations ^3^	470	838	100.00	387.35 (343.31–431.39)
Hospitalization	470	486	100.00	224.65 (186.92–262.38)
	3 Years since index date			
	All-cause HCRU			
Outpatient visits	450	11,909	95.74	1437.8 (1120.59–1755.01)
GPs visits	450	9160	95.74	1105.91 (822.37–1389.45)
Specialist visits	450	2749	95.74	331.89 (289.32–374.46)
Investigations ^3^	470	2866	100.00	346.02 (303.01–389.03)
Hospitalization	470	690	100.00	83.31 (79.94–86.68)
	Bleeding-related HCRU			
Outpatient visits	450	7444	95.74	898.73 (871.46–926)
GPs visits	450	5535	95.74	668.25 (625.68–710.82)
Specialist visits	443	1909	94.26	230.48 (192.41–268.55)
Investigations ^3^	470	1792	100.00	216.35 (179.12–253.58)
Hospitalization	470	530	100.00	63.99 (59.65–68.33)

1. Index date: day of the first major bleed; 2. Outpatient visits: include GP visits and specialized visits; 3. Laboratory/radiology investigations. Qualitative variables are presented as absolute and relative (%) frequencies. Rate (95% CI): per 100 person-years. FXais; Factor Xa inhibitors; GP: general practitioners; HCRU: healthcare resource utilization.

**Table 4:** Cumulative length of hospital stays, number of prescriptions and work absences for 3 years from index date.

Time Window ^1^	All FXais N = 470			
	Patient with Use of Resources (n)	% of Patients	All Patients Within Cohort	
Cumulative HCRU per Patient			Average	Standard Deviation
	6 Months since Index Date			
	All-cause HCRU			
Length of hospital stays (days)	470	100	13.28	5.99
Number of prescriptions	470	100	8.52	3.90
Work absences—all (days)	40	9	2.56	9.34
	Bleeding-related HCRU			
Length of hospital stays (days)	470	100	11.16	2.79
Number of prescriptions—all	470	100	2.88	0.78
Work absences (days)	40	9	2.22	7.51
	3 Years since index date			
	All-cause HCRU			
Length of hospital stays (days)	470	100	15.82	8.41
Number of prescriptions	470	100	34.27	7.95
Work absences (number of days)	40	9	3.04	11.27
	Bleeding-related HCRU			
Length of hospital stays (days)	470	100	12.20	4.81
Number of prescriptions	470	100	3.86	1.64
Work absences (days)	40	9	2.49	8.65

1. Index date: day of the first major bleed. Quantitative variables are presented as mean and standard deviation; qualitative variables are presented as absolute and relative (%) frequencies. FXais; Factor Xa inhibitors; HCRU: healthcare resource utilization.

**Table 5:** Cumulative costs for 3 years from index date.

Time Window ^1^	All FXais			
Cumulative Costs	No. Patients	Total Cost (EUR)	Mean Costs per Patient (EUR)	SD Costs per Patient (EUR)
	6 Months since index date			
	All-cause costs			
Outpatient	470	514,415.8	1094.50	515.15
GP visit	470	207,364.1	441.20	254.17
Specialist visit	470	255,281.0	543.15	337.41
Investigations ^2^ (outpatient)	470	21,624.8	46.01	36.89
Prescriptions (outpatient)	470	29,764.9	63.33	39.58
Inpatient (hospital + all other costs in hospitalization)	470	4,936,654.7	10,503.52	4325.64
Hospitalizations (>24 h)	470	4,478,320.5	9528.34	4297.66
Investigations ^2^ (within hospital)	470	73,699.1	156.81	131.41
Prescriptions (within hospital)	470	383,745.1	816.48	578.34
Pharmacy and investigation (global)	470	508,834.0	1082.63	604.74
Prescriptions (global)	470	413,510.0	879.81	581.25
Investigations ^2^ (global)	470	95,324.0	202.82	142.91
Indirect cost				
Cost of absence from work	470	329,132.7	700.28	2558.70
Total overall cost	470	5,780,203.3	12,298.30	4855.57
	Bleeding-related costs			
Outpatient	470	450,116.4	957.69	479.22
GP visits	470	185,563.3	394.82	223.03
Specialist visits	470	250,549.2	533.08	323.50
Investigations ^2^ (outpatient)	470	12,997.6	27.65	40.89
Prescriptions (outpatient)	470	777.4	1.65	3.91
Inpatient—bleeding episode (hospital + all other costs in bleeding event hospitalization)	470	4,194,794.0	8925.09	2135.64
Hospitalizations (>24 h)	470	3,764,229.4	8009.00	2000.76
Investigations ^2^ (within hospital)	470	46,641.3	99.24	78.59
Prescriptions (within hospital)	470	383,314.3	815.56	578.42
Pharmacy and investigation (global)	470	443,730.6	944.11	584.17
Prescriptions	470	384,091.7	817.22	578.73
Investigations ^2^ (global)	470	59,638.9	126.89	86.12
Indirect cost				
Cost of absence from work	470	285,558.9	607.57	2056.87
Total overall bleeding cost	470	4,930,469.4	10,490.36	2587.72
	3 Years since index date			
	All-cause costs			
Outpatient	470	1,413,481.1	3007.41	1224.13
GP visit	470	587,339.2	1249.66	631.16
Specialist visit	470	650,386.8	1383.80	674.18
Investigations ^2^ (outpatient)	470	53,693.2	114.24	57.42
Prescriptions (outpatient)	470	121,115.9	257.69	113.70
Inpatient	470	6,038,507.6	12,847.89	6869.78
Hospitalizations (>24 h)	470	5,335,947.6	11,353.08	6038.97
Investigations ^2^ (within hospital)	470	137,655.3	292.88	200.22
Prescriptions (within hospital)	470	562,984.6	1197.84	1895.53
Pharmacy and investigation (global)	470	875,449.0	1862.66	1946.94
Prescriptions (global)	470	684,100.5	1455.53	1901.91
Investigations ^2^ (global)	470	191,348.5	407.12	230.74
Indirect cost				
Cost of absence from work	470	391,067.8	832.06	3089.81
Total overall cost	470	7,843,056.4	16,687.35	7661.35
	Bleeding-related costs			
Outpatient	470	857,818.3	1825.15	880.36
GP visits	470	354,904.2	755.12	368.42
Specialist visits	470	451,650.9	960.96	550.29
Investigations ^2^ (outpatient)	470	37,857.6	80.55	136.09
Prescriptions (outpatient)	470	13,405.5	28.52	16.11
Inpatient—bleeding episode (hospital + all other costs in bleeding event hospitalization)	470	4,638,200.9	9868.51	3779.94
Hospitalizations (>24 h)	470	4,113,739.3	8752.64	3451.91
Investigations ^2^ (within hospital)	470	79,401.1	168.94	113.76
Prescriptions (within hospital)	470	443,935.5	944.54	703.76
Pharmacy and investigation (global)	470	574,599.8	1222.55	736.63
Prescriptions	470	457,341.0	973.07	703.98
Investigations ^2^ (global)	470	117,258.8	249.49	204.30
Indirect cost				
Cost of absence from work	470	320,911.2	682.79	2371.46
Total overall bleeding cost	470	5,816,930.5	12,376.45	4259.28

1. Index date: day of the first major bleed; 2. Laboratory/radiology investigations. Quantitative variables are presented as mean and (standard deviation); qualitative variables are presented by their absolute frequencies; FXais: Factor Xa inhibitors; GP: general practitioner.

## Data Availability

This was a secondary data study using the BIG-PACR database, and the data can be obtained upon reasonable request.

## Supplementary Materials

The following supporting information can be downloaded at: https://www.mdpi.com/article/10.3390/jcm13144253/s1, Table S1: Costs 2023. Table S2: Baseline characteristics on the day prior to index day 1 in the overall population and according to the use of pro-thrombin complex concentrates, the type of FXai and the FXai indication. Table S3: CHA_2_DS_2_-VASc and HAS-BLED scores on the day prior to index date. Table S4: Cumulative clinical outcomes in the overall study population and according to the use of prothrombin complex concentrates, the type of first major bleeding, the type of FXai and the FXai indication. Table S5: Cumulative outpatient Visits and Hospitalization for 3 years from index date in the overall study population and according to the type of first major bleeding, the type of FXai and the FXai indication. Table S6: Cumulative Length of hospital stays, number of prescriptions and work absences for 3 years from index date in the overall study population and according to the type of first major bleeding, the type of FXai and the FXai indication. Table S7: Cumulative costs for 3 years from index date in the overall study population and according to the type of the first major bleeding, the type of FXai and the FXai indication. Table S8: Independent variables associated with outcomes (Fine and Gray regression models in the case of myocardial infarction and stroke and Cox regression models in the case of overall or all-cause mortality).

## References

[1] Hindricks G., Potpara T., Dagres N., Arbelo E., Bax J.J., Blomström-Lundqvist C., Boriani G., Castella M., Dan G.A., Dilaveris P.E. (2021). 2020 ESC Guidelines for the diagnosis and management of atrial fibrillation developed in collaboration with the European Association for Cardio-Thoracic Surgery (EACTS): The Task Force for the diagnosis and management of atrial fibrillation of the European Society of Cardiology (ESC) Developed with the special contribution of the European Heart Rhythm Association (EHRA) of the ESC. Eur. Heart J..

[2] Joglar J.A., Chung M.K., Armbruster A.L., Benjamin E.J., Chyou J.Y., Cronin E.M., Deswal A., Eckhardt L.L., Goldberger Z.D., Gopinathannair R. (2024). 2023 ACC/AHA/ACCP/HRS Guideline for the Diagnosis and Management of Atrial Fibrillation: A Report of the American College of Cardiology/American Heart Association Joint Committee on Clinical Practice Guidelines. Circulation.

[3] Ortel T.L., Neumann I., Ageno W., Beyth R., Clark N.P., Cuker A., Hutten B.A., Jaff M.R., Manja V., Schulman S. (2020). American Society of Hematology 2020 guidelines for management of venous thromboembolism: Treatment of deep vein thrombosis and pulmonary embolism. Blood Adv..

[4] Cheng A., Malkin C., Briffa N.P. (2023). Antithrombotic therapy after heart valve intervention: Review of mechanisms, evidence and current guidance. Heart.

[5] Ruff C.T., Giugliano R.P., Braunwald E., Hoffman E.B., Deenadayalu N., Ezekowitz M.D., Camm A.J., Weitz J.I., Lewis B.S., Parkhomenko A. (2014). Comparison of the efficacy and safety of new oral anticoagulants with warfarin in patients with atrial fibrillation: A meta-analysis of randomised trials. Lancet.

[6] van der Hulle T., Kooiman J., den Exter P.L., Dekkers O.M., Klok F.A., Huisman M.V. (2014). Effectiveness and safety of novel oral anticoagulants as compared with vitamin K antagonists in the treatment of acute symptomatic venous thromboembolism: A systematic review and meta-analysis. J. Thromb. Haemost..

[7] Almutairi A.R., Zhou L., Gellad W.F., Lee J.K., Slack M.K., Martin J.R., Lo-Ciganic W.H. (2017). Effectiveness and Safety of Non-vitamin K Antagonist Oral Anticoagulants for Atrial Fibrillation and Venous Thromboembolism: A Systematic Review and Meta-analyses. Clin. Ther..

[8] Lamberts M., Staerk L., Olesen J.B., Fosbøl E.L., Hansen M.L., Harboe L., Lefevre C., Evans D., Gislason G.H. (2017). Major Bleeding Complications and Persistence With Oral Anticoagulation in Non-Valvular Atrial Fibrillation: Contemporary Findings in Real-Life Danish Patients. J. Am. Heart Assoc..

[9] Zhang Q., Wang R., Chen L., Chen W. (2024). Effect of China national centralized drug procurement policy on anticoagulation selection and hemorrhage events in patients with AF in Suining. Front. Pharmacol..

[10] O’Brien E.C., Holmes D.N., Thomas L., Fonarow G.C., Kowey P.R., Ansell J.E., Mahaffey K.W., Gersh B.J., Peterson E.D., Piccini J.P. (2018). Therapeutic Strategies Following Major, Clinically Relevant Nonmajor, and Nuisance Bleeding in Atrial Fibrillation: Findings From ORBIT-AF. J. Am. Heart Assoc..

[11] De Marco F., Valli G., Ancona C., Ruggieri M.P. (2023). Management of bleeding in patients on direct oral anticoagulants in emergency department: Where we are and where we are going. Eur. Heart J. Suppl..

[12] Steffel J., Collins R., Antz M., Cornu P., Desteghe L., Haeusler K.G., Oldgren J., Reinecke H., Roldan-Schilling V., Rowell N. (2021). 2021 European Heart Rhythm Association Practical Guide on the Use of Non-Vitamin K Antagonist Oral Anticoagulants in Patients with Atrial Fibrillation. Ep Eur..

[13] Xu Y., Schulman S., Dowlatshahi D., Holbrook A.M., Simpson C.S., Shepherd L.E., Wells P.S., Giulivi A., Gomes T., Mamdani M. (2019). Healthcare resource utilization and costs among patients with direct oral anticoagulant or warfarin-related major bleeding. Thromb. Res..

[14] Deitelzweig S.B., Lovelace B., Christoph M., Lingohr-Smith M., Lin J., Fermann G.J. (2020). Evaluation of the Incremental Healthcare Economic Burden of Patients with Atrial Fibrillation Treated with Direct-Acting Oral Anticoagulants and Hospitalized for Major Bleeds in the USA. Adv. Ther..

[15] Escobar C., Palacios B., Villarreal M., Gutiérrez M., Capel M., Hernández I., García M., Lledó L., Arenillas J.F. (2024). Clinical Characteristics and Incidence of Hemorrhagic Complications in Patients Taking Factor Xa Inhibitors in Spain: A Long-Term Observational Study. J. Clin. Med..

[16] Escobar C., Palacios B., Gonzalez V., Gutiérrez M., Duong M., Chen H., Justo N., Cid-Ruzafa J., Hernández I., Hunt P.R. (2023). Burden of Illness beyond Mortality and Heart Failure Hospitalizations in Patients Newly Diagnosed with Heart Failure in Spain According to Ejection Fraction. J. Clin. Med..

[17] Sicras-Mainar A., Sicras-Navarro A., Palacios B., Varela L., Delgado J.F. (2022). Epidemiology and treatment of heart failure in Spain: The HF-PATHWAYS study. Rev. Esp. Cardiol. (Engl. Ed.).

[18] Escobar C., Aranda U., Palacios B., Capel M., Sicras A., Sicras A., Hormigo A., Alcázar R., Manito N., Botana M. (2021). Epidemiology, clinical profile, management, and two-year risk complications among patients with chronic kidney disease in Spain. Nefrologia (Engl. Ed.).

[19] (2007). Database of Spanish Healthcare Costs and Cost-Effectiveness Ratios: eSalud.

[20] BOTPLUS Database General Council of Pharmacist Colleges. https://botplusweb.farmaceuticos.com/.

[21] Costs of Absence from Work in Spain. https://www.ine.es/dynt3/inebase/index.htm?padre=4563&capsel=4563.

[22] Llisterri Caro J.L., Cinza-Sanjurjo S., Polo Garcia J., Prieto Díaz M.A. (2019). Utilización de los anticoagulantes orales de acción directa en Atención Primaria de España. Posicionamiento de SEMERGEN ante la situación actual [Use of direct-acting oral anticoagulants in Primary Care in Spain. Positioning statement by SEMERGEN on the current situation]. Semergen.

[23] General Criteria and Recommendations for the Use of Direct Oral Anticoagulants (DOACs) in the Prevention of Stroke and Systemic Embolism in Patients with Non-Valvular Atrial Fibrillation. Therapeutic Positioning Report. IPT-230/V5/08022024. Spanish Agency for Medicines and Health Products. Ministry of Health, Published on 8 February 2024. https://www.aemps.gob.es/medicamentosUsoHumano/informesPublicos/docs/2024/IPT-230-ACOD-FANV.pdf.

[24] Patel M.R., Mahaffey K.W., Garg J., Pan G., Singer D.E., Hacke W., Breithardt G., Halperin J.L., Hankey G.J., Piccini J.P. (2011). Rivaroxaban versus warfarin in nonvalvular atrial fibrillation. N. Engl. J. Med..

[25] Granger C.B., Alexander J.H., McMurray J.J., Lopes R.D., Hylek E.M., Hanna M., Al-Khalidi H.R., Ansell J., Atar D., Avezum A. (2011). Apixaban versus warfarin in patients with atrial fibrillation. N. Engl. J. Med..

[26] Giugliano R.P., Ruff C.T., Braunwald E., Murphy S.A., Wiviott S.D., Halperin J.L., Waldo A.L., Ezekowitz M.D., Weitz J.I., Špinar J. (2013). Edoxaban versus warfarin in patients with atrial fibrillation. N. Engl. J. Med..

[27] Bauersachs R., Berkowitz S.D., Brenner B., Buller H.R., Decousus H., Gallus A.S., Lensing A.W., Misselwitz F., Prins M.H., Raskob G.E. (2010). Oral rivaroxaban for symptomatic venous thromboembolism. N. Engl. J. Med..

[28] Agnelli G., Buller H.R., Cohen A., Curto M., Gallus A.S., Johnson M., Masiukiewicz U., Pak R., Thompson J., Raskob G.E. (2013). Oral apixaban for the treatment of acute venous thromboembolism. N. Engl. J. Med..

[29] Büller H.R., Décousus H., Grosso M.A., Mercuri M., Middeldorp S., Prins M.H., Raskob G.E., Schellong S.M., Schwocho L., Segers A. (2013). Edoxaban versus warfarin for the treatment of symptomatic venous thromboembolism. N. Engl. J. Med..

[30] Roberti R., Iannone L.F., Palleria C., Curcio A., Rossi M., Sciacqua A., Armentaro G., Vero A., Manti A., Cassano V. (2021). Direct Oral Anticoagulants: From Randomized Clinical Trials to Real-World Clinical Practice. Front. Pharmacol..

[31] Schulman S., Kearon C., Subcommittee on Control of Anticoagulation of the Scientific and Standardization Committee of the International Society on Thrombosis and Haemostasis (2005). Definition of major bleeding in clinical investigations of antihemostatic medicinal products in non-surgical patients. J. Thromb. Haemost..

[32] Aggarwal R., Ruff C.T., Virdone S., Perreault S., Kakkar A.K., Palazzolo M.G., Dorais M., Kayani G., Singer D.E., Secemsky E. (2023). Development and Validation of the DOAC Score: A Novel Bleeding Risk Prediction Tool for Patients With Atrial Fibrillation on Direct-Acting Oral Anticoagulants. Circulation.

[33] Grottke O., Afshari A., Ahmed A., Arnaoutoglou E., Bolliger D., Fenger-Eriksen C., von Heymann C. (2024). Clinical guideline on reversal of direct oral anticoagulants in patients with life threatening bleeding. Eur. J. Anaesthesiol..

[34] Milling T.J., Middeldorp S., Xu L., Koch B., Demchuk A., Eikelboom J.W., Verhamme P., Cohen A.T., Beyer-Westendorf J., Gibson C.M. (2023). Final Study Report of Andexanet Alfa for Major Bleeding With Factor Xa Inhibitors. Circulation.

[35] Connolly S.J., Sharma M., Cohen A.T., Demchuk A.M., Członkowska A., Lindgren A.G., Molina C.A., Bereczki D., Toni D., Seiffge D.J. (2024). Andexanet for Factor Xa Inhibitor-Associated Acute Intracerebral Hemorrhage. N. Engl. J. Med..

[36] Fanikos J., Goldstein J.N., Lovelace B., Beaubrun A.C., Blissett R.S., Aragão F. (2022). Cost-effectiveness of andexanet alfa versus four-factor prothrombin complex concentrate for the treatment of oral factor Xa inhibitor-related intracranial hemorrhage in the US. J. Med. Econ..

[37] Spyropoulos A.C., Hartaigh B.Ó., Cao Z., Caberwal H., Lipkin C., Petrini M., Wang C. (2022). Costs and Healthcare Resource Utilization Associated with Idarucizumab or Andexanet Alfa Oral Anticoagulant Reversal in Patients Hospitalized with Life-Threatening Bleeds. Clin. Appl. Thromb. Hemost..

[38] Lutsey P.L., MacLehose R.F., Claxton J.S., Walker R.F., Adam T.J., Alonso A., Zakai N.A. (2020). Impact of oral anticoagulation choice on healthcare utilization for the primary treatment of venous thromboembolism. Vasc. Med..

[39] Mokgokong R., Khachatryan A., Quignot N., Chaves J., Moniot A., Gusto G. (2022). Comparative Analysis of All-Cause Health Care Resource Utilization and Costs Among Venous Thrombosis Patients Without Cancer Prescribed Apixaban or VKAs in France. Adv. Ther..

[40] Choi J.H., Kim W., Kim Y.T., Cho J., Shin S.Y., Kim C., Kim J.B. (2022). Cost-effectiveness of Direct Oral Anticoagulant vs. Warfarin Among Atrial Fibrillation Patients With Intermediate Stroke Risk. Front. Cardiovasc. Med..

