# Supplementary material for: Clinical and Economic Consequences of a First Major Bleeding Event in Patients Treated with Direct Factor Xa Inhibitors in Spain: A Long-Term Observational Study
Source: J Clin Med. 2024 Jul 21;13(14):4253. doi: 10.3390/jcm13144253 (PMC11278163; doi:10.3390/jcm13144253)
Supplement: Supplementary file 1 [file jcm-13-04253-s001.zip › jcm-3068085-supplementary.pdf]

Supplementary Table S1. Costs 2023.

**Outpatient lvisits:**	
GP visit	64.12
Specialistvisit*	236.59
Hospital (stay / day)	717.68
**Investigations:**	
Laboratory tests	56.76
Conventional radiology	46.15
Computed tomography	212.29
Magnetic nuclear resonance	356.32
**Indirect costs:**	
Cost per day not worked	274.05
Pharmaceutical prescription	RP+VAT

*Cardiologists, Internal Medicine Physicians, Neurologists, Gastroenterologists, Intensivists, Oncologists, Orthopedic Surgeons

Cost data was inferred from the eSalud Consulting database (Database of Spanish healthcare costs and cost-effectiveness ratios: eSalud [Internet]. Barcelona: Oblikue Consulting, S.L; 2007 [last update: 2018; consultation date: 15/02/2024]. Available at: http://esalud.oblikue.com/). Pharmaceutical prescription costs were based on the full price of product (BOTPLUS Database General Council of Pharmacist Colleges. Available at: https://botplusweb.farmaceuticos.com/). Costs of absence from work were calculated by multiplying the number of days of absence from work due to sickness by the mean daily salary of a working person in Spain (available at https://www.ine.es/dynt3/inebase/index.htm?padre=4563&capsel=4563; Accessed: 15/02/2024).

Supplementary Table S2. Baseline characteristics on the day prior to index day 1 in the overall population and according to the use of pro-thrombin complex concentrates, the type of FXai and the FXai indication.

Overall population and according to the use of pro-thrombin complex concentrates.

	All FXai (N=470)	Type of major bleeding				Use of prothrombin complex concentrates	
		GIB (N=418)	ICH (N=32)	Other bleeding (N=20)	Trauma bleeding (N=10)	With (N=297)	Without (N=173)
**Biodemographic data**							
Age, years	77.93 (9.71)	77.93 (9.64)	76.48 (9.89)	80.19 (10.79)	79.26 (14.68)	77.51 (10.07)	78.64 (9.04)
<45 years	0 (0)	0 (0)	0 (0)	0 (0)	0 (0)	0 (0)	0 (0)
45-64 years	40 (8.51)	34 (8.13)	4 (12.50)	2 (10.00)	3 (30.00)	29 (9.76)	11 (6.36)
65-74 years	140 (29.79)	121 (28.95)	14 (43.75)	5 (25.00)	2 (20.00)	94 (31.65)	46 (26.59)
75-84 years	183 (38.94)	168 (40.19)	9 (28.13)	6 (30.00)	1 (10.00)	108 (36.36)	75 (43.35)
≥85 years	107 (22.77)	95 (22.73)	5 (15.63)	7 (35.00)	4 (40.00)	66 (22.22)	41 (23.70)
Sex, female	287 (61.06)	254 (60.77)	22 (68.75)	11 (55.00)	7 (70.00)	181 (60.94)	106 (61.27)
BMI	27.49 (5.24)	27.44 (5.2)	27.94 (5.45)	27.87 (6.05)	28.34 (6.29)	27.61 (5.13)	27.29 (5.44)
Alcohol use	4 (0.85)	4 (0.96)	0 (0)	0 (0)	0 (0)	2 (0.67)	2 (1.16)
Updated Charlson comorbidity index	7.23 (2.13)	7.20 (2.12)	7.47 (2.41)	7.45 (2.01)	7.50 (2.42)	7.35 (2.23)	7.02 (1.94)
**Cardiovascular risk factors**							
Hypertension	370 (78.72)	326 (77.99)	27 (84.38)	17 (85)	8 (80.00)	233 (78.45)	137 (79.19)
Hypercholesterolemia	223 (47.45)	202 (48.33)	10 (31.25)	11 (55)	4 (40.00)	141 (47.47)	82 (47.40)
Type 1 diabetes	13 (2.77)	12 (2.87)	1 (3.13)	0 (0)	1 (10.00)	8 (2.69)	5 (2.89)
Type 2 diabetes	159 (33.83)	142 (33.97)	12 (37.5)	5 (25.00)	3 (30.00)	105 (35.35)	54 (31.21)
Smoking	4 (0.85)	4 (0.96)	0 (0)	0 (0)	0 (0)	3 (1.01)	1 (0.58)
**Vascular disease**							
Heart failure	112 (23.83)	96 (22.97)	7 (21.88)	9 (45.00)	2 (20.00)	73 (24.58)	39 (22.54)
Chronic kidney disease	81 (17.23)	69 (16.51)	9 (28.13)	3 (15.00)	1 (10.00)	56 (18.86)	25 (14.45)
Coronary artery disease	72 (15.32)	64 (15.31)	5 (15.63)	3 (15.00)	1 (10.00)	50 (16.84)	22 (12.72)
Myocardial infarction	38 (8.09)	34 (8.13)	3 (9.38)	1 (5.00)	0 (0)	29 (9.76)	9 (5.20)
Peripheral artery disease	45 (9.57)	40 (9.57)	2 (6.25)	3 (15.00)	1 (10.00)	23 (7.74)	22 (12.72)
Cerebrovascular disease	38 (8.09)	33 (7.89)	4 (12.5)	1 (5.00)	1 (10.00)	24 (8.08)	14 (8.09)
**Conditions that increase the risk of bleeding**							
Cancer	53 (11.28)	47 (11.24)	3 (9.38)	3 (15.00)	1 (10.00)	32 (10.77)	21 (12.14)
Anemia	93 (19.79)	82 (19.62)	7 (21.88)	4 (20.00)	2 (20.00)	64 (21.55)	29 (16.76)
Surgical procedures within 30 days prior to bleeding event	74 (15.74)	69 (16.51)	2 (6.25)	3 (15.00)	1 (10.00)	50 (16.84)	24 (13.87)
Major bleeding history >60 days prior to FXai start	50 (10.64)	45 (10.77)	3 (9.38)	2 (10.00)	1 (10.00)	36 (12.12)	14 (8.09)
Trauma within 30 days prior to bleeding event	34 (7.23)	30 (7.18)	1 (3.13)	3 (15.00)	0 (0)	20 (6.73)	14 (8.09)
Liver disease	26 (5.53)	23 (5.5)	2 (6.25)	1 (5.00)	0 (0)	19 (6.40)	7 (4.05)
Peptic ulcer disease	28 (5.96)	24 (5.74)	2 (6.25)	2 (10.00)	0 (0)	14 (4.71)	14 (8.09)

**FXai use during the follow-up**							
Year of first FXai use							
2013	6 (1.28)	5 (1.2)	0 (0)	1 (5.00)	1 (10.00)	4 (1.35)	2 (1.16)
2014	16 (3.4)	16 (3.83)	0 (0)	0 (0)	0 (0)	10 (3.37)	6 (3.47)
2015	18 (3.83)	16 (3.83)	0 (0)	2 (10)	0 (0)	13 (4.38)	5 (2.89)
2016	12 (2.55)	11 (2.63)	1 (3.13)	0 (0)	0 (0)	9 (3.03)	3 (1.73)
2017	33 (7.02)	30 (7.18)	3 (9.38)	0 (0)	0 (0)	20 (6.73)	13 (7.51)
2018	53 (11.28)	48 (11.48)	3 (9.38)	2 (10.00)	0 (0)	34 (11.45)	19 (10.98)
2019	50 (10.64)	46 (11)	2 (6.25)	2 (10.00)	0 (0)	30 (10.1)	20 (11.56)
2020	63 (13.4)	55 (13.16)	3 (9.38)	5 (25.00)	4 (40.00)	41 (13.8)	22 (12.72)
2021	100 (21.28)	91 (21.77)	7 (21.88)	2 (10.00)	2 (20.00)	61 (20.54)	39 (22.54)
2022	119 (25.32)	100 (23.92)	13 (40.63)	6 (30.00)	3 (30.00)	75 (25.25)	44 (25.43)
FXai indications							
VTE	83 (17.66)	75 (17.94)	4 (12.5)	4 (20.00)	1 (10.00)	53 (17.85)	30 (17.34)
AF	367 (78.09)	325 (77.75)	27 (84.38)	15 (75.00)	8 (80.00)	230 (77.44)	137 (79.19)
Non-mechanical cardiac-valve replacement	20 (4.26)	18 (4.31)	1 (3.13)	1 (5.00)	1 (10).00	14 (4.71)	6 (3.47)
**Concomitant treatments (within 120 days prior to index)**							
Gastroprotective agents	359 (76.38)	318 (76.08)	23 (71.88)	18 (90.00)	8 (80.00)	230 (77.44)	129 (74.57)
Antihypertensive therapies	370 (78.72)	326 (77.99)	27 (84.38)	17 (85.00)	8 (80.00)	233 (78.45)	137 (79.19)
Lipid lowering therapies	297 (63.19)	265 (63.4)	16 (50)	16 (80.00)	5 (50.00)	192 (64.65)	105 (60.69)
Antidiabetic drugs	171 (36.38)	153 (36.6)	13 (40.63)	5 (25.00)	4 (40.00)	112 (37.71)	59 (34.1)
NSAIDs	158 (33.62)	140 (33.49)	10 (31.25)	8 (40.00)	4 (40.00)	108 (36.36)	50 (28.9)
Antiplatelet drugs	70 (14.89)	60 (14.35)	3 (9.38)	7 (35.00)	1 (10.00)	48 (16.16)	22 (12.72)
Antidepressants	62 (13.19)	54 (12.92)	3 (9.38)	5 (25.00)	3 (30.00)	38 (12.79)	24 (13.87)
Anticancer drugs*	37 (7.87)	33 (7.89)	3 (9.38)	1 (5.00)	1 (10.00)	20 (6.73)	17 (9.83)
**Biochemical parameters**							
Hemoglobin, g/dL	11.75 (1.55)	11.75 (1.55)	11.85 (1.48)	11.43 (1.68)	11.34 (1.75)	11.74 (1.51)	11.75 (1.61)
HbA1c,	7.44 (1.18)	7.45 (1.19)	7.33 (1.17)	7.47 (1.03)	7.37 (1.16)	7.44 (1.2)	7.45 (1.16)
Platelet count, ×103/μL	234.69 (112.16)	233.08 (110.42)	256.41 (142.57)	233.59 (94.45)	235.79 (105.41)	238.95 (113.96)	227.37 (108.92)
eGFR, mL/min/1.73 m^2	93.57 (12.44)	93.66 (12.46)	93.07 (14.96)	92.6 (6.62)	96.17 (5.16)	93.21 (13.86)	94.2 (9.52)
Creatinine clearance, mL/min	48.42 (22.12)	48.85 (22.05)	42.11 (20.01)	49.68 (26.24)	45.64 (10.8)	48.45 (21)	48.37 (24)
**Actions taken to control bleeding**							
Prothrombin complex concentrates	297 (63.19)	263 (62.92)	23 (71.88)	11 (55.00)	7 (70.00)	297 (100)	0 (0)
Transfusion of blood products	95 (20.21)	87 (20.81)	6 (18.75)	2 (10.00)	1 (10.00)	48 (16.16)	47 (27.17)
Factor VIIa	36 (7.66)	32 (7.66)	3 (9.38)	1 (5.00)	0 (0)	26 (8.75)	10 (5.78)
Fresh frozen plasma	23 (4.89)	21 (5.02)	2 (6.25)	0 (0)	0 (0)	17 (5.72)	6 (3.47)
Tranexamic acid	15 (3.19)	12 (2.87)	2 (6.25)	1 (5.00)	0 (0)	9 (3.03)	6 (3.47)
Surgical procedures used to correct bleeding	88 (18.72)	78 (18.66)	7 (21.88)	3 (15.00)	1 (10.00)	63 (21.21)	25 (14.45)
**Death**							

At the end of the 1st major bleeding event	20 (4.26)	9 (2.15)	9 (28.13)	2 (10.00)	7 (70.00)	12 (4.04)	8 (4.62)

Type of FXai and the FXai indication.

	Specific FXai			FXai indication		
	Apixaban (N=237)	Rivaroxaban (N=114)	Edoxaban (N=119)	VTE (N=83)	AF (N=367)	Non-mechanical cardiac-valve replacement (N=20)
**Biodemographic data**						
Age at index date (years)	78.73 (9.62)	76.31 (9.07)	77.88 (10.34)	78.01 (10.34)	77.9 (9.46)	78.05 (11.84)
<45 years	0 (0)	0 (0)	0 (0)	0 (0)	0 (0)	0 (0)
45-64 years	20 (8.44)	10 (8.77)	10 (8.4)	9 (10.84)	28 (7.63)	3 (15.00)
65-74 years	65 (27.43)	38 (33.33)	37 (31.09)	22 (26.51)	114 (31.06)	4 (20.00)
75-84 years	93 (39.24)	47 (41.23)	43 (36.13)	29 (34.94)	148 (40.33)	6 (30.00)
≥85 years	59 (24.89)	19 (16.67)	29 (24.37)	23 (27.71)	77 (20.98)	7 (35.00)
Sex, female	143 (60.34)	75 (65.79)	69 (57.98)	42 (50.6)	233 (63.49)	12 (60.00)
BMI	28.13 (5.69)	27.1 (4.98)	26.59 (4.35)	28.4 (4.79)	27.32 (5.38)	26.85 (4.15)
Alcohol use	1 (0.42)	2 (1.75)	1 (0.84)	0 (0)	4 (1.09)	0 (0)
Updated Charlson comorbidity index	7.12 (2.01)	7.65 (2.29)	7.03 (2.17)	6.89 (2.01)	7.33 (2.17)	6.65 (1.69)
**Cardiovascular risk factors**						
Hypertension	191 (80.59)	92 (80.7)	87 (73.11)	67 (80.72)	287 (78.2)	16 (80.00)
Hypercholesterolemia	113 (47.68)	61 (53.51)	49 (41.18)	47 (56.63)	166 (45.23)	10 (50.00)
Type 1 diabetes	7 (2.95)	3 (2.63)	3 (2.52)	0 (0)	13 (3.54)	0 (0)
Type 2 diabetes	69 (29.11)	51 (44.74)	39 (32.77)	20 (24.1)	133 (36.24)	6 (30.00)
Smoking	3 (1.27)	0 (0)	1 (0.84)	0 (0)	4 (1.09)	0 (0)
**Vascular disease**						
Heart failure	54 (22.78)	36 (31.58)	22 (18.49)	26 (31.33)	77 (20.98)	9 (45.00)
Chronic kidney disease	36 (15.19)	25 (21.93)	20 (16.81)	14 (16.87)	62 (16.89)	5 (25.00)
Coronary artery disease	40 (16.88)	15 (13.16)	17 (14.29)	9 (10.84)	59 (16.08)	4 (20.00)
Myocardial infarction	20 (8.44)	10 (8.77)	8 (6.72)	4 (4.82)	33 (8.99)	1 (5.00)
Peripheral artery disease	25 (10.55)	13 (11.4)	7 (5.88)	3 (3.61)	37 (10.08)	5 (25.00)
Cerebrovascular disease	20 (8.44)	11 (9.65)	7 (5.88)	6 (7.23)	30 (8.17)	2 (10.00)
**Conditions that increase the risk of bleeding**						
Cancer	26 (10.97)	13 (11.4)	14 (11.76)	10 (12.05)	40 (10.9)	3 (15.00)
Anemia	48 (20.25)	20 (17.54)	25 (21.01)	16 (19.28)	72 (19.62)	5 (25.00)
Surgical procedures within 30 days prior to bleeding event	36 (15.19)	17 (14.91)	21 (17.65)	9 (10.84)	64 (17.44)	1 (5.00)
Major bleeding history >60 days prior to FXa start	30 (12.66)	12 (10.53)	8 (6.72)	10 (12.05)	38 (10.35)	2 (10.00)
Trauma within 30 days prior to bleeding event	14 (5.91)	8 (7.02)	12 (10.08)	10 (12.05)	23 (6.27)	1 (5.00)
Liver disease	14 (5.91)	6 (5.26)	6 (5.04)	6 (7.23)	20 (5.45)	0 (0)
Peptic ulcer disease	12 (5.06)	10 (8.77)	6 (5.04)	7 (8.43)	21 (5.72)	0 (0)
**FXai use during the follow-up**						
Year of first FXai use						
2013	3 (1.27)	3 (2.63)	0 (0)	1 (1.2)	5 (1.36)	0 (0)
2014	10 (4.22)	6 (5.26)	0 (0)	4 (4.82)	12 (3.27)	0 (0)
2015	12 (5.06)	6 (5.26)	0 (0)	3 (3.61)	14 (3.81)	1 (5.00)
2016	10 (4.22)	2 (1.75)	0 (0)	3 (3.61)	7 (1.91)	2 (10.00)
2017	14 (5.91)	13 (11.4)	6 (5.04)	7 (8.43)	25 (6.81)	1 (5.00)
2018	22 (9.28)	13 (11.4)	18 (15.13)	10 (12.05)	41 (11.17)	2 (10.00)

2019	19 (8.02)	13 (11.4)	18 (15.13)	2 (2.41)	44 (11.99)	4 (20.00)
2020	32 (13.5)	11 (9.65)	20 (16.81)	12 (14.46)	50 (13.62)	1 (5.00)
2021	49 (20.68)	22 (19.3)	29 (24.37)	21 (25.3)	75 (20.44)	4 (20.00)
2022	66 (27.85)	25 (21.93)	28 (23.53)	20 (24.1)	94 (25.61)	5 (25.00)
FXai indications						
VTE	38 (16.03)	21 (18.42)	24 (20.17)	83 (100)	0 (0)	0 (0)
AF	185 (78.06)	89 (78.07)	93 (78.15)	0 (0)	367 (100)	0 (0)
Non-mechanical cardiac-valve replacement	14 (5.91)	4 (3.51)	2 (1.68)	0 (0)	0 (0)	20 (100.00)
**Concomitant treatments (within 120 days prior to index)**						
Gastroprotective agents	187 (78.90)	85 (74.56)	87 (73.11)	68 (81.93)	279 (76.02)	12 (60.00)
Antihypertensive therapies	191 (80.59)	92 (80.7)	87 (73.11)	67 (80.72)	287 (78.2)	16 (80.00)
Lipid lowering therapies	152 (64.14)	79 (69.3)	66 (55.46)	56 (67.47)	225 (61.31)	16 (80.00)
Antidiabetic drugs	75 (31.65)	54 (47.37)	42 (35.29)	20 (24.1)	145 (39.51)	6 (30.00)
NSAIDs	79 (33.33)	43 (37.72)	36 (30.25)	34 (40.96)	118 (32.15)	6 (30.00)
Antiplatelet drugs	30 (12.66)	20 (17.54)	20 (16.81)	10 (12.05)	57 (15.53)	3 (15.00)
Antidepressants	35 (14.77)	10 (8.77)	17 (14.29)	12 (14.46)	46 (12.53)	4 (20.00)
Anticancer drugs*	20 (8.44)	10 (8.77)	7 (5.88)	8 (9.64)	28 (7.63)	1 (5.00)
**Biochemical parameters**						
Hemoglobin, g/dL	11.68 (1.56)	11.68 (1.57)	11.93 (1.5)	11.55 (1.48)	11.78 (1.56)	11.98 (1.48)
HbA1c,	7.54 (1.12)	7.12 (1.24)	7.58 (1.2)	7.52 (1.24)	7.43 (1.17)	7.46 (1.15)
Platelet count, ×103/μL	223.99 (109.98)	239.5 (116.22)	251.4 (111.07)	238.46 (102.03)	234.95 (115.13)	214.36 (98.67)
eGFR, mL/min/1.73 m^2	93.18 (11.66)	94.18 (12.28)	93.78 (14.06)	93.32 (10.4)	93.45 (13.11)	96.84 (5.36)
Creatinine clearance, mL/min	47.63 (23.41)	49.15 (22.68)	49.32 (18.82)	46.96 (22.99)	48.91 (21.94)	45.57 (22.44)
**Actions taken to control bleeding**						
Prothrombin complex concentrates	150 (63.29)	70 (61.4)	77 (64.71)	53 (63.86)	230 (62.67)	14 (70.00)
Transfusion of blood products	51 (21.52)	21 (18.42)	23 (19.33)	19 (22.89)	71 (19.35)	5 (25.00)
Other agents (Factor VIIa)	20 (8.44)	8 (7.02)	8 (6.72)	6 (7.23)	29 (7.9)	1 (5.00)
Fresh frozen plasma	8 (3.38)	4 (3.51)	11 (9.24)	4 (4.82)	18 (4.9)	1 (5.00)
Tranexamic acid	5 (2.11)	6 (5.26)	4 (3.36)	2 (2.41)	12 (3.27)	1 (5.00)
Surgical procedures used to correct bleeding	38 (16.03)	21 (18.42)	29 (24.37)	19 (22.89)	66 (17.98)	3 (15.00)
**Death**						
At the end of the 1st major bleeding event	15 (6.33)	4 (3.51)	1 (0.84)	3 (3.61)	14 (3.81)	3 (15.00)

Consider two tablets or capsules per day for apixaban and one per day for rivaroxaban and edoxaban; *within 180 days prior to the index date. Quantitative variables are presented as mean and (Standard deviation): qualitative variables are presented as absolute and relative (%) frequencies.

AF: atrial fibrillation; BMI: body mass index; eGFR: estimated glomerular filtration rate; FXai; Factor Xa inhibitors; NSAIDs: non-steroidal anti-inflammatory drugs; SD: Standard deviation; VTE: venous thromboembolism.

Supplementary Table S3: CHA_2DS_2-VASc and HAS-BLED scores on the day prior to index date.*

	Overall N= 470	GIB N= 418	ICH N= 32	Trauma bleeding N= 10	Other MB N= 20	VTE N = 83	AF N = 367	Non-mechanical cardiac-valve replacement N = 20
**CHA_2D_2-VASc**								
Baseline mean (SD)	3.44 (1.51)	3.43 (1.53)	3.5 (1.14)	3 (1.49)	3.6 (1.64)	3.34 (1.17)	3.44 (1.56)	3.85 (1.79)
3 months from index date mean (SD)	4.84 (1.79)	4.82 (1.8)	5.05 (1.73)	4 (1.73)	5.06 (1.59)	4.74 (1.52)	4.86 (1.84)	4.94 (1.89)
**HAS-BLED**								
Baseline mean (SD)	1.67 (1.1)	1.69 (1.1)	1.41 (1.16)	1.10 (0.99)	1.60 (0.88)	1.87 (1.16)	1.65 (1.08)	1.30 (1.03)
3 months from index date mean (SD)	3.05 (1.34)	3.05 (1.34)	2.82 (1.33)	3.33 (1.15)	3.28 (1.32)	3.31 (1.33)	2.98 (1.35)	3.18 (1.07)

*Index date: day of the 1st recording with FXai. Quantitative variables are presented as mean and (Standard deviation);

AF: atrial fibrillation; FXai; Factor Xa inhibitors; MB: major bleeding; GIB: gastrointestinal bleeding; ICH: intracranial hemorrhage; SD: Standard deviation; VTE: venous thromboembolism.

Supplementary Table S4. Cumulative clinical outcomes in the overall study population and according to the use of prothrombin complex concentrates, the type of first major bleeding, the type of FXai and the FXai indication.

Overall study population and according to the use of prothrombin complex concentrates.

Time window[1]	All FXai (N=470)			All FXai with Prothrombin complex concentrates (N= 297)			All FXai Without Prothrombin complex concentrates(N=173)		
**Incidence Rates[2]**									
	n	%	Incidence rates per 100 patient-years (95% CI)[2]	n	%	Incidence rates per 100 patient-years (95% CI)[2]	n	%	Incidence rates per 100 patient-years (95% CI)[2]
**3 months since index date**									
Death from any cause	36	7.66	32.71 (28.47 - 36.95)	22	7.41	31.54 (26.26 - 36.82)	14	8.09	34.74 (27.64 - 41.84)
Within index hospitalization	20	4.26	18.17 (14.68 - 21.66)	12	4.04	17.2 (12.91 - 21.49)	8	4.62	19.85 (13.91 - 25.79)
Cardiovascular death	28	5.96	25.44 (21.5 - 29.38)	17	5.72	24.37 (19.49 - 29.25)	11	6.36	27.29 (20.65 - 33.93)
Within index hospitalization	20	4.26	18.17 (14.68 - 21.66)	12	4.04	17.20 (12.91 - 21.49)	8	4.62	19.85 (13.91 - 25.79)
AMI	25	5.32	23.22 (19.4 - 27.04)	15	5.05	22.03 (17.31 - 26.75)	10	5.78	25.28 (18.8 - 31.76)
Within index hospitalization	5	1.06	4.64 (2.74 - 6.54)	2	0.67	2.94 (1.02 - 4.86)	3	1.73	7.59 (3.64 - 11.54)
Ischemic stroke	22	4.68	20.42 (16.78 - 24.06)	14	4.71	20.58 (15.98 - 25.18)	8	4.62	20.14 (14.16 - 26.12)
Within index hospitalization	1	0.21	0.93 (0.06 - 1.8)	1	0.34	1.47 (0.10 - 2.84)	0	0.00	0 (0 - 0)
Acute kidney failure	11	2.34	10.07 (7.35 - 12.79)	7	2.36	10.09 (6.66 - 13.52)	4	2.31	10.05 (5.57 - 14.53)
Within index hospitalization	1	0.21	0.92 (0.06 - 1.78)	0	0.00	0 (0 - 0)	1	0.58	2.51 (0.18 - 4.84)
Acute liver failure	1	0.21	0.91 (0.05 - 1.77)	1	0.34	1.43 (0.08 - 2.78)	0	0.00	0 (0 - 0)
Within index hospitalization	0	0.00	0 (0 - 0)	0	0.00	0 (0 - 0)	0	0.00	0 (0 - 0)
**6 months since index date**									
Death from any cause	71	15.11	32.82 (28.57 - 37.07)	45	15.15	32.86 6 (27.52 - 38.2)	26	15.0 3	32.75 (25.76 - 39.74)
Cardiovascular death	42	8.94	19.41 (15.83 - 22.99)	26	8.75	18.98 (14.52 - 23.44)	16	9.25	20.16 (14.18 - 26.14)
AMI	34	7.23	16.20 (12.87 - 19.53)	21	7.07	15.88 (11.72 - 20.04)	13	7.51	16.75 (11.19 - 22.31)
Ischemic stroke	31	6.60	14.82 (11.61 - 18.03)	21	7.07	15.89 (11.73 - 20.05)	10	5.78	12.98 (7.97 - 17.99)
Acute kidney failure	16	3.40	7.54 (5.15 - 9.93)	11	3.70	8.18 (5.06 - 11.3)	5	2.89	6.43 (2.77 - 10.09)
Acute liver failure	3	0.64	1.39 (0.33 - 2.45)	1	0.34	0.73 (0 - 1.7)	2	1.16	2.52 (0.18 - 4.86)
**12 months since index date**									
Death from any cause	109	23.19	28.21 (24.14 - 32.28)	67	22.56	27.41 (22.34 - 32.48)	42	24.2 8	29.58 (22.78 - 36.38)
Cardiovascular death	64	13.62	16.56 (13.2 - 19.92)	37	12.46	15.14 (11.06 - 19.22)	27	15.6 1	19.01 (13.16 - 24.86)
AMI	42	8.94	11.24 (8.38 - 14.1)	25	8.42	10.61 (7.1 - 14.12)	17	9.83	12.3 (7.41 - 17.19)
Ischemic stroke	37	7.87	9.95 (7.24 - 12.66)	26	8.75	11.10 (7.53 - 14.67)	11	6.36	7.98 (3.94 - 12.02)
Acute kidney failure	26	5.53	6.91 (4.62 - 9.2)	18	6.06	7.56 (4.55 - 10.57)	8	4.62	5.78 (2.3 - 9.26)
Acute liver failure	6	1.28	1.56 (0.44 - 2.68)	2	0.67	0.82 (0 - 1.85)	4	2.31	2.83 (0.36 - 5.3)
**2 years since index date**									
Death from any cause	125	26.60	19.31 (15.74 - 22.88)	79	26.60	19.37 (14.88 - 23.86)	46	26.5 9	19.22 (13.35 - 25.09)

Cardiovascular death	71	15.11	10.97 (8.14 - 13.8)	42	14.14	10.30 (6.84 - 13.76)	29	16.76	12.12 (7.26 - 16.98)
AMI	55	11.70	8.87 (6.3 - 11.44)	33	11.11	8.45 (5.28 - 11.62)	22	12.72	9.57 (5.19 - 13.95)
Ischemic stroke	41	8.72	6.60 (4.36 - 8.84)	29	9.76	7.46 (4.47 - 10.45)	12	6.94	5.15 (1.86 - 8.44)
Acute kidney failure	31	6.60	4.98 (3.01 - 6.95)	21	7.07	5.36 (2.8 - 7.92)	10	5.78	4.34 (1.3 - 7.38)
Acute liver failure	8	1.70	1.24 (0.24 - 2.24)	4	1.35	0.99 (0 - 2.12)	4	2.31	1.68 (0 - 3.6)
**3 years since index date**									
Death from any cause	136	28.94	16.42 (13.07 - 19.77)	88	29.63	16.95 (12.68 - 21.22)	48	27.75	15.53 (10.13 - 20.93)
Cardiovascular death	77	16.38	9.3 (6.67 - 11.93)	46	15.49	8.86 (5.63 - 12.09)	31	17.92	10.03 (5.55 - 14.51)
AMI	58	12.34	7.34 (4.98 - 9.7)	35	11.78	7.07 (4.15 - 9.99)	23	13.29	7.78 (3.79 - 11.77)
Ischemic stroke	43	9.15	5.42 (3.37 - 7.47)	31	10.44	6.31 (3.54 - 9.08)	12	6.94	3.98 (1.07 - 6.89)
Acute kidney failure	33	7.02	4.17 (2.36 - 5.98)	21	7.07	4.23 (1.94 - 6.52)	12	6.94	4.07 (1.13 - 7.01)
Acute liver failure	8	1.70	0.97 (0.08 - 1.86)	4	1.35	0.78 (0 - 1.78)	4	2.31	1.30 (0 - 2.99)
**Event Rates[3]**									
	**Sum of outcomes**	**%**	**Rate per 100 patient-years**	**Sum of outcomes**	**%**	**Rate per 100 patient-years**	**Sum of outcomes**	**%**	**Rate per 100 patient-years**
**3 months since index date**									
AMI	25	5.32	21.57 (17.85 - 25.29)	15	5.05	20.48 (15.89 - 25.07)	10	5.78	23.44 (17.13 - 29.75)
Ischemic stroke	24	4.68	20.71 (17.05 - 24.37)	16	4.71	21.85 (17.15 - 26.55)	8	4.62	18.75 (12.93 - 24.57)
Acute kidney failure	11	2.34	9.49 (6.84 - 12.14)	7	2.36	9.56 (6.22 - 12.9)	4	2.31	9.38 (5.04 - 13.72)
Acute liver failure	1	0.21	0.86 (0.03 - 1.69)	1	0.34	1.37 (0.05 - 2.69)	0	0.00	0 (0 - 0)
**6 months since index date**									
AMI	38	7.23	16.17 (12.84 - 19.5)	23	7.07	15.49 (11.38 - 19.6)	15	7.51	17.33 (11.69 - 22.97)
Ischemic stroke	34	6.60	14.47 (11.29 - 17.65)	24	7.07	16.16 (11.97 - 20.35)	10	5.78	11.55 (6.79 - 16.31)
Acute kidney failure	18	3.40	7.66 (5.26 - 10.06)	12	3.70	8.08 (4.98 - 11.18)	6	2.89	6.93 (3.15 - 10.71)
Acute liver failure	3	0.64	1.28 (0.26 - 2.3)	1	0.34	0.67 (0 - 1.6)	2	1.16	2.31 (0.07 - 4.55)
**12 months since index date**									
AMI	47	8.94	10.47 (7.7 - 13.24)	27	8.42	9.53 (6.19 - 12.87)	20	9.83	12.09 (7.23 - 16.95)
Ischemic stroke	41	7.87	9.14 (6.53 - 11.75)	30	8.75	10.59 (7.09 - 14.09)	11	6.36	6.65 (2.94 - 10.36)
Acute kidney failure	29	5.53	6.46 (4.24 - 8.68)	19	6.06	6.71 (3.86 - 9.56)	10	4.62	6.04 (2.49 - 9.59)
Acute liver failure	7	1.28	1.56 (0.44 - 2.68)	2	0.67	0.71 (0 - 1.66)	5	2.31	3.02 (0.47 - 5.57)
**2 years since index date**									
AMI	63	11.70	7.95 (5.5 - 10.4)	37	11.11	7.39 (4.41 - 10.37)	26	12.72	8.9 (4.66 - 13.14)
Ischemic stroke	45	8.72	5.68 (3.59 - 7.77)	33	9.76	6.59 (3.77 - 9.41)	12	6.94	4.11 (1.15 - 7.07)
Acute kidney failure	35	6.60	4.41 (2.55 - 6.27)	22	7.07	4.39 (2.06 - 6.72)	13	5.78	4.45 (1.38 - 7.52)
Acute liver failure	9	1.70	1.14 (0.18 - 2.1)	4	1.35	0.8 (0 - 1.81)	5	2.31	1.71 (0 - 3.64)
**3 years since index date**									
AMI	67	12.34	6.42 (4.2 - 8.64)	39	11.78	5.9 (3.22 - 8.58)	28	13.29	7.34 (3.45 - 11.23)
Ischemic stroke	47	9.15	4.51 (2.63 - 6.39)	35	10.44	5.29 (2.74 - 7.84)	12	6.94	3.14 (0.54 - 5.74)
Acute kidney failure	37	7.02	3.55 (1.88 - 5.22)	22	7.07	3.33 (1.29 - 5.37)	15	6.94	3.93 (1.03 - 6.83)
Acute liver failure	9	1.70	0.86 (0.03 - 1.69)	4	1.35	0.61 (0 - 1.5)	5	2.31	1.31 (0 - 3)

Type of first major bleeding.

Time window[1]	GIB (N=418)			ICH (N=32)			Trauma bleeding (N=10)			Other MB (N=20)		
Incidence Rates[2]												
	n	%	Incidence rates per 100 patient-years (95% CI)[2]	n	%	Incidence rates per 100 patient-years (95% CI)[2]	n	%	Incidence rates per 100 patient-years (95% CI)[2]	n	%	Incidence rates per 100 patient-years (95% CI)[2]
**3 months since index date**												
Death from any cause	23	5.50	23.00 (18.97 - 27.03)	9	28.13	158.01 (31.6 - 284.4)	7	70.00	922.38 (756.5 - 1088.2)	4	20.00	91.42 (79.15 - 103.69)
Within index hospitalization	9	2.15	9.00 (6.26 - 11.74)	9	28.13	158.01 (31.6 - 284.4)	7	70.00	922.38 (756.5 - 1088.2)	2	10.00	45.71 (23.88 - 67.54)
Cardiovascular death	16	3.83	16.00 (12.49 - 19.51)	9	28.13	158.01 (31.6 - 284.4)	7	70.00	922.38 (756.5 - 1088.2)	3	15.00	68.57 (48.22 - 88.92)
Within index hospitalization	9	2.15	9.00 (6.26 - 11.74)	9	28.13	158.01 (31.6 - 284.4)	7	70.00	922.38 (756.5 - 1088.2)	2	10.00	45.71 (23.88 - 67.54)
AMI	20	4.78	20.42 (16.56 - 24.28)	4	12.50	71.89 (56.31 - 87.47)	0	0.00	0 (0 - 0)	1	5.26	24.08 (4.85 - 43.31)
Within index hospitalization	2	0.48	2.04 (0.68 - 3.4)	3	9.38	53.91 (36.64 - 71.18)	0	0.00	0 (0 - 0)	0	0.00	0 (0 - 0)
Ischemic stroke	21	5.02	21.45 (17.51 - 25.39)	1	3.13	18.24 (4.86 - 31.62)	0	0.00	0 (0 - 0)	0	0.00	0 (0 - 0)
Within index hospitalization	1	0.24	1.02 (0.06 - 1.98)	0	0.00	0 (0 - 0)	0	0.00	0 (0 - 0)	0	0.00	0 (0 - 0)
Acute kidney failure	9	2.15	9.08 (6.33 - 11.83)	2	6.25	35.3 (18.74 - 51.86)	0	0.00	0 (0 - 0)	0	0.00	0 (0 - 0)
Within index hospitalization	1	0.24	1.01 (0.05 - 1.97)	0	0.00	0 (0 - 0)	0	0.00	0 (0 - 0)	0	0.00	0 (0 - 0)
Acute liver failure	1	0.24	1 (0.05 - 1.95)	0	0.00	0 (0 - 0)	0	0.00	0 (0 - 0)	0	0.00	0 (0 - 0)
Within index hospitalization	0	0.00	0 (0 - 0)	0	0.00	0 (0 - 0)	0	0.00	0 (0 - 0)	0	0.00	0 (0 - 0)
**6 months since index date**												
Death from any cause	57	13.64	29 (24.65 - 33.35)	10	31.25	88.27 (77.12 - 99.42)	7	70.00	483.9 (17.42 - 79.36)	4	20.00	47.33 (25.45 - 69.21)
Cardiovascular death	30	7.18	15.26 (11.81 - 18.71)	9	28.13	79.44 (65.44 - 93.44)	7	70.00	483.9 (17.42 - 79.36)	3	15.00	35.49 (14.52 - 56.46)
AMI	29	6.94	15.21 (11.77 - 18.65)	4	12.50	36.55 (19.86 - 53.24)	0	0.00	0 (0 - 0)	1	5.26	12.15 (0 - 26.84)
Ischemic stroke	30	7.18	15.8 (12.3 - 19.3)	1	3.13	9.18 (0 - 19.18)	0	0.00	0 (0 - 0)	0	0.00	0 (0 - 0)
Acute kidney failure	13	3.11	6.73 (4.33 - 9.13)	3	9.38	28.03 (12.47 - 43.59)	0	0.00	0 (0 - 0)	0	0.00	0 (0 - 0)

Acute liver failure	3	0.72	1.53 (0.35 - 2.71)	0	0.00	0 (0 - 0)	0	0.00	0 (0 - 0)	0	0.00	0 (0 - 0)
**12 months since index date**												
Death from any cause	91	21.77	25.86 (21.66 - 30.06)	11	34.38	55.96 (38.76 - 73.16)	7	70.00	286.43 (0.62 - 56.66)	7	35.00	46.91 (25.04 - 68.78)
Cardiovascular death	50	11.96	14.21 (10.86 - 17.56)	9	28.13	45.78 (28.52 - 63.04)	7	70.00	286.43 (0.62 - 56.66)	5	25.00	33.5 (12.81 - 54.19)
AMI	37	8.85	10.88 (7.89 - 13.87)	4	12.50	20.95 (6.85 - 35.05)	0	0.00	0 (0 - 0)	1	5.26	6.8 (0 - 18.12)
Ischemic stroke	36	8.61	10.66 (7.7 - 13.62)	1	3.13	5.2 (0 - 12.89)	0	0.00	0 (0 - 0)	0	0.00	0 (0 - 0)
Acute kidney failure	23	5.50	6.7 (4.3 - 9.1)	3	9.38	16.66 (3.75 - 29.57)	0	0.00	0 (0 - 0)	0	0.00	0 (0 - 0)
Acute liver failure	6	1.44	1.71 (0.47 - 2.95)	0	0.00	0 (0 - 0)	0	0.00	0 (0 - 0)	0	0.00	0 (0 - 0)
**2 years since index date**												
Death from any cause	106	25.36	17.9 (14.22 - 21.58)	12	37.50	38.79 (21.91 - 55.67)	7	70.00	157.52 (0 - 38.33)	7	35.00	29.07 (9.17 - 48.97)
Cardiovascular death	57	13.64	9.62 (6.79 - 12.45)	9	28.13	29.09 (13.35 - 44.83)	7	70.00	157.52 (0 - 38.33)	5	25.00	20.77 (2.99 - 38.55)
AMI	50	11.96	8.83 (6.11 - 11.55)	4	12.50	13.17 (1.45 - 24.89)	0	0.00	0 (0 - 0)	1	5.26	4.19 (0 - 13.2)
Ischemic stroke	39	9.33	6.88 (4.45 - 9.31)	2	6.25	6.62 (0 - 15.23)	0	0.00	0 (0 - 0)	0	0.00	0 (0 - 0)
Acute kidney failure	27	6.46	4.74 (2.7 - 6.78)	4	12.50	14.22 (2.12 - 26.32)	0	0.00	0 (0 - 0)	0	0.00	0 (0 - 0)
Acute liver failure	8	1.91	1.36 (0.25 - 2.47)	0	0.00	0 (0 - 0)	0	0.00	0 (0 - 0)	0	0.00	0 (0 - 0)
**3 years since index date**												
Death from any cause	117	27.99	15.44 (11.98 - 18.9)	12	37.50	30.47 (14.52 - 46.42)	7	70.00	123.97 (0 - 32.82)	7	35.00	22.61 (4.28 - 40.94)
Cardiovascular death	63	15.07	8.31 (5.66 - 10.96)	9	28.13	22.85 (8.3 - 37.4)	7	70.00	123.97 (0 - 32.82)	5	25.00	16.15 (0.02 - 32.28)
AMI	53	12.68	7.35 (4.85 - 9.85)	4	12.50	10.30 (0 - 20.83)	0	0.00	0 (0 - 0)	1	5.26	3.25 (0 - 11.22)
Ischemic stroke	40	9.57	5.52 (3.33 - 7.71)	2	6.25	5.31 (0 - 13.08)	0	0.00	0 (0 - 0)	1	5.00	3.30 (0 - 11.13)
Acute kidney failure	28	6.70	3.87 (2.02 - 5.72)	5	15.63	14.12 (2.05 - 26.19)	0	0.00	0 (0 - 0)	0	0.00	0 (0 - 0)
Acute liver failure	8	1.91	1.06 (0.08 - 2.04)	0	0.00	0 (0 - 0)	0	0.00	0 (0 - 0)	0	0.00	0 (0 - 0)
**Event Rates[3]**												
	**Sum of outcomes**	**%**	**Rate per 100 patient-years**	**Sum of outcomes**	**%**	**Rate per 100 patient-years**	**Sum of outcomes**	**%**	**Rate per 100 patient-years**	**Sum of outcomes**	**%**	**Rate per 100 patient-years**
**3 months since index date**												

AMI	20	4.78	19.4 (15.61 - 23.19)	4	12.50	50.69 (33.37 - 68.01)	0	0.00	0 (0 - 0)	1	5.00	20.28 (2.66 - 37.9)
Ischemic stroke	22	5.02	21.35 (17.42 - 25.28)	2	3.13	25.35 (10.28 - 40.42)	0	0.00	0 (0 - 0)	0	0.00	0 (0 - 0)
Acute kidney failure	9	2.15	8.73 (6.02 - 11.44)	2	6.25	25.35 (10.28 - 40.42)	0	0.00	0 (0 - 0)	0	0.00	0 (0 - 0)
Acute liver failure	1	0.24	0.97 (0.03 - 1.91)	0	0.00	0 (0 - 0)	0	0.00	0 (0 - 0)	0	0.00	0 (0 - 0)
**6 months since index date**												
AMI	32	6.94	15.31 (11.86 - 18.76)	5	12.50	31.37 (15.29 - 47.45)	0	0.00	0 (0 - 0)	1	5.00	9.97 (0 - 23.1)
Ischemic stroke	32	7.18	15.31 (11.86 - 18.76)	2	3.13	12.55 (1.07 - 24.03)	0	0.00	0 (0 - 0)	0	0.00	0 (0 - 0)
Acute kidney failure	15	3.11	7.18 (4.71 - 9.65)	3	9.38	18.82 (5.28 - 32.36)	0	0.00	0 (0 - 0)	0	0.00	0 (0 - 0)
Acute liver failure	3	0.72	1.44 (0.3 - 2.58)	0	0.00	0 (0 - 0)	0	0.00	0 (0 - 0)	0	0.00	0 (0 - 0)
**12 months since index date**												
AMI	41	8.85	10.23 (7.32 - 13.14)	5	12.50	17.34 (4.22 - 30.46)	0	0.00	0 (0 - 0)	1	5.00	5.19 (0 - 14.91)
Ischemic stroke	39	8.61	9.73 (6.89 - 12.57)	2	3.13	6.94 (0 - 15.75)	0	0.00	0 (0 - 0)	0	0.00	0 (0 - 0)
Acute kidney failure	26	5.50	6.49 (4.13 - 8.85)	3	9.38	10.41 (0 - 20.99)	0	0.00	0 (0 - 0)	0	0.00	0 (0 - 0)
Acute liver failure	7	1.44	1.75 (0.49 - 3.01)	0	0.00	0 (0 - 0)	0	0.00	0 (0 - 0)	0	0.00	0 (0 - 0)
**2 years since index date**												
AMI	57	11.96	8.01 (5.41 - 10.61)	5	12.50	10.51 (0 - 21.14)	0	0.00	0 (0 - 0)	1	5.00	2.99 (0 - 10.45)
Ischemic stroke	42	10.05	5.9 (3.64 - 8.16)	3	9.38	6.31 (0 - 14.73)	0	0.00	0 (0 - 0)	0	0.00	0 (0 - 0)
Acute kidney failure	31	7.42	4.35 (2.39 - 6.31)	4	12.50	8.41 (0 - 18.03)	0	0.00	0 (0 - 0)	0	0.00	0 (0 - 0)
Acute liver failure	9	2.15	1.26 (0.19 - 2.33)	0	0.00	0 (0 - 0)	0	0.00	0 (0 - 0)	0	0.00	0 (0 - 0)
**3 years since index date**												
AMI	61	14.59	6.5 (4.14 - 8.86)	5	15.63	8.43 (0 - 18.06)	0	0.00	0 (0 - 0)	1	5.00	2.21 (0 - 8.65)
Ischemic stroke	43	10.29	4.58 (2.58 - 6.58)	3	9.38	5.06 (0 - 12.65)	0	0.00	0 (0 - 0)	1	5.00	2.21 (0 - 8.65)
Acute kidney failure	32	7.66	3.41 (1.67 - 5.15)	5	15.63	8.43 (0 - 18.06)	0	0.00	0 (0 - 0)	0	0.00	0 (0 - 0)
Acute liver failure	9	2.15	0.96 (0.03 - 1.89)	0	0.00	0 (0 - 0)	0	0.00	0 (0 - 0)	0	0.00	0 (0 - 0)

Type of FXai.

	Apixaban (N=237)			Rivaroxaban (N=114)			Edoxaban (N=119)		
Incidence Rates[2]									
	n	%	Incidence rates per 100 patient-years (95% CI)[2]	n	%	Incidence rates per 100 patient-years (95% CI)[2]	n	%	Incidence rates per 100 patient-years (95% CI)[2]
3 months since index date									
Death from any cause	21	8.86	38.58 (32.38 - 44.78)	9	7.89	33.55 (24.88 - 42.22)	6	5.04	20.83 (13.53 - 28.13)
Within index hospitalization	15	6.33	27.56 (21.87 - 33.25)	4	3.51	14.91 (8.37 - 21.45)	1	0.84	3.47 (0.18 - 6.76)
Cardiovascular death	19	8.02	34.91 (28.84 - 40.98)	6	5.26	22.37 (14.72 - 30.02)	3	2.52	10.41 (4.92 - 15.9)
Within index hospitalization	15	6.33	27.56 (21.87 - 33.25)	4	3.51	14.91 (8.37 - 21.45)	1	0.84	3.47 (0.18 - 6.76)
AMI	14	5.91	26.33 (20.72 - 31.94)	6	5.31	22.76 (15.03 - 30.49)	5	4.20	17.79 (10.92 - 24.66)
Within index hospitalization	4	1.69	7.52 (4.16 - 10.88)	1	0.88	3.79 (0.27 - 7.31)	0	0.00	0 (0 - 0)
Ischemic stroke	9	3.80	16.82 (12.06 - 21.58)	8	7.02	30.61 (22.15 - 39.07)	5	4.20	17.79 (10.92 - 24.66)
Within index hospitalization	0	0.00	0 (0 - 0)	1	0.88	3.83 (0.31 - 7.35)	0	0.00	0 (0 - 0)
Acute kidney failure	7	2.95	13.01 (8.73 - 17.29)	2	1.75	7.47 (2.64 - 12.3)	2	1.68	7 (2.42 - 11.58)
Within index hospitalization	1	0.42	1.86 (0.14 - 3.58)	0	0.00	0 (0 - 0)	0	0.00	0 (0 - 0)
Acute liver failure	1	0.42	1.84 (0.13 - 3.55)	0	0.00	0 (0 - 0)	0	0.00	0 (0 - 0)
Within index hospitalization	0	0.00	0 (0 - 0)	0	0.00	0 (0 - 0)	0	0.00	0 (0 - 0)
6 months since index date									
Death from any cause	40	16.88	37.32 (31.16 - 43.48)	17	14.91	32.49 (23.89 - 41.09)	14	11.76	24.64 (16.9 - 32.38)
Cardiovascular death	25	10.55	23.32 (17.94 - 28.7)	11	9.65	21.02 (13.54 - 28.5)	6	5.04	10.56 (5.04 - 16.08)
AMI	17	7.17	16.35 (11.64 - 21.06)	11	9.73	21.86 (14.24 - 29.48)	6	5.04	10.8 (5.22 - 16.38)
Ischemic stroke	14	5.91	13.47 (9.12 - 17.82)	9	7.89	17.73 (10.72 - 24.74)	8	6.72	14.67 (8.31 - 21.03)
Acute kidney failure	10	4.22	9.56 (5.82 - 13.3)	3	2.63	5.81 (1.52 - 10.1)	3	2.52	5.36 (1.31 - 9.41)
Acute liver failure	3	1.27	2.81 (0.71 - 4.91)	0	0.00	0 (0 - 0)	0	0.00	0 (0 - 0)
12 months since index date									
Death from any cause	61	25.74	32.04 (26.1 - 37.98)	24	21.05	25.68 (17.66 - 33.7)	24	20.17	23.39 (15.78 - 31)

Cardiovascular death	36	15.19	18.91 (13.92 - 23.9)	17	14.91	18.19 (11.11 - 25.27)	11	9.24	10.72 (5.16 - 16.28)
AMI	23	9.70	12.54 (8.32 - 16.76)	12	10.62	13.32 (7.05 - 19.59)	7	5.88	6.97 (2.39 - 11.55)
Ischemic stroke	18	7.59	9.8 (6.01 - 13.59)	10	8.77	10.97 (5.23 - 16.71)	9	7.56	9.27 (4.06 - 14.48)
Acute kidney failure	14	5.91	7.61 (4.23 - 10.99)	8	7.02	8.73 (3.55 - 13.91)	4	3.36	3.97 (0.46 - 7.48)
Acute liver failure	4	1.69	2.11 (0.28 - 3.94)	1	0.88	1.07 (0 - 2.96)	1	0.84	0.98 (0 - 2.75)
**2 years since index date**									
Death from any cause	70	29.54	22.19 (16.9 - 27.48)	28	24.56	17.51 (10.53 - 24.49)	27	22.69	15.71 (9.17 - 22.25)
Cardiovascular death	40	16.88	12.68 (8.44 - 16.92)	19	16.67	11.88 (5.94 - 17.82)	12	10.08	6.98 (2.4 - 11.56)
AMI	28	11.81	9.31 (5.61 - 13.01)	16	14.16	10.47 (4.82 - 16.12)	11	9.24	6.6 (2.14 - 11.06)
Ischemic stroke	21	8.86	6.92 (3.69 - 10.15)	10	8.77	6.38 (1.89 - 10.87)	10	8.40	6.19 (1.86 - 10.52)
Acute kidney failure	17	7.17	5.67 (2.73 - 8.61)	9	7.89	5.84 (1.54 - 10.14)	5	4.20	2.97 (0 - 6.02)
Acute liver failure	5	2.11	1.59 (0 - 3.18)	2	1.75	1.26 (0 - 3.31)	1	0.84	0.58 (0 - 1.94)
**3 years since index date**									
Death from any cause	74	31.22	18.45 (13.51 - 23.39)	31	27.19	14.98 (8.43 - 21.53)	31	26.05	14.07 (7.82 - 20.32)
Cardiovascular death	43	18.14	10.72 (6.78 - 14.66)	21	18.42	10.15 (4.61 - 15.69)	13	10.92	5.9 (1.67 - 10.13)
AMI	29	12.24	7.64 (4.26 - 11.02)	16	14.16	8.04 (3.03 - 13.05)	13	10.92	6.13 (1.82 - 10.44)
Ischemic stroke	23	9.70	6.02 (2.99 - 9.05)	10	8.77	4.92 (0.95 - 8.89)	10	8.40	4.81 (0.97 - 8.65)
Acute kidney failure	17	7.17	4.49 (1.85 - 7.13)	10	8.77	5.06 (1.04 - 9.08)	6	5.04	2.8 (0 - 5.76)
Acute liver failure	5	2.11	1.25 (0 - 2.66)	2	1.75	0.98 (0 - 2.79)	1	0.84	0.45 (0 - 1.65)
**Event Rates[3]**									
	**Sum of outcomes**	**%**	**Rate per 100 patient-years**	**Sum of outcomes**	**%**	**Rate per 100 patient-years**	**Sum of outcomes**	**%**	**Rate per 100 patient-years**
**3 months since index date**									
AMI	14	5.91	23.96 (18.53 - 29.39)	6	5.26	21.35 (13.83 - 28.87)	5	4.20	17.04 (10.28 - 23.8)
Ischemic stroke	10	3.80	17.11 (12.32 - 21.9)	8	7.02	28.46 (20.18 - 36.74)	6	4.20	20.45 (13.2 - 27.7)
Acute kidney failure	7	2.95	11.98 (7.85 - 16.11)	2	1.75	7.12 (2.4 - 11.84)	2	1.68	6.82 (2.29 - 11.35)
Acute liver failure	1	0.42	1.71 (0.06 - 3.36)	0	0.00	0 (0 - 0)	0	0.00	0 (0 - 0)
**6 months since index date**									
AMI	20	7.17	16.88 (12.11 - 21.65)	11	9.65	19.32 (12.07 - 26.57)	7	5.04	11.74 (5.96 - 17.52)
Ischemic stroke	15	5.91	12.66 (8.43 - 16.89)	10	7.89	17.56 (10.58 - 24.54)	9	6.72	15.1 (8.67 - 21.53)
Acute kidney failure	11	4.22	9.28 (5.59 - 12.97)	3	2.63	5.27 (1.17 - 9.37)	4	2.52	6.71 (2.21 - 11.21)
Acute liver failure	3	1.27	2.53 (0.53 - 4.53)	0	0.00	0 (0 - 0)	0	0.00	0 (0 - 0)

**12 months since index date**									
AMI	27	9.70	12.03 (7.89 - 16.17)	12	10.53	11.02 (5.27 - 16.77)	8	5.88	6.93 (2.37 - 11.49)
Ischemic stroke	19	7.59	8.46 (4.92 - 12)	12	8.77	11.02 (5.27 - 16.77)	10	7.56	8.67 (3.61 - 13.73)
Acute kidney failure	15	5.91	6.68 (3.5 - 9.86)	9	7.02	8.26 (3.21 - 13.31)	5	3.36	4.33 (0.67 - 7.99)
Acute liver failure	5	1.69	2.23 (0.35 - 4.11)	1	0.88	0.92 (0 - 2.67)	1	0.84	0.87 (0 - 2.54)
**2 years since index date**									
AMI	34	11.81	8.65 (5.07 - 12.23)	16	14.04	8.18 (3.15 - 13.21)	13	9.24	6.37 (1.98 - 10.76)
Ischemic stroke	22	8.86	5.59 (2.67 - 8.51)	12	8.77	6.14 (1.73 - 10.55)	11	8.40	5.39 (1.33 - 9.45)
Acute kidney failure	18	7.17	4.58 (1.92 - 7.24)	10	7.89	5.11 (1.07 - 9.15)	7	4.20	3.43 (0.16 - 6.7)
Acute liver failure	6	2.11	1.53 (0 - 3.09)	2	1.75	1.02 (0 - 2.86)	1	0.84	0.49 (0 - 1.74)
**3 years since index date**									
AMI	36	12.24	7.01 (3.76 - 10.26)	16	14.04	6.09 (1.7 - 10.48)	15	10.92	5.62 (1.48 - 9.76)
Ischemic stroke	24	9.70	4.68 (1.99 - 7.37)	12	8.77	4.57 (0.74 - 8.4)	11	8.40	4.12 (0.55 - 7.69)
Acute kidney failure	18	7.17	3.51 (1.17 - 5.85)	11	8.77	4.19 (0.51 - 7.87)	8	5.04	3 (0 - 6.06)
Acute liver failure	6	2.11	1.17 (0 - 2.54)	2	1.75	0.76 (0 - 2.35)	1	0.84	0.37 (0 - 1.46)

Type of FXai indication.

	Venous thromboembolism (N=83)			Atrial fibrillation (N=367)			Non-mechanical cardiac-valve replacement (N=20)		
**Incidence Rates[2]**									
	n	%	Incidence rates per 100 patient-years (95% CI)[2]	n	%	Incidence rates per 100 patient-years (95% CI)[2]	n	%	Incidence rates per 100 patient-years (95% CI)[2]
**3 months since index date**									
Death from any cause	6	7.23	30.92 (20.98 - 40.86)	27	7.36	31.23 (26.49 - 35.97)	3	15.00	71.43 (51.63 - 91.23)
Within index hospitalization	3	3.61	15.46 (7.68 - 23.24)	14	3.81	16.19 (12.42 - 19.96)	3	15.00	71.43 (51.63 - 91.23)
Cardiovascular death	6	7.23	30.92 (20.98 - 40.86)	19	5.18	21.98 (17.74 - 26.22)	3	15.00	71.43 (51.63 - 91.23)
Within index hospitalization	3	3.61	15.46 (7.68 - 23.24)	14	3.81	16.19 (12.42 - 19.96)	3	15.00	71.43 (51.63 - 91.23)
AMI	3	3.61	15.63 (7.82 - 23.44)	21	5.74	24.9 (20.47 - 29.33)	1	5.00	24.19 (5.42 - 42.96)
Within index hospitalization	1	1.20	5.21 (0.43 - 9.99)	4	1.09	4.74 (2.56 - 6.92)	0	0.00	0 (0 - 0)
Ischemic stroke	2	2.41	10.48 (3.89 - 17.07)	20	5.45	23.68 (19.33 - 28.03)	0	0.00	0 (0 - 0)
Within index hospitalization	0	0.00	0 (0 - 0)	1	0.27	1.18 (0.08 - 2.28)	0	0.00	0 (0 - 0)
Acute kidney failure	2	2.41	10.41 (3.84 - 16.98)	9	2.45	10.49 (7.35 - 13.63)	0	0.00	0 (0 - 0)
Within index hospitalization	0	0.00	0 (0 - 0)	1	0.27	1.17 (0.07 - 2.27)	0	0.00	0 (0 - 0)
Acute liver failure	0	0.00	0 (0 - 0)	1	0.27	1.16 (0.06 - 2.26)	0	0.00	0 (0 - 0)
Within index hospitalization	0	0.00	0 (0 - 0)	0	0.00	0 (0 - 0)	0	0.00	0 (0 - 0)
**6 months since index date**									
Death from any cause	12	14.46	31.25 (21.28 - 41.22)	56	15.26	33.06 (28.25 - 37.87)	3	15.00	35.16 (14.23 - 56.09)
Cardiovascular death	8	9.64	20.83 (12.09 - 29.57)	31	8.45	18.3 (14.34 - 22.26)	3	15.00	35.16 (14.23 - 56.09)
AMI	7	8.43	18.63 (10.25 - 27.01)	25	6.83	15.22 (11.54 - 18.9)	2	10.00	25.03 (6.04 - 44.02)
Ischemic stroke	4	4.82	10.69 (4.04 - 17.34)	27	7.36	16.53 (12.73 - 20.33)	0	0.00	0 (0 - 0)
Acute kidney failure	4	4.82	10.69 (4.04 - 17.34)	12	3.27	7.22 (4.57 - 9.87)	0	0.00	0 (0 - 0)
Acute liver failure	0	0.00	0 (0 - 0)	3	0.82	1.78 (0.43 - 3.13)	0	0.00	0 (0 - 0)
**12 months since index date**									

Death from any cause	17	20.48	24.44 (15.19 - 33.69)	87	23.71	28.91 (24.27 - 33.55)	5	25.00	31.3 (10.98 - 51.62)
Cardiovascular death	13	15.66	18.69 (10.3 - 27.08)	47	12.81	15.62 (11.91 - 19.33)	4	20.00	25.04 (6.05 - 44.03)
AMI	9	10.84	13.46 (6.12 - 20.8)	31	8.47	10.6 (7.45 - 13.75)	2	10.00	13.85 (0 - 28.99)
Ischemic stroke	4	4.82	5.92 (0.84 - 11)	33	8.99	11.44 (8.18 - 14.7)	0	0.00	0 (0 - 0)
Acute kidney failure	6	7.23	9.06 (2.88 - 15.24)	20	5.45	6.8 (4.22 - 9.38)	0	0.00	0 (0 - 0)
Acute liver failure	0	0.00	0 (0 - 0)	6	1.63	2 (0.57 - 3.43)	0	0.00	0 (0 - 0)
**2 years since index date**									
Death from any cause	21	25.30	18.16 (9.87 - 26.45)	97	26.43	19.28 (15.24 - 23.32)	7	35.00	24.58 (5.71 - 43.45)
Cardiovascular death	13	15.66	11.24 (4.44 - 18.04)	52	14.17	10.34 (7.22 - 13.46)	6	30.00	21.07 (3.2 - 38.94)
AMI	11	13.25	9.99 (3.54 - 16.44)	42	11.48	8.66 (5.78 - 11.54)	2	10.00	7.92 (0 - 19.76)
Ischemic stroke	4	4.82	3.57 (0 - 7.56)	37	10.08	7.69 (4.96 - 10.42)	0	0.00	0 (0 - 0)
Acute kidney failure	6	7.23	5.56 (0.63 - 10.49)	24	6.54	4.94 (2.72 - 7.16)	1	5.00	3.51 (0 - 11.58)
Acute liver failure	0	0.00	0 (0 - 0)	8	2.18	1.6 (0.32 - 2.88)	0	0.00	0 (0 - 0)
**3 years since index date**									
Death from any cause	21	25.30	14.47 (6.9 - 22.04)	108	29.43	16.73 (12.91 - 20.55)	7	35.00	18.62 (1.56 - 35.68)
Cardiovascular death	13	15.66	8.96 (2.82 - 15.1)	58	15.80	8.99 (6.06 - 11.92)	6	30.00	15.96 (0 - 32.01)
AMI	13	15.66	9.46 (3.16 - 15.76)	43	11.75	6.94 (4.34 - 9.54)	2	10.00	5.99 (0 - 16.39)
Ischemic stroke	4	4.82	2.85 (0 - 6.43)	39	10.63	6.34 (3.85 - 8.83)	0	0.00	0 (0 - 0)
Acute kidney failure	6	7.23	4.47 (0.02 - 8.92)	26	7.08	4.20 (2.15 - 6.25)	1	5.00	2.66 (0 - 9.71)
Acute liver failure	0	0.00	0 (0 - 0)	8	2.18	1.25 (0.11 - 2.39)	0	0.00	0 (0 - 0)
**Event Rates[3]**									
	**Sum of outcomes**	**%**	**Rate per 100 patient-years**	**Sum of outcomes**	**%**	**Rate per 100 patient-years**	**Sum of outcomes**	**%**	**Rate per 100 patient-years**
**3 months since index date**									
AMI	3	3.61	14.66 (7.05 - 22.27)	21	5.72	23.21 (18.89 - 27.53)	1	5.00	20.28 (2.66 - 37.9)
Ischemic stroke	2	2.41	9.77 (3.38 - 16.16)	22	5.45	24.31 (19.92 - 28.7)	0	0.00	0 (0 - 0)
Acute kidney failure	2	2.41	9.77 (3.38 - 16.16)	9	2.45	9.95 (6.89 - 13.01)	0	0.00	0 (0 - 0)
Acute liver failure	0	0.00	0 (0 - 0)	1	0.27	1.11 (0.04 - 2.18)	0	0.00	0 (0 - 0)
**6 months since index date**									

AMI	8	8.43	19.22 (10.74 - 27.7)	28	6.81	15.27 (11.59 - 18.95)	2	10.00	19.95 (2.44 - 37.46)
Ischemic stroke	4	4.82	9.61 (3.27 - 15.95)	30	7.36	16.36 (12.58 - 20.14)	0	0.00	0 (0 - 0)
Acute kidney failure	5	4.82	12.02 (5.02 - 19.02)	13	3.27	7.09 (4.46 - 9.72)	0	0.00	0 (0 - 0)
Acute liver failure	0	0.00	0 (0 - 0)	3	0.82	1.64 (0.34 - 2.94)	0	0.00	0 (0 - 0)
**12 months since index date**									
AMI	11	10.84	13.73 (6.33 - 21.13)	34	8.45	9.73 (6.7 - 12.76)	2	10.00	10.43 (0 - 23.83)
Ischemic stroke	5	4.82	6.24 (1.04 - 11.44)	36	8.99	10.3 (7.19 - 13.41)	0	0.00	0 (0 - 0)
Acute kidney failure	7	7.23	8.74 (2.66 - 14.82)	22	5.45	6.29 (3.81 - 8.77)	0	0.00	0 (0 - 0)
Acute liver failure	0	0.00	0 (0 - 0)	7	1.63	2 (0.57 - 3.43)	0	0.00	0 (0 - 0)
**2 years since index date**									
AMI	14	13.25	9.93 (3.5 - 16.36)	47	11.44	7.61 (4.9 - 10.32)	2	10.00	5.9 (0 - 16.23)
Ischemic stroke	5	6.02	3.54 (0 - 7.52)	40	10.90	6.47 (3.95 - 8.99)	0	0.00	0 (0 - 0)
Acute kidney failure	7	8.43	4.96 (0.29 - 9.63)	27	7.36	4.37 (2.28 - 6.46)	1	5.00	2.95 (0 - 10.37)
Acute liver failure	0	0.00	0 (0 - 0)	9	2.45	1.46 (0.23 - 2.69)	0	0.00	0 (0 - 0)
**3 years since index date**									
AMI	16	19.28	8.72 (2.65 - 14.79)	49	13.35	6.02 (3.59 - 8.45)	2	10.00	4.42 (0 - 13.43)
Ischemic stroke	5	6.02	2.72 (0 - 6.22)	42	11.44	5.16 (2.9 - 7.42)	0	0.00	0 (0 - 0)
Acute kidney failure	7	8.43	3.81 (0 - 7.93)	29	7.90	3.56 (1.66 - 5.46)	1	5.00	2.21 (0 - 8.65)
Acute liver failure	0	0.00	0 (0 - 0)	9	2.45	1.11 (0.04 - 2.18)	0	0.00	0 (0 - 0)

1. Index date: day of the first major bleeding2. Laboratory/radiology investigations. Qualitative variables are presented as absolute and relative (%) frequencies. Rate (95 CI): per 100 person-years. AMI: acute myocardial infarction; FXai; Factor Xa inhibitors; GIB: gastrointestinal bleeding; GP: general practitioners; HCRU: Health Care Resource Utilization; ICH: intracranial bleeding; MB: major bleeding.

1. Time window: cumulative events from index date (day of the 1st major bleeding)

2. Incidence rates defined as the total number of incident events of interest divided by the total person time at risk

3. Event rate defined as the total number of events, including recurrent events divided by the total person time of follow-up

N: number of patients in the total group or subgroup or with the event

Supplementary Table S5. Cumulative outpatient Visits and Hospitalization for 3 years from index date in the overall study population and according to the type of first major bleeding, the type of FXai and the FXai indication.

Overall study population.

Time window[1]	All FXai (N=470)			
	Patients (n)	Visits (n)	% of patients	Rate, number of visits per 100 patient-years (95 CI)
**Cumulative HCRU**				
**6 months since index date**				
**All-cause HCRU**				
Outpatient Visits[2]	444	4313	94.47	1993.62 (1632.42 - 2354.82)
GPs visits	432	3234	91.91	1494.87 (1172.5 - 1817.24)
Specialist visits	382	1079	81.28	498.75 (453.55 - 543.95)
Investigations[3]	470	1271	100.00	587.50 (542.99 - 632.01)
Hospitalization	470	579	100.00	267.63 (227.6 - 307.66)
**Bleeding-related HCRU**				
Outpatient Visits	444	3953	94.47	1827.22 (1477.85 - 2176.59)
GPs visits	432	2894	91.91	1337.71 (1029.96 - 1645.46)
Specialist visits	382	1059	81.28	489.51 (444.32 - 534.7)
Investigations[3]	470	838	100.00	387.35 (343.31 - 431.39)
Hospitalization	470	486	100.00	224.65 (186.92 - 262.38)
**1 year since index date**				
**All-cause HCRU**				
Outpatient Visits	448	6618	95.32	1712.49 (1371.9 - 2053.08)
GPs visits	447	4945	95.11	1279.58 (977.58 - 1581.58)
Specialist visits	428	1673	91.06	432.91 (388.11 - 477.71)
Investigations[3]	470	1973	100.00	510.54 (465.35 - 555.73)
Hospitalization	470	629	100.00	162.76 (129.39 - 196.13)
**Bleeding-related HCRU**				
Outpatient Visits	448	5866	95.32	1517.9 (1193.5 - 1842.3)
GPs visits	447	4405	95.11	1139.85 (852.54 - 1427.16)
Specialist visits	428	1461	91.06	378.05 (334.21 - 421.89)
Investigations[3]	470	1080	100.00	279.46 (238.89 - 320.03)
Hospitalization	470	505	100.00	130.68 (100.21 - 161.15)
**2 years since index date**				
**All-cause HCRU**				
Outpatient Visits	450	9085	95.74	1403.66 (1089.61 - 1717.71)
GPs visits	450	6878	95.74	1062.67 (784.05 - 1341.29)
Specialist visits	443	2207	94.26	340.99 (298.13 - 383.85)
Investigations[3]	470	2387	100.00	368.8 (325.18 - 412.42)
Hospitalization	470	669	100.00	103.36 (75.84 - 130.88)
**Bleeding-related HCRU**				
Outpatient Visits	450	6814	95.74	1052.79 (775.32 - 1330.26)
GPs visits	450	5021	95.74	775.76 (738.05 - 813.47)
Specialist visits	443	1793	94.26	277.02 (236.56 - 317.48)
Investigations[3]	470	1575	100.00	243.34 (204.55 - 282.13)
Hospitalization	470	517	100.00	79.88 (76.26 - 83.5)
**3 years since index date**				
**All-cause HCRU**				
Outpatient Visits	450	11909	95.74	1437.8 (1120.59 - 1755.01)
GPs visits	450	9160	95.74	1105.91 (822.37 - 1389.45)
Specialist visits	450	2749	95.74	331.89 (289.32 - 374.46)
Investigations[3]	470	2866	100.00	346.02 (303.01 - 389.03)
Hospitalization	470	690	100.00	83.31 (79.94 - 86.68)
**Bleeding-related HCRU**				
Outpatient Visits	450	7444	95.74	898.73 (871.46 - 926)
GPs visits	450	5535	95.74	668.25 (625.68 - 710.82)
Specialist visits	443	1909	94.26	230.48 (192.41 - 268.55)
Investigations3	470	1792	100.00	216.35 (179.12 - 253.58)
Hospitalization	470	530	100.00	63.99 (59.65 - 68.33)

Type of first major bleeding.

	GIB (N=418)				ICH (N=32)				Trauma major bleeding (N=10)				Other MB (N=20)			
**Time window[1]**	**Patients (n)**	**Visits (n)**	**% of patients**	**Rate, number of visits per 100 patient-years (95 CI)**	**Patients (n)**	**Visits (n)**	**% of patients**	**Rate, number of visits per 100 patient-years (95 CI)**	**Patients (n)**	**Visits (n)**	**% of patients**	**Rate, number of visits per 100 patient-years (95 CI)**	**Patients (n)**	**Visits (n)**	**% of patients**	**Rate, number of visits per 100 patient-years (95 CI)**
**Cumulative HCRU**																
**6 months since index date**																
**All-cause HCRU**																
Outpatient Visits	404	3906	96.65	1987.19 (1604.65 - 2369.73)	22	223	68.75	1968.44 (590.78 - 3346.1)	3	35	30.00	2419.51 (0 - 5073.92)	18	184	90.00	2176.99 (368.33 - 3985.65)
GPs visits	393	2926	94.02	1488.61 (1147.37 - 1829.85)	22	172	68.75	1518.26 (274.9 - 2761.62)	3	29	30.00	2004.73 (0 - 4486.15)	17	136	85.00	1609.08 (0 - 3219.48)
Specialist visits	347	980	83.01	498.58 (450.65 - 546.51)	19	51	59.38	450.18 (277.8 - 622.56)	2	6	20.00	414.77 (109.4 - 720.14)	16	48	80.00	567.91 (350.81 - 785.01)
Investigations[3]	418	1141	100.00	580.49 (533.18 - 627.8)	32	77	100.00	679.69 (518.02 - 841.36)	10	23	100.00	1589.96 (0 - 3856.42)	20	53	100.00	627.07 (415.13 - 839.01)
Hospitalization	418	516	100.00	262.52 (220.34 - 304.7)	32	42	100.00	370.74 (203.39 - 538.09)	10	10	100.00	691.29 (404.96 - 977.62)	20	21	100.00	248.46 (59.08 - 437.84)
**Bleeding-related HCRU**																
Outpatient Visits	404	3590	96.65	1826.42 (1456.02 - 2196.82)	22	199	68.75	1756.59 (438.12 - 3075.06)	3	32	30.00	2212.12 (0 - 4784.71)	18	164	90.00	1940.36 (207.19 - 3673.53)
GPs visits	393	2626	94.02	1335.99 (1009.83 - 1662.15)	22	150	68.75	1324.06 (149.72 - 2498.4)	3	26	30.00	1797.35 (0 - 4177.2)	17	118	85.00	1396.11 (0 - 2915.08)
Specialist visits	347	964	83.01	490.44 (442.52 - 538.36)	19	49	59.38	432.53 (260.87 - 604.19)	2	6	20.00	414.77 (109.4 - 720.14)	16	46	80.00	544.25 (325.98 - 762.52)
Investigations[3]	418	760	100.00	386.65 (339.96 - 433.34)	32	46	100.00	406.05 (235.89 - 576.21)	10	13	100.00	898.67 (711.63 - 1085.71)	20	32	100.00	378.61 (166.03 - 591.19)
Hospitalization	418	434	100.00	220.8 (181.04 - 260.56)	32	32	100.00	282.47 (126.48 - 438.46)	10	10	100.00	691.29 (404.96 - 977.62)	20	20	100.00	236.63 (50.36 - 422.9)
**1 year since index date**																
**All-cause HCRU**																
Outpatient Visits	407	6018	97.37	1710.27 (1349.3 - 2071.24)	23	325	71.88	1653.31 (366.2 - 2940.42)	3	54	30.00	2209.64 (0 - 4781.19)	18	275	90.00	1842.76 (143.55 - 3541.97)
GPs visits	406	4493	97.13	1276.88 (956.93 - 1596.83)	23	247	71.88	1256.52 (108.08 - 2404.96)	3	45	30.00	1841.37 (0 - 4243.71)	18	205	90.00	1373.69 (0 - 2882.37)
Specialist visits	390	1525	93.30	433.39 (385.88 - 480.9)	20	78	62.50	396.79 (227.28 - 566.3)	2	9	20.00	368.27 (69.32 - 667.22)	18	70	90.00	469.07 (250.36 - 687.78)
Investigations[3]	418	1776	100.00	504.73 (456.8 - 552.66)	32	119	100.00	605.37 (436.02 - 774.72)	10	40	100.00	1636.77 (0 - 3929.94)	20	78	100.00	522.67 (303.76 - 741.58)

Hospitalization	418	566	100.00	160.85 (125.63 - 196.07)	32	42	100.00	213.66 (71.64 - 355.68)	10	10	100.00	409.19 (104.44 - 713.94)	20	21	100.00	140.72 (0 - 293.12)
**Bleeding-related HCRU**																
Outpatient Visits	407	5330	97.37	1514.75 (1171.06 - 1858.44)	23	282	71.88	1434.56 (220.01 - 2649.11)	3	42	30.00	1718.61 (0 - 4056.89)	18	254	90.00	1702.04 (54.97 - 3349.11)
GPs visits	406	4004	97.13	1137.91 (833.48 - 1442.34)	23	215	71.88	1093.73 (12.34 - 2175.12)	3	34	30.00	1391.26 (0 - 3536.27)	18	186	90.00	1246.37 (0 - 2694)
Specialist visits	390	1326	93.30	376.84 (330.38 - 423.3)	20	67	62.50	340.84 (176.61 - 505.07)	2	8	20.00	327.35 (36.51 - 618.19)	18	68	90.00	455.66 (237.39 - 673.93)
Investigations[3]	418	982	100.00	279.08 (236.08 - 322.08)	32	56	100.00	284.88 (128.49 - 441.27)	10	15	100.00	613.79 (312.02 - 915.56)	20	42	100.00	281.44 (84.35 - 478.53)
Hospitalization	418	453	100.00	128.74 (96.63 - 160.85)	32	32	100.00	162.79 (34.88 - 290.7)	10	10	100.00	409.19 (104.44 - 713.94)	20	20	100.00	134.02 (0 - 283.33)
**2 years since index date**																
**All-cause HCRU**																
Outpatient Visits	409	8289	97.85	1399.64 (1067.03 - 1732.25)	23	414	71.88	1338.32 (158.65 - 2517.99)	3	61	30.00	1372.69 (0 - 3505.64)	18	382	90.00	1586.6 (0 - 3187.86)
GPs visits	409	6272	97.85	1059.06 (764.06 - 1354.06)	23	313	71.88	1011.82 (0 - 2056.71)	3	50	30.00	1125.15 (0 - 3083.73)	18	293	90.00	1216.94 (0 - 2649.78)
Specialist visits	404	2017	96.65	340.58 (295.15 - 386.01)	21	101	65.63	326.5 (164.02 - 488.98)	3	11	30.00	247.53 (0 - 515.02)	18	89	90.00	369.65 (158.09 - 581.21)
Investigations[3]	418	2158	100.00	364.39 (318.25 - 410.53)	32	138	100.00	446.11 (273.88 - 618.34)	10	40	100.00	900.12 (714.28 - 1085.96)	20	91	100.00	377.96 (165.45 - 590.47)
Hospitalization	418	604	100.00	101.99 (72.98 - 131)	32	44	100.00	142.24 (21.22 - 263.26)	10	10	100.00	225.03 (0 - 483.86)	20	21	100.00	87.22 (72.59 - 101.85)
**Bleeding-related HCRU**																
Outpatient Visits	409	6219	97.85	1050.11 (756.21 - 1344.01)	23	309	71.88	998.89 (987.35 - 1010.43)	3	47	30.00	1057.64 (0 - 2963.76)	18	286	90.00	1187.87 (0 - 2605.84)
GPs visits	409	4581	97.85	773.52 (733.39 - 813.65)	23	228	71.88	737.05 (584.52 - 889.58)	3	37	30.00	832.61 (601.22 - 1064)	18	212	90.00	880.52 (738.37 - 1022.67)
Specialist visits	404	1638	96.65	276.58 (233.7 - 319.46)	21	81	65.63	261.85 (109.52 - 414.18)	3	10	30.00	225.03 (0 - 483.86)	18	74	90.00	307.35 (105.13 - 509.57)
Investigations[3]	418	1443	100.00	243.66 (202.51 - 284.81)	32	74	100.00	239.22 (91.41 - 387.03)	10	18	100.00	405.06 (100.79 - 709.33)	20	58	100.00	240.9 (53.48 - 428.32)
Hospitalization	418	465	100.00	78.52 (74.58 - 82.46)	32	32	100.00	103.45 (0 - 208.97)	10	10	100.00	225.03 (0 - 483.86)	20	20	100.00	83.07 (66.63 - 99.51)
**3 years since index date**																
**All-cause HCRU**																
Outpatient Visits	409	10915	97.85	1440.11 (1103.52 - 1776.7)	23	543	71.88	1378.65 (184.12 - 2573.18)	3	70	30.00	1239.69 (0 - 3282.24)	18	451	90.00	1456.51 (0 - 3002.53)
GPs visits	409	8405	97.85	1108.94 (807.92 - 1409.96)	23	416	71.88	1056.2 (0 - 2121.12)	3	56	30.00	991.75 (935.69 - 1047.81)	18	339	90.00	1094.81 (0 - 2463.27)

Specialist visits	409	2510	97.85	331.17 (286.05 - 376.29)	23	127	71.88	322.45 (160.5 - 484.4)	3	14	30.00	247.94 (0 - 515.58)	18	112	90.00	361.71 (151.12 - 572.3)
Investigations[3]	418	2600	100.00	343.04 (297.53 - 388.55)	32	157	100.00	398.62 (228.98 - 568.26)	10	40	100.00	708.39 (426.69 - 990.09)	20	109	100.00	352.02 (142.7 - 561.34)
Hospitalization	418	623	100.00	82.2 (78.53 - 85.87)	32	45	100.00	114.25 (4.03 - 224.47)	10	10	100.00	177.1 (0 - 413.71)	20	22	100.00	71.05 (51.17 - 90.93)
**Bleeding-related HCRU**																
Outpatient Visits	409	6795	97.85	896.52 (867.32 - 925.72)	23	331	71.88	840.39 (713.49 - 967.29)	3	50	30.00	885.49 (688.13 - 1082.85)	18	318	90.00	1026.99 (0 - 2357.42)
GPs visits	409	5052	97.85	666.55 (621.35 - 711.75)	23	245	71.88	622.04 (454.04 - 790.04)	3	40	30.00	708.39 (426.69 - 990.09)	18	238	90.00	768.63 (583.81 - 953.45)
Specialist visits	404	1743	97.85	229.97 (189.63 - 270.31)	21	86	65.63	218.35 (75.21 - 361.49)	3	10	30.00	177.1 (0 - 413.71)	18	80	90.00	258.36 (66.52 - 450.2)
Investigations3	418	1643	100.00	216.77 (177.27 - 256.27)	32	82	100.00	208.19 (67.51 - 348.87)	10	19	100.00	336.49 (43.63 - 629.35)	20	67	100.00	216.38 (35.91 - 396.85)
Hospitalization	418	478	100.00	63.07 (58.44 - 67.7)	32	32	100.00	81.25 (67.73 - 94.77)	10	10	100.00	177.1 (0 - 413.71)	20	20	100.00	64.59 (43.63 - 85.55)

Type of FXai.

	Apixaban (N=237)				Rivaroxaban (N=114)				Edoxaban (N=119)			
	Patients (n)	Visits (n)	% of patients	Rate, number of visits per 100 patient-years (95 CI)	Patients (n)	Visits (n)	% of patients	Rate, number of visits per 100 patient-years (95 CI)	Patients (n)	Visits (n)	% of patients	Rate, number of visits per 100 patient-years (95 CI)
**Cumulative HCRU**												
**6 months since index date**												
**All-cause HCRU**												
Outpatient Visits	217	2136	91.56	1992.74 (1484.17 - 2501.31)	110	1073	96.49	2050.71 (1309.54 - 2791.88)	117	1104	98.32	1942.72 (1231.86 - 2653.58)
GPs visits	213	1604	89.87	1496.42 (1042.26 - 1950.58)	106	810	92.98	1548.07 (884.06 - 2212.08)	113	820	94.96	1442.97 (811.62 - 2074.32)
Specialist visits	186	532	78.48	496.32 (432.66 - 559.98)	94	263	82.46	502.64 (410.86 - 594.42)	102	284	85.71	499.76 (409.92 - 589.6)
Investigations[3]	237	617	100.00	575.62 (512.69 - 638.55)	114	315	100.00	602.03 (512.18 - 691.88)	119	339	100.00	596.54 (508.39 - 684.69)
Hospitalization	237	295	100.00	275.21 (218.35 - 332.07)	114	141	100.00	269.48 (188.03 - 350.93)	119	143	100.00	251.64 (173.67 - 329.61)
**Bleeding-related HCRU**												
Outpatient Visits	217	1952	91.56	1821.08 (1329.73 - 2312.43)	110	986	96.49	1884.44 (1166.56 - 2602.32)	117	1015	98.32	1786.11 (1097.92 - 2474.3)
GPs visits	213	1430	89.87	1334.09 (901.2 - 1766.98)	106	729	92.98	1393.26 (757.58 - 2028.94)	113	735	94.96	1293.39 (690.45 - 1896.33)
Specialist visits	186	522	78.48	486.99 (423.35 - 550.63)	94	257	82.46	491.18 (399.41 - 582.95)	102	280	85.71	492.72 (402.89 - 582.55)
Investigations[3]	237	402	100.00	375.04 (313.4 - 436.68)	114	204	100.00	389.88 (300.35 - 479.41)	119	232	100.00	408.25 (319.94 - 496.56)
Hospitalization	237	246	100.00	229.5 (175.96 - 283.04)	114	117	100.00	223.61 (147.12 - 300.1)	119	123	100.00	216.44 (142.45 - 290.43)
**1 year since index date**												
Outpatient Visits	220	3291	92.83	1728.47 (1247.07 - 2209.87)	110	1623	96.49	1736.77 (1041.35 - 2432.19)	118	1704	99.16	1660.73 (992.08 - 2329.38)
GPs visits	219	2451	92.41	1287.29 (860.91 - 1713.67)	110	1213	96.49	1298.03 (681.07 - 1914.99)	118	1281	99.16	1248.47 (654.57 - 1842.37)
Specialist visits	213	840	89.87	441.18 (377.96 - 504.4)	104	410	91.23	438.74 (347.65 - 529.83)	111	423	93.28	412.26 (323.82 - 500.7)

Investigations[3]	237	981	100.00	515.23 (451.6 - 578.86)	114	492	100.00	526.49 (434.83 - 618.15)	119	500	100.00	487.3 (397.49 - 577.11)
Hospitalization	237	320	100.00	168.07 (120.46 - 215.68)	114	155	100.00	165.87 (97.59 - 234.15)	119	154	100.00	150.09 (85.92 - 214.26)
**Bleeding-related HCRU**												
Outpatient Visits	220	2918	92.83	1532.56 (1073.93 - 1991.19)	110	1511	96.49	1616.92 (941.07 - 2292.77)	118	1437	99.16	1400.51 (776.97 - 2024.05)
GPs visits	219	2196	92.41	1153.36 (746.68 - 1560.04)	110	1158	96.49	1239.17 (634.33 - 1844.01)	118	1051	99.16	1024.31 (479.52 - 1569.1)
Specialist visits	213	722	89.87	379.2 (317.43 - 440.97)	104	353	91.23	377.74 (288.74 - 466.74)	111	386	93.28	376.2 (289.16 - 463.24)
Investigations[3]	237	513	100.00	269.43 (212.94 - 325.92)	114	265	100.00	283.58 (200.84 - 366.32)	119	302	100.00	294.33 (212.45 - 376.21)
Hospitalization	237	254	100.00	133.4 (90.11 - 176.69)	114	121	100.00	129.48 (67.85 - 191.11)	119	130	100.00	126.7 (66.93 - 186.47)
**2 years since index date**												
**All-cause HCRU**												
Outpatient Visits	222	4502	93.67	1427.16 (981.83 - 1872.49)	110	2215	96.49	1385.09 (750.98 - 2019.2)	118	2368	99.16	1377.82 (758.54 - 1997.1)
GPs visits	222	3396	93.67	1076.55 (681.94 - 1471.16)	110	1683	96.49	1052.42 (489.11 - 1615.73)	118	1799	99.16	1046.75 (496.71 - 1596.79)
Specialist visits	218	1106	91.98	350.61 (289.86 - 411.36)	109	532	95.61	332.67 (246.18 - 419.16)	116	569	97.48	331.07 (246.52 - 415.62)
Investigations[3]	237	1172	100.00	371.53 (310.01 - 433.05)	114	600	100.00	375.19 (286.31 - 464.07)	119	615	100.00	357.84 (271.71 - 443.97)
Hospitalization	237	337	100.00	106.83 (67.5 - 146.16)	114	168	100.00	105.05 (48.76 - 161.34)	119	164	100.00	95.42 (91.66 - 99.18)
**Bleeding-related HCRU**												
Outpatient Visits	222	3356	93.67	1063.87 (671.31 - 1456.43)	110	1691	96.49	1057.42 (492.93 - 1621.91)	118	1767	99.16	1028.13 (482.44 - 1573.82)
GPs visits	222	2462	93.67	780.47 (727.77 - 833.17)	110	1256	96.49	785.4 (710.04 - 860.76)	118	1303	99.16	758.15 (681.21 - 835.09)
Specialist visits	218	894	91.98	283.4 (226.03 - 340.77)	109	435	95.61	272.01 (190.32 - 353.7)	116	464	97.48	269.98 (190.21 - 349.75)
Investigations[3]	237	728	100.00	230.78 (177.14 - 284.42)	114	397	100.00	248.25 (168.95 - 327.55)	119	450	100.00	261.83 (182.84 - 340.82)
Hospitalization	237	257	100.00	81.47 (76.52 - 86.42)	114	128	100.00	80.04 (72.7 - 87.38)	119	132	100.00	76.8 (69.22 - 84.38)
**3 years since index date**												
**All-cause HCRU**												
Outpatient Visits	222	5882	93.67	1466.74 (1016.32 - 1917.16)	110	2924	96.49	1413.2 (773.73 - 2052.67)	118	3103	99.16	1408.23 (783.26 - 2033.2)

GPs visits	222	4519	93.67	1126.86 (724.28 - 1529.44)	110	2243	96.49	1084.06 (513.35 - 1654.77)	118	2398	99.16	1088.28 (528.74 - 1647.82)
Specialist visits	222	1363	93.67	339.88 (279.57 - 400.19)	110	681	96.49	329.13 (242.87 - 415.39)	118	705	99.16	319.95 (236.14 - 403.76)
Investigations[3]	237	1422	100.00	354.59 (293.68 - 415.5)	114	705	100.00	340.73 (253.73 - 427.73)	119	739	100.00	335.38 (250.55 - 420.21)
Hospitalization	237	344	100.00	85.78 (81.33 - 90.23)	114	174	100.00	84.1 (77.39 - 90.81)	119	172	100.00	78.06 (70.62 - 85.5)
**Bleeding-related HCRU**												
Outpatient Visits	222	3650	93.67	910.17 (873.77 - 946.57)	110	1867	96.49	902.34 (847.85 - 956.83)	118	1927	99.16	874.53 (815.01 - 934.05)
GPs visits	222	2707	93.67	675.02 (615.39 - 734.65)	110	1397	96.49	675.18 (589.21 - 761.15)	118	1431	99.16	649.43 (563.7 - 735.16)
Specialist visits	218	943	91.98	235.15 (181.16 - 289.14)	109	470	95.61	227.16 (150.24 - 304.08)	116	496	97.48	225.1 (150.06 - 300.14)
Investigations[3]	237	825	100.00	205.72 (154.26 - 257.18)	114	448	100.00	216.52 (140.91 - 292.13)	119	519	100.00	235.54 (159.3 - 311.78)
Hospitalization	237	260	100.00	64.83 (58.75 - 70.91)	114	133	100.00	64.28 (55.48 - 73.08)	119	137	100.00	62.17 (53.46 - 70.88)

Type of FXai indication.

Time window[1]	Venous thromboembolism (N=83)				Atrial fibrillation (N=367)				Non-mechanical cardiac-valve replacement (N=20)			
	Patients (n)	Visits (n)	% of patients	Rate, number of visits per 100 patient-years (95 CI)	Patients (n)	Visits (n)	% of patients	Rate, number of visits per 100 patient-years (95 CI)	Patients (n)	Visits (n)	% of patients	Rate, number of visits per 100 patient-years (95 CI)
**Cumulative HCRU**												
**6 months since index date**												
**All-cause HCRU**												
Outpatient Visits	78	778	93.98	2026.19 (1161.44 - 2890.94)	349	3390	95.10	2001.05 (1591.72 - 2410.38)	17	145	85.00	1699.58 (53.46 - 3345.7)
GPs visits	75	582	90.36	1515.73 (744.23 - 2287.23)	341	2550	92.92	1505.22 (1139.37 - 1871.07)	16	102	80.00	1195.57 (0 - 2617.5)
Specialist visits	65	196	78.31	510.45 (402.9 - 618)	303	840	82.56	495.84 (444.69 - 546.99)	14	43	70.00	504.01 (284.88 - 723.14)
Investigations[3]	83	246	100.00	640.67 (537.45 - 743.89)	367	978	100.00	577.29 (526.75 - 627.83)	20	47	100.00	550.9 (332.9 - 768.9)
Hospitalization	83	103	100.00	268.25 (172.93 - 363.57)	367	453	100.00	267.4 (222.12 - 312.68)	20	23	100.00	269.59 (75.11 - 464.07)
**Bleeding-related HCRU**												
Outpatient Visits	78	716	93.98	1864.72 (1026.79 - 2702.65)	349	3102	95.10	1831.05 (1435.36 - 2226.74)	17	135	85.00	1582.37 (0 - 3181.89)
GPs visits	75	524	90.36	1364.68 (626.14 - 2103.22)	341	2278	92.92	1344.66 (995.62 - 1693.7)	16	92	80.00	1078.36 (0 - 2437.75)
Specialist visits	65	192	78.31	500.04 (392.47 - 607.61)	303	824	82.56	486.39 (435.25 - 537.53)	14	43	70.00	504.01 (284.88 - 723.14)
Investigations[3]	83	162	100.00	421.91 (315.66 - 528.16)	367	645	100.00	380.73 (331.05 - 430.41)	20	31	100.00	363.36 (152.57 - 574.15)
Hospitalization	83	86	100.00	223.97 (134.28 - 313.66)	367	379	100.00	223.72 (181.08 - 266.36)	20	21	100.00	246.15 (57.36 - 434.94)
**1 year since index date**												
**All-cause HCRU**												
Outpatient Visits	79	1175	95.18	1689.42 (883.3 - 2495.54)	352	5211	95.91	1731.62 (1344.49 - 2118.75)	17	232	85.00	1452.49 (0 - 2996.74)
GPs visits	78	871	93.98	1252.32 (540.25 - 1964.39)	352	3911	95.91	1299.63 (955.6 - 1643.66)	17	163	85.00	1020.5 (0 - 2347.2)
Specialist visits	75	304	90.36	437.09 (330.38 - 543.8)	337	1300	91.83	431.99 (381.31 - 482.67)	16	69	80.00	431.99 (214.89 - 649.09)
Investigations[3]	83	369	100.00	530.55 (423.18 - 637.92)	367	1523	100.00	506.1 (454.95 - 557.25)	20	81	100.00	507.12 (288.01 - 726.23)
Hospitalization	83	110	100.00	158.16 (79.66 - 236.66)	367	496	100.00	164.82 (126.86 - 202.78)	20	23	100.00	144 (0 - 297.87)
**Bleeding-related HCRU**												
Outpatient Visits	79	1064	95.18	1529.82 (755.39 - 2304.25)	352	4594	95.91	1526.59 (1158.62 - 1894.56)	17	208	85.00	1302.23 (0 - 2777.22)
GPs visits	78	802	93.98	1153.12 (465.97 - 1840.27)	352	3461	95.91	1150.1 (823.69 - 1476.51)	17	142	85.00	889.02 (751.36 - 1026.68)
Specialist visits	75	262	90.36	376.7 (272.45 - 480.95)	337	1133	91.83	376.5 (326.93 - 426.07)	16	66	80.00	413.21 (197.4 - 629.02)
Investigations[3]	83	216	100.00	310.56 (211.01 - 410.11)	367	829	100.00	275.48 (229.77 - 321.19)	20	35	100.00	219.13 (37.84 - 400.42)

Hospitalization	83	87	100.00	125.09 (53.92 - 196.26)	367	397	100.00	131.92 (97.3 - 166.54)	20	21	100.00	131.48 (0 - 279.58)
**2 years since index date**												
**All-cause HCRU**												
Outpatient Visits	80	1658	96.39	1434.02 (680 - 2188.04)	353	7104	96.19	1411.94 (1055.67 - 1768.21)	17	323	85.00	1134.15 (0 - 2523.9)
GPs visits	80	1243	96.39	1075.08 (408.67 - 1741.49)	353	5390	96.19	1071.28 (754.86 - 1387.7)	17	245	85.00	860.27 (708.32 - 1012.22)
Specialist visits	79	415	95.18	358.94 (255.74 - 462.14)	347	1714	94.55	340.66 (292.17 - 389.15)	17	78	85.00	273.88 (78.43 - 469.33)
Investigations[3]	83	441	100.00	381.42 (276.92 - 485.92)	367	1849	100.00	367.49 (318.16 - 416.82)	20	97	100.00	340.6 (132.9 - 548.3)
Hospitalization	83	116	100.00	100.33 (35.69 - 164.97)	367	529	100.00	105.14 (73.76 - 136.52)	20	24	100.00	84.27 (68.31 - 100.23)
**Bleeding-related HCRU**												
Outpatient Visits	80	1252	96.39	1082.87 (414.34 - 1751.4)	353	5329	96.19	1059.15 (744.31 - 1373.99)	17	233	85.00	818.13 (649.07 - 987.19)
GPs visits	80	917	96.39	793.12 (705.97 - 880.27)	353	3935	96.19	782.09 (739.85 - 824.33)	17	169	85.00	593.41 (378.13 - 808.69)
Specialist visits	79	335	95.18	289.74 (192.14 - 387.34)	347	1394	94.55	277.06 (231.27 - 322.85)	17	64	85.00	224.72 (41.79 - 407.65)
Investigations[3]	83	320	100.00	276.77 (180.52 - 373.02)	367	1203	100.00	239.1 (195.46 - 282.74)	20	52	100.00	182.59 (13.27 - 351.91)
Hospitalization	83	90	100.00	77.84 (68.9 - 86.78)	367	406	100.00	80.69 (76.65 - 84.73)	20	21	100.00	73.74 (54.45 - 93.03)
**3 years since index date**												
**All-cause HCRU**												
Outpatient Visits	80	2127	96.39	1465.18 (704.4 - 2225.96)	353	9380	96.19	1453.12 (1092.56 - 1813.68)	17	402	85.00	1069.15 (0 - 2423.42)
GPs visits	80	1614	96.39	1111.8 (435.5 - 1788.1)	353	7238	96.19	1121.28 (798.46 - 1444.1)	17	308	85.00	819.15 (650.46 - 987.84)
Specialist visits	80	513	96.39	353.38 (250.54 - 456.22)	353	2142	96.19	331.83 (283.65 - 380.01)	17	94	85.00	250 (60.22 - 439.78)
Investigations[3]	83	516	100.00	355.45 (252.47 - 458.43)	367	2230	100.00	345.46 (296.81 - 394.11)	20	120	100.00	319.15 (114.85 - 523.45)
Hospitalization	83	118	100.00	81.28 (72.89 - 89.67)	367	548	100.00	84.89 (81.23 - 88.55)	20	24	100.00	63.83 (42.77 - 84.89)
**Bleeding-related HCRU**												
Outpatient Visits	80	1374	96.39	946.48 (898.06 - 994.9)	353	5815	96.19	900.84 (870.26 - 931.42)	17	255	85.00	678.19 (473.44 - 882.94)
GPs visits	80	1015	96.39	699.18 (600.51 - 797.85)	353	4334	96.19	671.41 (623.35 - 719.47)	17	186	85.00	494.68 (275.56 - 713.8)
Specialist visits	79	359	95.18	247.3 (154.48 - 340.12)	347	1481	94.55	229.43 (186.41 - 272.45)	17	69	85.00	183.51 (13.86 - 353.16)
-Investigations[3]	83	365	100.00	251.43 (158.1 - 344.76)	367	1367	100.00	211.77 (169.97 - 253.57)	20	60	100.00	159.57 (0 - 320.07)
Hospitalization	83	90	100.00	62.00 (51.56 - 72.44)	367	419	100.00	64.91 (60.03 - 69.79)	20	21	100.00	55.85 (34.09 - 77.61)

1. Index date: day of the first major bleeding; 2. Laboratory/radiology investigations. Qualitative variables are presented as absolute and relative (%) frequencies. Rate (95 CI): per 100 person-years. AMI: acute myocardial infarction; FXai; Factor Xa inhibitors; GIB: gastrointestinal bleeding; GP: general practitioners; HCRU: Health Care Resource Utilization; ICH: intracranial bleeding; MB: major bleeding.

Supplementary Table S6. Cumulative Length of hospital stays, number of prescriptions and work absences for 3 years from index date in the overall study population and according to the type of first major bleeding, the type of FXai and the FXai indication.

Overall study population.

Time window[1]	All FXai N=470			
	Patient with use of resources (n)	% of patients	ALL patients within cohort	
**Cumulative HCRU per patient**			Average	Standard deviation
**6 months since index date**				
**All-cause HCRU**				
Length of hospital stays (days)	470	100	13.28	5.99
Number of Prescriptions	470	100	8.52	3.90
Work absences - ALL (number of days)	40	9	2.56	9.34
**Bleeding-related HCRU**				
Length of hospital stays (days)	470	100	11.16	2.79
Number of Prescriptions - ALL	470	100	2.88	0.78
Work absences (number of days)	40	9	2.22	7.51
**1 year since index date**				
**All-cause HCRU**				
Length of hospital stays (days)	470	100	14.47	7.43
Number of Prescriptions	470	100	16.99	5.46
Work absences (number of days)	40	9	2.60	9.62
**Bleeding-related HCRU**				
Length of hospital stays (days)	470	100	11.63	3.84
Number of Prescriptions	470	100	3.38	1.26
Work absences (number of days)	40	9	2.28	7.81
**2 years since index date**				
**All-cause HCRU**				
Length of hospital stays (days)	470	100	15.34	8.10
Number of Prescriptions	470	100	25.70	6.81
Work absences (number of days)	40	9	2.94	10.84
**Bleeding-related HCRU**				
Length of hospital stays (days)	470	100	11.91	4.42
Number of Prescriptions	470	100	3.70	1.50
Work absences (number of days)	40	9	2.39	8.29
**3 years since index date**				
**All-cause HCRU**				
Length of hospital stays (days)	470	100	15.82	8.41
Number of Prescriptions	470	100	34.27	7.95
Work absences (number of days)	40	9	3.04	11.27
**Bleeding-related HCRU**				
Length of hospital stays (days)	470	100	12.20	4.81
Number of Prescriptions	470	100	3.86	1.64
Work absences (number of days)	40	9	2.49	8.65

Type of first major bleeding.

Time window[1]	GIB N=418				ICH N=32				Trauma bleeding N=10				Other MB N=20			
**Cumulative HCRU per patient**	**Patient with use of resources n**	**% of patients**	**Average**	**Standard deviation**	**Patient with use of resources n**	**% of patients**	**Average**	**Standard deviation**	**Patient with use of resources n**	**% of patients**	**Average**	**Standard deviation**	**Patient with use of resources n**	**% of patients**	**Average**	**Standard deviation**
**6 months since index date**																
**All-cause HCRU**																
Length of hospital stays (days)	418	100	13.30	6.02	32	100	13.84	6.79	10	100	11.10	2.69	20	100	11.95	3.30
Number of Prescriptions	418	100	8.52	3.92	32	100	8.66	3.65	10	100	8.20	4.34	20	100	8.30	4.07
Work absences - ALL (number of days)	34	8	2.52	9.49	4	13	3.13	8.44	3	30	8.00	12.89	2	10	2.45	7.56
**Bleeding-related HCRU**																
Length of hospital stays (days)	418	100	11.19	2.87	32	100	10.63	2.00	10	100	11.10	2.69	20	100	11.35	2.03
Number of Prescriptions - ALL	418	100	2.85	0.79	32	100	2.97	0.69	10	100	3.10	0.74	20	100	3.20	0.77
Work absences (number of days)	34	8	2.14	7.44	4	13	3.13	8.44	3	30	8.00	12.89	2	10	2.45	7.56
**1 year since index date**																
**All-cause HCRU**																
Length of hospital stays (days)	418	100	14.63	7.61	32	100	13.84	6.79	10	100	11.10	2.69	20	100	11.95	3.30
Number of Prescriptions	418	100	17.09	5.48	32	100	16.28	5.94	10	100	15.20	5.03	20	100	16.15	4.22
Work absences (number of days)	34	8	2.57	9.81	4	13	3.13	8.44	3	30	8.00	12.89	2	10	2.45	7.56
**Bleeding-related HCRU**																
Length of hospital stays (days)	418	100%	11.72	4.00	32	100%	10.63	2.00	10	100%	11.10	2.69	20	100%	11.35	2.03
Number of Prescriptions	418	100%	3.37	1.26	32	100	3.47	1.37	10	100	3.60	1.51	20	100	3.40	1.19
Work absences (number of days)	34	8	2.21	7.78	4	13	3.13	8.44	3	30	8.00	12.89	2	10	2.45	7.56
**2 years since index date**																
**All-cause HCRU**																
Length of hospital stays (days)	418	100	15.58	8.31	32	100	14.28	6.78	10	100	11.10	2.69	20	100	11.95	3.30
Number of Prescriptions	418	100	25.71	6.90	32	100	26.03	6.21	10	100	24.50	6.72	20	100	25.10	6.24
Work absences (number of days)	34	8	2.84	10.70	4	13	4.59	14.05	3	30	8.00	12.89	2	10	2.45	7.56
**Bleeding-related HCRU**																
Length of hospital stays (days)	418	100	12.04	4.62	32	100	10.63	2.00	10	100	11.10	2.69	20	100	11.35	2.03
Number of Prescriptions	418	100	3.67	1.49	32	100	4.25	1.80	10	100	4.20	1.55	20	100	3.50	1.15
Work absences (number of days)	34	8	2.33	8.33	4	13	3.13	8.44	3	30	8.00	12.89	2	10	2.45	7.56
**3 years since index date**																
**All-cause HCRU**																
Length of hospital stays (days)	418	100	16.07	8.64	32	100	14.69	6.97	10	100	11.10	2.69	20	100	12.45	3.72
Number of Prescriptions	418	100	34.20	8.07	32	100	35.25	6.60	10	100	33.20	8.00	20	100	34.20	7.48
Work absences (number of days)	34	8	2.94	11.20	4	13	4.59	14.05	3	30	8.00	12.89	2	10	2.45	7.56
**Bleeding-related HCRU**																
Length of hospital stays (days)	418	100	12.36	5.03	32	100	10.63	2.00	10	100	11.10	2.69	20	100	11.35	2.03
Number of Prescriptions	418	100	3.83	1.63	32	100	4.31	1.79	10	100	4.50	1.35	20	100	3.80	1.67
Work absences (number of days)	34	8	2.44	8.73	4	13	3.13	8.44	3	30	8.00	12.89	2	10	2.45	7.56

Type of FXai.

Time window[1]	Apixaban N=237				Rivaroxaban N=114				Edoxaban N=119			
**Cumulative HCRU per patient**	**Patient with use of resources n**	**% of patients**	**Average**	**Standard deviation**	**Patient with use of resources n**	**% of patients**	**Average**	**Standard deviation**	**Patient with use of resources n**	**% of patients**	**Average**	**Standard deviation**
**6 months since index date**												
**All-cause HCRU**												
Length of hospital stays (days)	237	100	13.76	6.28	114	100	12.85	5.66	119	100	12.73	5.66
Number of Prescriptions	237	100	8.56	4.00	114	100	8.35	3.91	119	100	8.60	3.70
Work absences - ALL (number of days)	20	8	2.59	9.60	10	9	2.93	10.67	10	8	2.13	7.26
**Bleeding-related HCRU**												
Length of hospital stays (days)	237	100	11.38	2.70	114	100	10.79	1.92	119	100	11.08	3.54
Number of Prescriptions - ALL	237	100	2.89	0.78	114	100	2.75	0.75	119	100	2.97	0.81
Work absences (number of days)	20	8	2.19	7.42	10	9	2.52	8.51	10	8	1.98	6.65
**1 year since index date**												
**All-cause HCRU**												
Length of hospital stays (days)	237	100	14.90	7.51	114	100	14.14	7.45	119	100	13.91	7.28
Number of Prescriptions	237	100	16.78	5.52	114	100	17.06	5.59	119	100	17.34	5.22
Work absences (number of days)	20	8	2.68	10.15	10	9	2.93	10.67	10	8	2.13	7.26
**Bleeding-related HCRU**												
Length of hospital stays (days)	237	100	11.77	3.47	114	100	11.18	2.83	119	100	11.77	5.15
Number of Prescriptions	237	100	3.33	1.23	114	100	3.38	1.29	119	100	3.47	1.30
Work absences (number of days)	20	8	2.31	8.02	10	9	2.52	8.51	10	8	1.98	6.65
**2 years since index date**												
**All-cause HCRU**												
Length of hospital stays (days)	237	100	15.62	8.02	114	100	15.30	8.13	119	100	14.82	8.25
Number of Prescriptions	237	100	25.41	6.94	114	100	25.91	6.54	119	100	26.09	6.84
Work absences (number of days)	20	8	3.07	11.46	10	9	3.32	11.79	10	8	2.32	8.40
**Bleeding-related HCRU**												
Length of hospital stays (days)	237	100	11.89	3.53	114	100	11.85	4.37	119	100	12.02	5.87
Number of Prescriptions	237	100	3.70	1.50	114	100	3.66	1.47	119	100	3.76	1.54
Work absences (number of days)	20	8	2.43	8.49	10	9	2.75	9.39	10	8	1.98	6.65
**3 years since index date**												
**All-cause HCRU**												
Length of hospital stays (days)	237	100	15.92	8.10	114	100	15.88	8.48	119	100	15.56	9.01
Number of Prescriptions	237	100	33.99	7.96	114	100	34.72	7.70	119	100	34.41	8.20
Work absences (number of days)	20	8	3.16	11.73	10	9	3.32	11.79	10	8	2.50	9.82
**Bleeding-related HCRU**												
Length of hospital stays (days)	237	100	12.00	3.57	114	100	12.34	5.31	119	100	12.45	6.27
Number of Prescriptions	237	100	3.84	1.66	114	100	3.76	1.56	119	100	3.98	1.69
Work absences (number of days)	20	8	2.52	8.86	10	9	2.75	9.39	10	8	2.18	7.49

Type of FXai indication.

Time window[1]	Venous thromboembolism N=83				Atrial fibrillation N=367				Non-mechanical cardiac-valve replacement N=20			
**Cumulative HCRU per patient**	**Patient with use of resources n**	**% of patients**	**Average**	**Standard deviation**	**Patient with use of resources n**	**% of patients**	**Average**	**Standard deviation**	**Patient with use of resources n**	**% of patients**	**Average**	**Standard deviation**
**6 months since index date**												
**All-cause HCRU**												
Length of hospital stays (days)	83	100	13.13	5.89	367	100	13.34	6.04	20	100	12.80	5.66
Number of Prescriptions	83	100	8.92	3.52	367	100	8.43	3.97	20	100	8.50	4.12
Work absences - ALL (number of days)	9	11	3.45	11.92	28	8	2.32	8.75	3	15	3.15	7.71
**Bleeding-related HCRU**												
Length of hospital stays (days)	83	100	11.02	2.59	367	100	11.17	2.78	20	100	11.55	3.68
Number of Prescriptions - ALL	83	100	3.04	0.79	367	100	2.84	0.78	20	100	2.90	0.79
Work absences (number of days)	9	11	2.82	8.44	28	8	2.03	7.28	3	15	3.15	7.71
**1 year since index date**												
**All-cause HCRU**												
Length of hospital stays (days)	83	100	14.01	6.79	367	100	14.66	7.65	20	100	12.80	5.66
Number of Prescriptions	83	100	17.54	5.19	367	100	16.89	5.56	20	100	16.60	4.74
Work absences (number of days)	9	11	3.45	11.92	28	8	2.38	9.14	3	15	3.15	7.71
**Bleeding-related HCRU**												
Length of hospital stays (days)	83	100	11.14	2.81	367	100	11.74	4.04	20	100	11.55	3.68
Number of Prescriptions	83	100	3.54	1.39	367	100	3.32	1.23	20	100	3.80	1.15
Work absences (number of days)	9	11	2.82	8.44	28	8	2.11	7.67	3	15	3.15	7.71
**2 years since index date**												
**All-cause HCRU**												
Length of hospital stays (days)	83	100	14.83	7.64	367	100	15.58	8.31	20	100	13.10	5.44
Number of Prescriptions	83	100	26.35	6.26	367	100	25.52	6.96	20	100	26.30	6.35
Work absences (number of days)	9	11	3.72	12.60	28	8	2.70	10.44	3	15	4.10	10.45
**Bleeding-related HCRU**												
Length of hospital stays (days)	83	100	11.54	3.49	367	100	12.01	4.64	20	100	11.55	3.68
Number of Prescriptions	83	100	3.92	1.59	367	100	3.64	1.49	20	100	4.05	1.36
Work absences (number of days)	9	11	3.14	9.63	28	8	2.18	8.00	3	15	3.15	7.71
**3 years since index date**												
**All-cause HCRU**												
Length of hospital stays (days)	83	100	15.10	7.90	367	100	16.13	8.64	20	100	13.10	5.44
Number of Prescriptions	83	100	34.78	7.69	367	100	34.17	8.03	20	100	34.15	7.79
Work absences (number of days)	9	11	3.72	12.60	28	8	2.82	11.02	3	15	4.10	10.45
**Bleeding-related HCRU**												
Length of hospital stays (days)	83	100	11.54	3.49	367	100	12.38	5.10	20	100	11.55	3.68
Number of Prescriptions	83	100	4.18	1.86	367	100	3.77	1.59	20	100	4.10	1.45
Work absences (number of days)	9	11	3.14	9.63	28	8	2.31	8.48	3	15	3.15	7.71

1. Index date: day of the first major bleeding; Quantitative variables are presented as mean and (Standard deviation): qualitative variables are presented as absolute and relative (%) frequencies. FXai; Factor Xa inhibitors; GIB: gastrointestinal bleeding; GP: general practitioners; HCRU: Health Care Resource Utilization; ICH: intracranial bleeding; MB: major bleeding.

Supplementary Table S7. Cumulative costs for 3 years from index date in the overall study population and according to the type of the first major bleeding, the type of FXai and the FXai indication.

Overall study population.

Time window1	All FXai			
**Cumulative costs**	No. Patients	Total cost (€)	Mean costs per patient (€)	SD costs per patient (€)
**6 months since index date**				
**All-cause costs**				
**Outpatient**	470	514,415.8	1094.50	515.15
GPs visit	470	207,364.1	441.20	254.17
Specialist visit	470	255,281.0	543.15	337.41
Investigations[3] (outpatient)	470	21,624.8	46.01	36.89
Prescriptions (outpatient)	470	29,764.9	63.33	39.58
**Inpatient (hospital+all other costs in hospitalization)**	470	4,936,654.7	10503.52	4325.64
Hospitalizations (>24 hours)	470	4,478,320.5	9528.34	4297.66
Investigations[3] (within hospital)	470	73,699.1	156.81	131.41
Prescriptions (within hospital)	470	383,745.1	816.48	578.34
**Pharmacy & Investigation (global)**	470	508,834.0	1082.63	604.74
Prescriptions (global)	470	413,510.0	879.81	581.25
Investigations[3] (global)	470	95,324.0	202.82	142.91
**Indirect cost**				
Cost of absence from work	470	329,132.7	700.28	2558.70
**Total Overall cost**	470	5,780,203.3	12298.30	4855.57
**Bleeding-related costs**				
**Outpatient**	470	450,116.4	957.69	479.22
GPs visits	470	185,563.3	394.82	223.03
Specialist visits	470	250,549.2	533.08	323.50
Investigations[3] (outpatient)	470	12,997.6	27.65	40.89
Prescriptions (outpatient)	470	777.4	1.65	3.91
**Inpatient - Bleeding episode (hospital+all other costs in bleeding event hospitalization)**	470	4,194,794.0	8925.09	2135.64
Hospitalizations (>24 hours)	470	3,764,229.4	8009.00	2000.76
Investigations[3] (within hospital)	470	46,641.3	99.24	78.59
Prescriptions (within hospital)	470	383,314.3	815.56	578.42
**Pharmacy & Investigation (global)**	470	443,730.6	944.11	584.17
Prescriptions	470	384,091.7	817.22	578.73
Investigations[3] (global)	470	59,638.9	126.89	86.12
**Indirect cost**				
Cost of absence from work	470	285,558.9	607.57	2056.87
**Total Overall bleeding cost**	470	4,930,469.4	10490.36	2587.72
**1 year since index date**				
**All-cause costs**				
**Outpatient**	470	813,977.5	1731.87	715.68
GPs visit	470	317,073.4	674.62	339.36
Specialist visit	470	395,815.6	842.16	448.56
Investigations[3] (outpatient)	470	40,411.8	85.98	62.54
Prescriptions (outpatient)	470	59,964.8	127.58	64.26
**Inpatient**	470	5,396,449.0	11481.81	5478.80
Hospitalizations (>24 hours)	470	4,879,503.4	10381.92	5335.64
Investigations[3] (within hospital)	470	94,191.6	200.41	150.08
Prescriptions (within hospital)	470	421,492.9	896.79	702.01
**Pharmacy & Investigation (global)**	470	616,061.1	1310.77	742.74

Prescriptions (global)	470	481,457.7	1024.38	706.27
Investigations[3] (global)	470	134,603.4	286.39	175.96
**Indirect cost**				
Cost of absence from work	470	335,161.8	713.11	2636.57
**Total Overall cost**	470	6,545,588.3	13926.78	5920.77
**Bleeding-related costs**				
**Outpatient**	470	662,667.9	1409.93	720.37
GPs visits	470	282,448.6	600.95	388.93
Specialist visits	470	345,658.5	735.44	481.12
Investigations[3] (outpatient)	470	21,965.4	46.73	74.33
Prescriptions (outpatient)	470	12,208.5	25.98	14.12
**Inpatient - Bleeding episode (hospital+all other costs in bleeding event hospitalization)**	470	4,371,611.6	9301.30	2984.35
Hospitalizations (>24 hours)	470	3,922,118.9	8344.93	2753.93
Investigations[3] (within hospital)	470	49,631.0	105.60	78.84
Prescriptions (within hospital)	470	399,168.7	849.30	634.17
**Pharmacy & Investigation (global)**	470	482,973.5	1027.60	643.25
Prescriptions	470	411,377.2	875.27	633.61
Investigations[3] (global)	470	71,596.4	152.33	113.97
**Indirect cost**				
Cost of absence from work	470	293,506.4	624.48	2139.32
**Total Overall bleeding cost**	470	5,327,785.8	11335.71	3321.55
**2 years since index date**				
**All-cause costs**				
**Outpatient**	470	1,101,334.8	2343.27	970.89
GPs visit	470	441,017.4	938.33	440.02
Specialist visit	470	522,154.9	1110.97	601.11
Investigations[3] (outpatient)	470	45,974.1	97.82	57.78
Prescriptions (outpatient)	470	91,378.6	194.42	92.10
**Inpatient**	470	5,746,930.6	12227.51	6099.89
Hospitalizations (>24 hours)	470	5,174,469.7	11009.51	5810.79
Investigations[3] (within hospital)	470	113,439.0	241.36	174.67
Prescriptions (within hospital)	470	457,444.8	973.29	863.32
**Pharmacy & Investigation (global)**	470	708,236.5	1506.89	914.77
Prescriptions (global)	470	548,823.4	1167.71	865.52
Investigations[3] (global)	470	159,413.1	339.18	200.81
**Indirect cost**				
Cost of absence from work	470	378,735.6	805.82	2970.05
**Total Overall cost**	470	7,227,001.0	15376.60	6753.09
**Bleeding-related costs**				
**Outpatient**	470	789,862.4	1680.56	787.48
GPs visits	470	321,946.5	684.99	327.48
Specialist visits	470	424,206.5	902.57	502.64
Investigations[3] (outpatient)	470	30,252.1	64.37	105.57
Prescriptions (outpatient)	470	12,924.4	27.50	15.79
**Inpatient - Bleeding episode (hospital+all other costs in bleeding event hospitalization)**	470	4,510,376.8	9596.55	3441.48
Hospitalizations (>24 hours)	470	4,017,570.3	8548.02	3171.39
Investigations[3] (within hospital)	470	72,328.0	153.89	105.86
Prescriptions (within hospital)	470	419,436.5	892.42	664.49
**Pharmacy & Investigation (global)**	470	534,940.9	1138.17	692.29
Prescriptions	470	432,360.8	919.92	664.53
Investigations[3] (global)	470	102,580.1	218.26	176.82
**Indirect cost**				
Cost of absence from work	470	308,305.0	655.97	2271.32

**Total Overall bleeding cost**	470	5,608,544.2	11933.07	3854.70
**3 years since index date**				
**All-cause costs**				
**Outpatient**	470	1,413,481.1	3007.41	1224.13
GPs visit	470	587,339.2	1249.66	631.16
Specialist visit	470	650,386.8	1383.80	674.18
Investigations[2] (outpatient)	470	53,693.2	114.24	57.42
Prescriptions (outpatient)	470	121,115.9	257.69	113.70
**Inpatient**	470	6,038,507.6	12847.89	6869.78
Hospitalizations (>24 hours)	470	5,335,947.6	11353.08	6038.97
Investigations[3] (within hospital)	470	137,655.3	292.88	200.22
Prescriptions (within hospital)	470	562,984.6	1197.84	1895.53
**Pharmacy & Investigation (global)**	470	875,449.0	1862.66	1946.94
Prescriptions (global)	470	684,100.5	1455.53	1901.91
Investigations[3] (global)	470	191,348.5	407.12	230.74
**Indirect cost**				
Cost of absence from work	470	391,067.8	832.06	3089.81
**Total Overall cost**	470	7,843,056.4	16687.35	7661.35
**Bleeding-related costs**				
**Outpatient**	470	857,818.3	1825.15	880.36
GPs visits	470	354,904.2	755.12	368.42
Specialist visits	470	451,650.9	960.96	550.29
Investigations[3] (outpatient)	470	37,857.6	80.55	136.09
Prescriptions (outpatient)	470	13,405.5	28.52	16.11
**Inpatient - Bleeding episode (hospital+all other costs in bleeding event hospitalization)**	470	4,638,200.9	9868.51	3779.94
Hospitalizations (>24 hours)	470	4,113,739.3	8752.64	3451.91
Investigations[3] (within hospital)	470	79,401.1	168.94	113.76
Prescriptions (within hospital)	470	443,935.5	944.54	703.76
**Pharmacy & Investigation (global)**	470	574,599.8	1222.55	736.63
Prescriptions	470	457,341.0	973.07	703.98
Investigations[3] (global)	470	117,258.8	249.49	204.30
**Indirect cost**				
Cost of absence from work	470	320,911.2	682.79	2371.46
**Total Overall bleeding cost**	470	5,816,930.5	12376.45	4259.28

Type of the first major bleeding.

Time window1	GIB				ICH				Trauma bleeding				Other MB			
**Cumulative costs**	**No. Patients**	**Total cost (€)**	**Mean costs per patient (€)**	**SD costs per patient (€)**	**No. Patients**	**Total cost (€)**	**Mean costs per patient (€)**	**SD costs per patient (€)**	**No. Patients**	**Total cost (€)**	**Mean costs per patient (€)**	**SD costs per patient (€)**	**No. Patients**	**Total cost (€)**	**Mean costs per patient (€)**	**SD costs per patient (€)**
**6 months since index date**																
**All-cause costs**																
**Outpatient**	418	465,750.7	1114.24	502.15	32	26,552.4	829.76	617.24	10	4,138.4	413.84	597.81	20	22,112.7	1105.63	517.94
GPs visit	418	187,615.1	448.84	248.71	32	11,028.6	344.65	286.64	10	1,859.5	185.95	335.84	20	8,720.3	436.02	293.17
Specialist visit	418	231,858.5	554.69	332.66	32	12,066.1	377.07	359.86	10	1,419.5	141.95	299.27	20	11,356.3	567.82	346.70
Investigations[3] (outpatient)	418	19,184.2	45.90	37.57	32	1,589.2	49.66	31.42	10	454.1	45.41	35.90	20	851.4	42.57	31.22
Prescriptions (outpatient)	418	26,754.9	64.01	39.80	32	1,840.4	57.51	38.54	10	397.4	39.74	10.10	20	1,169.7	58.48	37.35
**Inpatient (hospital+all other costs in hospitalization)**	418	4,402,459.4	10532.20	4343.64	32	347,040.3	10845.01	4953.59	10	89,381.3	8938.13	2426.04	20	187,155.0	9357.75	2480.38
Hospitalizations (>24 hours)	418	3,988,863.1	9542.73	4324.00	32	317,932.1	9935.38	4871.32	10	79,662.4	7966.24	1927.22	20	171,525.4	8576.27	2368.57
Investigations[3] (within hospital)	418	66,516.8	159.13	131.58	32	4,690.7	146.59	145.09	10	1,254.3	125.43	95.76	20	2,491.6	124.58	102.84
Prescriptions (within hospital)	418	346,276.6	828.41	584.12	32	24,368.5	761.52	524.53	10	8,449.5	844.95	595.24	20	13,100.0	655.00	532.17
**Pharmacy & Investigation (global)**	418	458,732.5	1097.45	611.62	32	32,488.9	1015.28	541.22	10	10,555.3	1055.53	595.32	20	17,612.6	880.63	532.83
Prescriptions (global)	418	373,031.5	892.42	587.23	32	26,208.9	819.03	527.98	10	8,846.9	884.69	600.20	20	14,269.6	713.48	526.92
Investigations[3] (global)	418	85,701.0	205.03	143.40	32	6,280.0	196.25	151.99	10	1,708.4	170.84	108.36	20	3,343.0	167.15	116.86
**Indirect cost**																
Cost of absence from work	418	288,299.4	689.71	2601.60	32	27,404.9	856.40	2312.58	10	21,923.9	2192.39	3533.24	20	13,428.4	671.42	2070.89
**Total Overall cost**	418	5,156,509.6	12336.15	4958.53	32	400,997.5	12531.17	4645.25	10	115,443.7	11544.37	2921.13	20	222,696.1	11134.80	2365.17
**Bleeding-related costs**																
**Outpatient**	418	409,283.4	979.15	468.69	32	21,851.3	682.85	553.66	10	3,207.8	320.78	568.52	20	18,981.7	949.09	458.28
GPs visits	418	168,379.1	402.82	219.36	32	9,618.0	300.56	245.08	10	1,667.1	166.71	288.66	20	7,566.2	378.31	238.92
Specialist visits	418	228,073.1	545.63	319.99	32	11,592.9	362.28	334.50	10	1,419.5	141.95	299.27	20	10,883.2	544.16	317.42
Investigations[3] (outpatient)	418	11,919.2	28.51	41.46	32	567.6	17.74	36.58	10	113.5	11.35	35.90	20	510.8	25.54	34.33
Prescriptions (outpatient)	418	702.0	1.68	3.94	32	62.8	1.96	4.01	10	5.6	0.56	1.77	20	12.6	0.63	2.82
**Inpatient - Bleeding episode (hospital+all other costs in bleeding event hospitalization)**	418	3,745,808.1	8961.26	2188.97	32	271,601.4	8487.54	1628.28	10	89,029.0	8902.90	2401.31	20	177,384.6	8869.23	1655.36
Hospitalizations (>24 hours)	418	3,357,305.0	8031.83	2059.11	32	244,011.1	7625.35	1432.46	10	79,662.4	7966.24	1927.22	20	162,913.3	8145.66	1459.24
Investigations[3] (within hospital)	418	42,061.5	100.63	80.33	32	3,210.6	100.33	71.54	10	914.2	91.42	64.66	20	1,369.2	68.46	37.82
Prescriptions (within hospital)	418	345,891.5	827.49	584.23	32	24,343.8	760.74	523.91	10	8,441.4	844.14	594.97	20	13,079.1	653.95	532.99
**Pharmacy & Investigation (global)**	418	400,574.2	958.31	590.29	32	28,184.7	880.77	528.87	10	9,474.7	947.47	586.99	20	14,971.7	748.59	518.10
Prescriptions	418	346,593.5	829.17	584.50	32	24,406.6	762.71	524.58	10	8,447.0	844.70	595.86	20	13,091.7	654.58	533.24
Investigations[3] (global)	418	53,980.7	129.14	87.37	32	3,778.1	118.07	81.16	10	1,027.7	102.77	67.79	20	1,880.0	94.00	58.47
**Indirect cost**																
Cost of absence from work	418	244,725.7	585.47	2039.58	32	27,404.9	856.40	2312.58	10	21,923.9	2192.39	3533.24	20	13,428.4	671.42	2070.89
**Total Overall bleeding cost**	418	4,399,817.1	10525.88	2654.61	32	320,857.6	10026.80	2084.17	10	114,160.7	11416.07	2923.33	20	209,794.7	10489.73	1776.03
**1 year since index date**																
**All-cause costs**																
**Outpatient**	418	739,867.4	1770.02	683.38	32	40,404.9	1262.65	928.11	10	6,960.2	696.02	973.75	20	33,705.2	1685.26	763.67
GPs visit	418	288,091.2	689.21	329.99	32	15,837.6	494.93	407.56	10	2,885.4	288.54	517.61	20	13,144.6	657.23	349.89
Specialist visit	418	360,800.2	863.16	434.02	32	18,454.0	576.69	537.29	10	2,129.3	212.93	479.06	20	16,561.3	828.07	482.43
Investigations[3] (outpatient)	418	36,325.2	86.90	63.87	32	2,440.6	76.27	46.96	10	1,021.6	102.16	52.16	20	1,646.0	82.30	56.68
Prescriptions (outpatient)	418	54,010.9	129.21	64.30	32	3,629.7	113.43	68.11	10	905.8	90.58	45.39	20	2,324.3	116.21	55.68
**Inpatient**	418	4,860,104.9	11627.05	5603.71	32	348,674.1	10896.07	4947.84	10	89,764.4	8976.44	2411.33	20	187,670.0	9383.50	2480.37
Hospitalizations (>24 hours)	418	4,390,046.0	10502.50	5460.15	32	317,932.1	9935.38	4871.32	10	79,662.4	7966.24	1927.22	20	171,525.4	8576.27	2368.57
Investigations[3] (within hospital)	418	84,898.5	203.11	152.55	32	6,297.6	196.80	143.98	10	1,630.4	163.04	80.81	20	2,995.6	149.78	92.26
Prescriptions (within hospital)	418	384,024.4	918.72	718.49	32	24,368.5	761.52	524.53	10	8,449.5	844.95	595.24	20	13,100.0	655.00	532.17

**Pharmacy & Investigation (global)**	418	559,259.0	1337.94	758.51	32	36,736.3	1148.01	558.07	10	12,007.4	1200.74	607.21	20	20,065.8	1003.29	567.06
Prescriptions (global)	418	438,035.3	1047.93	721.38	32	27,998.1	874.94	545.61	10	9,355.3	935.53	609.32	20	15,424.2	771.21	537.32
Investigations[3] (global)	418	121,223.7	290.01	179.94	32	8,738.2	273.07	150.22	10	2,652.1	265.21	117.31	20	4,641.5	232.08	114.71
**Indirect cost**																
Cost of absence from work	418	294,328.5	704.14	2687.68	32	27,404.9	856.40	2312.58	10	21,923.9	2192.39	3533.24	20	13,428.4	671.42	2070.89
**Total Overall cost**	418	5,894,300.8	14101.20	6100.11	32	416,484.0	13015.12	4684.26	10	118,648.5	11864.85	3043.48	20	234,803.5	11740.18	2392.21
**Bleeding-related costs**																
**Outpatient**	418	601,717.8	1439.52	705.42	32	31,466.2	983.32	799.44	10	4,530.5	453.05	736.81	20	29,483.9	1474.20	717.79
GPs visits	418	256,736.5	614.20	386.43	32	13,785.8	430.81	385.15	10	2,180.1	218.01	356.62	20	11,926.3	596.32	402.37
Specialist visits	418	313,718.8	750.52	474.26	32	15,851.6	495.36	511.19	10	1,892.7	189.27	414.31	20	16,088.1	804.41	493.90
Investigations[3] (outpatient)	418	20,205.9	48.34	75.65	32	908.1	28.38	61.16	10	227.0	22.70	71.79	20	851.4	42.57	63.46
Prescriptions (outpatient)	418	10,700.7	25.60	13.96	32	904.7	28.27	14.36	10	226.7	22.67	10.33	20	603.1	30.16	16.70
**Inpatient - Bleeding episode (hospital+all other costs in bleeding event hospitalization)**	418	3,923,258.7	9385.79	3102.54	32	270,921.0	8466.28	1627.68	10	88,732.3	8873.23	2399.94	20	177,431.9	8871.60	1654.69
Hospitalizations (>24 hours)	418	3,515,194.6	8409.56	2869.58	32	244,011.1	7625.35	1432.46	10	79,662.4	7966.24	1927.22	20	162,913.3	8145.66	1459.24
Investigations[3] (within hospital)	418	45,692.2	109.31	81.17	32	2,526.1	78.94	55.14	10	617.4	61.74	31.13	20	1,412.6	70.63	32.95
Prescriptions (within hospital)	418	361,745.9	865.42	645.20	32	24,343.8	760.74	523.91	10	8,441.4	844.14	594.97	20	13,079.1	653.95	532.99
**Pharmacy & Investigation (global)**	418	438,344.6	1048.67	653.92	32	28,682.7	896.34	532.96	10	9,512.6	951.26	594.40	20	15,946.2	797.31	520.03
Prescriptions	418	372,446.5	891.02	644.81	32	25,248.5	789.02	524.72	10	8,668.1	866.81	593.10	20	13,682.2	684.11	528.07
Investigations[3] (global)	418	65,898.1	157.65	115.85	32	3,434.3	107.32	96.45	10	844.5	84.45	73.05	20	2,264.0	113.20	72.47
**Indirect cost**																
Cost of absence from work	418	252,673.1	604.48	2133.03	32	27,404.9	856.40	2312.58	10	21,923.9	2192.39	3533.24	20	13,428.4	671.42	2070.89
**Total Overall bleeding cost**	418	4,777,649.5	11429.78	3439.76	32	329,792.0	10306.00	2110.88	10	115,186.7	11518.67	3055.03	20	220,344.3	11017.21	1815.33
**2 years since index date**																
**All-cause costs**																
**Outpatient**	418	1,003,200.4	2400.00	930.70	32	52,667.4	1645.86	1175.54	10	8,641.1	864.11	1171.60	20	45,467.0	2273.35	1052.19
GPs visit	418	402,160.6	962.11	424.19	32	20,069.6	627.17	495.26	10	3,206.0	320.60	613.53	20	18,787.2	939.36	504.57
Specialist visit	418	477,202.7	1141.63	584.17	32	23,895.6	746.74	723.90	10	2,602.5	260.25	540.09	20	21,056.5	1052.83	571.72
Investigations[3] (outpatient)	418	41,263.1	98.72	59.39	32	2,837.9	88.68	40.62	10	1,021.6	102.16	52.16	20	1,873.0	93.65	46.13
Prescriptions (outpatient)	418	81,847.0	195.81	93.27	32	5,814.4	181.70	74.12	10	1,793.0	179.30	83.82	20	3,717.3	185.86	95.36
**Inpatient**	418	5,196,519.8	12431.87	6260.57	32	362,097.3	11315.54	5039.19	10	79,662.4	7966.24	1927.22	20	171,525.4	8576.27	2368.57
Hospitalizations (>24 hours)	418	4,674,964.7	11184.13	5966.52	32	327,979.6	10249.36	4868.34	10	1,630.4	163.04	80.81	20	3,630.1	181.50	119.32
Investigations[3] (within hospital)	418	102,407.0	244.99	171.78	32	7,402.0	231.31	231.00	10	8,449.5	844.95	595.24	20	13,100.0	655.00	532.17
Prescriptions (within hospital)	418	417,717.1	999.32	884.30	32	26,627.8	832.12	698.79	10	12,894.6	1289.46	578.36	20	22,320.3	1116.02	559.98
**Pharmacy & Investigation (global)**	418	643,234.1	1538.84	937.09	32	42,682.0	1333.81	715.76	10	10,242.5	1024.25	582.46	20	16,817.2	840.86	547.35
Prescriptions (global)	418	499,564.0	1195.13	886.36	32	32,442.1	1013.82	691.69	10	2,652.1	265.21	117.31	20	5,503.1	275.16	129.21
Investigations[3] (global)	418	143,670.1	343.71	199.80	32	10,239.9	320.00	244.03								
**Indirect cost**									10	21,923.9	2192.39	3533.24	20	13,428.4	671.42	2070.89
Cost of absence from work	418	325,022.0	777.56	2933.57	32	40,285.2	1258.91	3851.09	10	120,329.5	12032.95	3089.19	20	247,208.9	12360.44	2462.75
**Total Overall cost**	418	6,524,742.2	15609.43	6870.53	32	455,049.9	14220.31	6578.59	10	79,662.4	7966.24	1927.22	20	171,525.4	8576.27	2368.57
**Bleeding-related costs**																
**Outpatient**	418	721,082.7	1725.08	753.73	32	35,945.3	1123.29	973.77	10	5,274.7	527.47	968.99	20	32,834.3	1641.72	850.55
GPs visits	418	293,733.7	702.71	316.97	32	14,619.4	456.86	363.36	10	2,372.4	237.24	433.88	20	13,593.4	679.67	363.55
Specialist visits	418	387,535.0	927.12	485.95	32	19,163.8	598.87	621.57	10	2,365.9	236.59	473.18	20	17,507.7	875.38	503.93
Investigations[3] (outpatient)	418	27,925.0	66.81	108.04	32	1,191.9	37.25	79.76	10	283.8	28.38	89.74	20	1,135.2	56.76	84.39
Prescriptions (outpatient)	418	11,397.1	27.27	15.98	32	949.2	29.66	16.03	10	247.6	24.76	10.17	20	578.1	28.90	11.06
**Inpatient - Bleeding episode (hospital+all other costs in bleeding event hospitalization)**	418	4,057,705.9	9707.43	3588.70	32	273,438.7	8544.96	1634.69	10	89,332.9	8933.29	2425.16	20	179,232.1	8961.61	1668.12
Hospitalizations (>24 hours)	418	3,610,645.9	8637.91	3313.79	32	244,011.1	7625.35	1432.46	10	79,662.4	7966.24	1927.22	20	162,913.3	8145.66	1459.24
Investigations[3] (within hospital)	418	65,603.2	156.95	104.62	32	4,126.2	128.94	124.24	10	989.4	98.94	80.09	20	2,598.7	129.93	96.79
Prescriptions (within hospital)	418	380,505.8	910.30	678.39	32	25,248.5	789.02	524.72	10	8,668.1	866.81	593.10	20	13,682.2	684.11	528.07
**Pharmacy & Investigation (global)**	418	485,431.1	1161.32	704.58	32	31,515.8	984.87	570.26	10	10,188.8	1018.88	621.65	20	17,994.1	899.70	541.44

Prescriptions	418	391,902.9	937.57	678.62	32	26,197.7	818.68	523.76	10	8,915.7	891.57	589.93	20	14,260.2	713.01	525.80
Investigations³ (global)	418	93,528.2	223.75	177.80	32	5,318.1	166.19	165.94	10	1,273.2	127.32	165.64	20	3,733.8	186.69	163.57
**Indirect cost**																
Cost of absence from work	418	267,471.7	639.88	2281.64	32	27,404.9	856.40	2312.58	10	21,923.9	2192.39	3533.24	20	13,428.4	671.42	2070.89
**Total Overall bleeding cost**	418	5,046,260.4	12072.39	3998.48	32	336,788.9	10524.65	2272.09	10	116,531.5	11653.15	3051.38	20	225,494.8	11274.74	1799.43
**3 years since index date**																
**All-cause costs**																
**Outpatient**	418	1,289,500.0	3084.93	1177.42	32	68,098.0	2128.06	1503.08	10	10,408.4	1040.84	1327.59	20	55,883.0	2794.15	1180.84
GPs visit	418	538,928.6	1289.30	617.80	32	26,673.9	833.56	691.94	10	3,590.7	359.07	693.37	20	21,736.7	1086.83	566.86
Specialist visit	418	593,841.7	1420.67	653.14	32	30,047.0	938.97	833.74	10	3,312.3	331.23	632.88	20	26,498.1	1324.91	596.56
Investigations³ (outpatient)	418	48,471.4	115.96	58.52	32	3,064.9	95.78	44.29	10	1,021.6	102.16	52.16	20	2,156.8	107.84	48.37
Prescriptions (outpatient)	418	107,404.3	256.95	114.07	32	8,258.2	258.07	111.51	10	2,465.8	246.58	108.52	20	5,453.4	272.67	113.99
**Inpatient**	418	5,461,489.9	13065.77	7059.88	32	379,561.5	11861.30	5557.33	10	89,764.4	8976.44	2411.33	20	197,456.2	9872.81	2941.90
Hospitalizations (>24 hours)	418	4,819,936.0	11530.95	6202.47	32	337,309.4	10540.92	5000.34	10	79,662.4	7966.24	1927.22	20	178,702.2	8935.11	2669.87
Investigations³ (within hospital)	418	124,619.5	298.13	199.46	32	8,582.4	268.20	237.57	10	1,630.4	163.04	80.81	20	4,453.4	222.67	132.14
Prescriptions (within hospital)	418	515,188.4	1232.51	1944.98	32	33,566.6	1048.96	1741.64	10	8,449.5	844.95	595.24	20	14,229.6	711.48	635.18
**Pharmacy & Investigation (global)**	418	795,683.6	1903.55	2000.82	32	53,472.2	1671.01	1724.55	10	13,567.4	1356.74	620.00	20	26,293.2	1314.66	662.14
Prescriptions (global)	418	622,592.7	1489.46	1953.05	32	41,824.8	1307.03	1721.68	10	10,915.3	1091.53	624.04	20	19,683.0	984.15	663.45
Investigations³ (global)	418	173,090.9	414.09	231.45	32	11,647.4	363.98	252.91	10	2,652.1	265.21	117.31	20	6,610.2	330.51	154.40
**Indirect cost**																
Cost of absence from work	418	337,354.2	807.07	3069.80	32	40,285.2	1258.91	3851.09	10	21,923.9	2192.39	3533.24	20	13,428.4	671.42	2070.89
**Total Overall cost**	418	7,088,344.1	16957.76	7781.53	32	487,944.7	15248.27	7577.36	10	122,096.7	12209.67	3218.27	20	266,767.6	13338.38	3053.72
**Bleeding-related costs**																
**Outpatient**	418	783,237.9	1873.77	847.93	32	38,454.0	1201.69	1048.52	10	5,594.7	559.47	1030.34	20	36,126.4	1806.32	922.28
GPs visits	418	323,934.2	774.96	356.70	32	15,709.4	490.92	394.80	10	2,564.8	256.48	472.15	20	15,260.6	763.03	421.18
Specialist visits	418	412,376.9	986.55	534.58	32	20,346.8	635.84	662.23	10	2,365.9	236.59	473.18	20	18,927.2	946.36	542.78
Investigations³ (outpatient)	418	35,019.7	83.78	139.59	32	1,419.0	44.34	97.50	10	340.5	34.05	107.69	20	1,419.0	70.95	107.18
Prescriptions (outpatient)	418	11,907.0	28.49	16.42	32	978.9	30.59	14.82	10	323.4	32.34	7.07	20	519.7	25.99	10.77
**Inpatient - Bleeding episode (hospital+all other costs in bleeding event hospitalization)**	418	4,182,823.5	10006.75	3945.35	32	274,802.2	8587.57	1654.31	10	89,622.5	8962.25	2434.09	20	180,575.2	9028.76	1633.61
Hospitalizations (>24 hours)	418	3,706,815.0	8867.98	3608.80	32	244,011.1	7625.35	1432.46	10	79,662.4	7966.24	1927.22	20	162,913.3	8145.66	1459.24
Investigations³ (within hospital)	418	71,505.0	171.06	113.14	32	4,536.4	141.76	117.22	10	1,031.4	103.14	96.06	20	3,359.7	167.99	121.42
Prescriptions (within hospital)	418	403,477.6	965.26	721.00	32	26,197.7	818.68	523.76	10	8,915.7	891.57	589.93	20	14,260.2	713.01	525.80
**Pharmacy & Investigation (global)**	418	521,909.2	1248.59	753.11	32	33,131.9	1035.37	572.76	10	10,611.0	1061.10	632.99	20	19,558.6	977.93	523.56
Prescriptions	418	415,384.5	993.74	720.94	32	27,176.5	849.27	526.97	10	9,239.1	923.91	592.89	20	14,780.0	739.00	528.96
Investigations³ (global)	418	106,524.7	254.84	206.50	32	5,955.4	186.11	163.32	10	1,372.0	137.20	189.92	20	4,778.7	238.93	208.09
**Indirect cost**																
Cost of absence from work	418	280,078.0	670.04	2393.67	32	27,404.9	856.40	2312.58	10	21,923.9	2192.39	3533.24	20	13,428.4	671.42	2070.89
**Total Overall bleeding cost**	418	5,246,139.4	12550.57	4422.80	32	340,661.0	10645.66	2309.81	10	117,141.1	11714.11	3048.42	20	230,130.1	11506.50	1810.39

Type of FXai.

Cumulative costs	Apixaban				Rivaroxaban				Edoxaban			
	**No. Patients**	**Total cost (€)**	**Mean costs per patient (€)**	**SD costs per patient (€)**	**No. Patients**	**Total cost (€)**	**Mean costs per patient (€)**	**SD costs per patient (€)**	**No. Patients**	**Total cost (€)**	**Mean costs per patient (€)**	**SD costs per patient (€)**
**6 months since index date**												
**All-cause costs**												
**Outpatient**	237	254,389.8	1073.37	542.54	114	127,105.2	1114.96	492.54	119	132,920.7	1116.98	481.60
GPs visit	237	102,848.5	433.96	257.44	114	51,937.2	455.59	269.40	119	52,578.4	441.84	233.26
Specialist visit	237	125,866.1	531.08	353.15	114	62,223.3	545.82	328.81	119	67,191.7	564.64	314.28
Investigations[3] (outpatient)	237	10,613.8	44.78	35.72	114	5,562.3	48.79	33.67	119	5,448.8	45.79	41.98
Prescriptions (outpatient)	237	14,874.5	62.76	39.18	114	7,284.5	63.90	39.50	119	7,605.9	63.92	40.76
**Inpatient (hospital+all other costs in hospitalization)**	237	2,574,299.2	10862.02	4491.10	114	1,159,475.5	10170.84	4119.50	119	1,202,880.0	10108.24	4153.94
Hospitalizations (>24 hours)	237	2,339,635.4	9871.88	4509.05	114	1,051,400.6	9222.81	4060.07	119	1,087,284.6	9136.85	4059.48
Investigations[3] (within hospital)	237	34,483.7	145.50	118.72	114	19,324.6	169.51	135.62	119	19,890.9	167.15	149.39
Prescriptions (within hospital)	237	199,750.1	842.83	612.01	114	88,533.3	776.61	543.90	119	95,461.6	802.20	542.08
**Pharmacy & Investigation (global)**	237	259,722.1	1095.87	632.54	114	120,704.7	1058.81	563.30	119	128,407.1	1079.05	590.38
Prescriptions (global)	237	214,624.7	905.59	612.76	114	95,817.8	840.51	548.83	119	103,067.5	866.11	548.06
Investigations[3] (global)	237	45,097.4	190.28	131.49	114	24,886.9	218.31	140.21	119	25,339.6	212.94	164.69
**Indirect cost**												
Cost of absence from work	237	167,992.0	708.83	2631.94	114	91,532.3	802.92	2923.25	119	69,608.4	584.94	1989.97
**Total Overall cost**	237	2,996,681.0	12644.22	5090.96	114	1,378,113.1	12088.71	5036.46	119	1,405,409.2	11810.16	4129.99
**Bleeding-related costs**												
**Outpatient**	237	221,556.3	934.84	507.10	114	111,112.7	974.67	455.47	119	117,447.4	986.95	444.54
GPs visits	237	91,691.6	386.88	224.22	114	46,743.5	410.03	243.32	119	47,128.2	396.04	200.28
Specialist visits	237	123,500.2	521.10	341.01	114	60,803.7	533.37	306.48	119	66,245.3	556.68	304.28
Investigations[3] (outpatient)	237	5,846.1	24.67	39.14	114	3,348.7	29.37	39.40	119	3,802.8	31.96	45.33
Prescriptions (outpatient)	237	415.5	1.75	3.94	114	157.8	1.38	3.68	119	204.1	1.72	4.06
**Inpatient - Bleeding episode (hospital+all other costs in bleeding event hospitalization)**	237	2,157,486.2	9103.32	2084.89	114	983,054.8	8623.29	1454.83	119	1,054,253.1	8859.27	2692.84
Hospitalizations (>24 hours)	237	1,934,864.1	8163.98	1939.50	114	882,745.9	7743.38	1375.27	119	946,619.4	7954.78	2540.96
Investigations[3] (within hospital)	237	22,773.5	96.09	75.27	114	11,762.5	103.18	86.20	119	12,105.3	101.73	77.84
Prescriptions (within hospital)	237	199,549.6	841.98	612.18	114	88,401.3	775.45	543.77	119	95,363.4	801.37	542.17
**Pharmacy & Investigation (global)**	237	228,584.6	964.49	615.67	114	103,670.4	909.39	554.40	119	111,475.6	936.77	549.40
Prescriptions	237	199,965.1	843.73	612.43	114	88,559.1	776.83	544.21	119	95,567.5	803.09	542.44
Investigations[3] (global)	237	28,619.5	120.76	83.18	114	15,111.3	132.56	85.74	119	15,908.1	133.68	91.91
**Indirect cost**												
Cost of absence from work	237	142,231.4	600.13	2034.63	114	78,652.0	689.93	2331.44	119	64,675.5	543.49	1822.03
**Total Overall bleeding cost**	237	2,521,273.9	10638.29	2540.48	114	1,172,819.5	10287.89	2505.80	119	1,236,376.0	10389.71	2756.84
**1 year since index date**												
**All-cause costs**												
**Outpatient**	237	405,196.0	1709.69	766.80	114	200,352.9	1757.48	684.03	119	208,428.7	1751.50	640.12
GPs visit	237	157,158.1	663.11	357.18	114	77,777.6	682.26	341.34	119	82,137.7	690.23	300.72
Specialist visit	237	198,735.9	838.55	477.37	114	97,002.0	850.90	434.68	119	100,077.7	840.99	403.60
Investigations[3] (outpatient)	237	20,319.4	85.74	60.17	114	10,102.9	88.62	58.02	119	9,989.4	83.94	71.20
Prescriptions (outpatient)	237	28,624.6	120.78	66.28	114	15,292.4	134.14	64.00	119	16,047.8	134.86	59.31
**Inpatient**	237	2,798,613.0	11808.49	5475.83	114	1,280,823.6	11235.29	5525.72	119	1,317,012.4	11067.33	5445.22
Hospitalizations (>24 hours)	237	2,534,844.3	10695.55	5389.84	114	1,156,899.5	10148.24	5346.70	119	1,187,759.7	9981.17	5221.44
Investigations[3] (within hospital)	237	44,741.5	188.78	136.27	114	24,733.2	216.96	157.44	119	24,717.0	207.71	167.50
Prescriptions (within hospital)	237	218,404.2	921.54	735.24	114	98,876.9	867.34	692.52	119	104,211.7	875.73	644.85
**Pharmacy & Investigation (global)**	237	312,089.7	1316.83	773.01	114	149,005.4	1307.06	742.60	119	154,966.0	1302.24	685.03
Prescriptions (global)	237	247,028.8	1042.32	742.05	114	114,169.3	1001.49	702.03	119	120,259.6	1010.58	638.75
Investigations[3] (global)	237	65,060.9	274.52	169.15	114	34,836.1	305.58	170.94	119	34,706.4	291.65	192.85

**Indirect cost**												
Cost of absence from work	237	174,021.0	734.27	2780.36	114	91,532.3	802.92	2923.25	119	69,608.4	584.94	1989.97
**Total Overall cost**	237	3,377,830.0	14252.45	6075.20	114	1,572,708.8	13795.69	6196.86	119	1,595,049.5	13403.78	5313.67
**Bleeding-related costs**												
**Outpatient**	237	327,478.6	1381.77	748.41	114	166,486.6	1460.41	688.61	119	168,702.7	1417.67	695.78
GPs visits	237	140,807.5	594.12	388.51	114	74,251.0	651.32	433.34	119	67,390.1	566.30	340.25
Specialist visits	237	170,818.2	720.75	504.36	114	83,516.4	732.60	429.23	119	91,323.9	767.43	483.07
Investigations[3] (outpatient)	237	9,592.1	40.47	69.76	114	5,732.6	50.29	72.33	119	6,640.7	55.80	83.92
Prescriptions (outpatient)	237	6,091.8	25.70	13.89	114	2,885.7	25.31	13.73	119	3,231.0	27.15	14.99
**Inpatient - Bleeding episode (hospital+all other costs in bleeding event hospitalization)**	237	2,231,314.1	9414.83	2681.31	114	1,018,307.9	8932.53	2191.76	119	1,121,989.5	9428.48	4030.67
Hospitalizations (>24 hours)	237	2,001,608.3	8445.60	2493.23	114	915,041.5	8026.68	2028.93	119	1,005,469.1	8449.32	3693.23
Investigations[3] (within hospital)	237	24,618.3	103.87	71.44	114	11,535.3	101.19	88.11	119	13,477.4	113.26	83.54
Prescriptions (within hospital)	237	204,743.5	863.90	642.24	114	91,567.1	803.22	590.15	119	102,858.0	864.35	661.28
**Pharmacy & Investigation (global)**	237	245,045.7	1033.95	659.39	114	111,720.7	980.01	593.05	119	126,207.1	1060.56	659.39
Prescriptions	237	210,835.3	889.60	642.30	114	94,452.8	828.53	589.28	119	106,089.1	891.50	659.72
Investigations[3] (global)	237	34,210.4	144.35	113.32	114	17,267.9	151.47	111.01	119	20,118.1	169.06	117.19
**Indirect cost**												
Cost of absence from work	237	150,178.8	633.67	2197.10	114	78,652.0	689.93	2331.44	119	64,675.5	543.49	1822.03
**Total Overall bleeding cost**	237	2,708,971.6	11430.26	3130.99	114	1,263,446.5	11082.86	2969.58	119	1,355,367.7	11389.64	3964.41
**2 years since index date**												
**All-cause costs**												
**Outpatient**	237	548,641.7	2314.94	980.11	114	267,781.2	2348.96	970.87	119	284,911.9	2394.22	958.32
GPs visit	237	217,751.5	918.78	453.56	114	107,914.0	946.61	450.04	119	115,351.9	969.34	402.96
Specialist visit	237	261,668.9	1104.09	603.71	114	125,866.1	1104.09	601.06	119	134,619.9	1131.26	600.56
Investigations[3] (outpatient)	237	22,873.5	96.51	56.00	114	11,578.7	101.57	52.03	119	11,521.9	96.82	66.24
Prescriptions (outpatient)	237	45,944.8	193.86	93.38	114	22,218.5	194.90	85.63	119	23,215.3	195.09	96.16
**Inpatient**	237	2,946,470.1	12432.36	5984.61	114	1,396,063.1	12246.17	6313.04	119	1,404,397.4	11801.66	6150.14
Hospitalizations (>24 hours)	237	2,656,849.8	11210.34	5759.24	114	1,251,633.2	10979.24	5835.87	119	1,265,986.8	10638.54	5918.61
Investigations[3] (within hospital)	237	53,379.4	225.23	152.39	114	30,193.4	264.85	188.34	119	29,866.2	250.98	199.27
Prescriptions (within hospital)	237	235,471.9	993.55	894.40	114	113,840.5	998.60	975.77	119	108,132.4	908.68	667.07
**Pharmacy & Investigation (global)**	237	357,669.6	1509.15	939.54	114	177,831.1	1559.92	1032.25	119	172,735.8	1451.56	730.94
Prescriptions (global)	237	281,416.7	1187.41	898.13	114	136,059.1	1193.50	970.48	119	131,347.7	1103.76	675.80
Investigations[3] (global)	237	76,252.9	321.74	182.72	114	41,772.1	366.42	206.75	119	41,388.1	347.80	226.19
**Indirect cost**												
Cost of absence from work	237	199,233.5	840.65	3140.63	114	103,864.5	911.09	3230.37	119	75,637.5	635.61	2301.70
**Total Overall cost**	237	3,694,345.3	15587.96	6798.77	114	1,767,708.8	15506.22	7161.41	119	1,764,946.8	14831.49	6268.40
**Bleeding-related costs**												
**Outpatient**	237	388,945.8	1641.12	803.19	114	194,692.0	1707.82	771.08	119	206,224.6	1732.98	773.64
GPs visits	237	157,863.4	666.09	335.84	114	80,534.7	706.44	341.43	119	83,548.4	702.09	295.94
Specialist visits	237	211,511.8	892.45	512.10	114	102,916.8	902.78	495.24	119	109,777.9	922.50	494.16
Investigations[3] (outpatient)	237	12,884.1	54.36	98.00	114	7,832.6	68.71	100.31	119	9,535.4	80.13	122.41
Prescriptions (outpatient)	237	6,459.5	27.26	15.85	114	3,269.9	28.68	16.69	119	3,195.0	26.85	14.83
**Inpatient - Bleeding episode (hospital+all other costs in bleeding event hospitalization)**	237	2,269,695.0	9576.77	2737.16	114	1,088,016.8	9544.01	3523.86	119	1,152,664.9	9686.26	4492.99
Hospitalizations (>24 hours)	237	2,021,703.4	8530.39	2533.90	114	969,585.1	8505.13	3133.73	119	1,026,281.8	8624.22	4209.70
Investigations[3] (within hospital)	237	35,101.8	148.11	106.55	114	17,560.0	154.04	104.34	119	19,666.3	165.26	105.89
Prescriptions (within hospital)	237	212,388.8	896.16	652.73	114	100,612.7	882.57	701.26	119	106,434.9	894.41	657.12
**Pharmacy & Investigation (global)**	237	266,834.2	1125.88	694.09	114	129,275.2	1133.99	709.44	119	138,831.5	1166.65	676.93
Prescriptions	237	218,848.4	923.41	652.24	114	103,882.6	911.25	701.05	119	109,629.9	921.26	658.48
Investigations[3] (global)	237	47,985.9	202.47	175.10	114	25,392.6	222.74	166.41	119	29,201.6	245.39	187.53
**Indirect cost**												
Cost of absence from work	237	157,578.1	664.89	2326.75	114	86,051.4	754.84	2571.99	119	64,675.5	543.49	1822.03
**Total Overall bleeding cost**	237	2,816,218.9	11882.78	3351.64	114	1,368,760.2	12006.67	4268.68	119	1,423,565.1	11962.73	4373.72

**3 years since index date**												
**All-cause costs**												
**Outpatient**	237	698,400.6	2946.84	1231.19	114	348,924.8	3060.74	1209.02	119	366,155.6	3076.94	1228.58
GPs visit	237	289,758.3	1222.61	619.91	114	143,821.2	1261.59	669.65	119	153,759.8	1292.10	617.99
Specialist visit	237	322,472.6	1360.64	690.51	114	161,118.0	1413.32	658.33	119	166,796.2	1401.65	660.01
Investigations[3] (outpatient)	237	26,733.1	112.80	57.83	114	13,338.2	117.00	50.81	119	13,621.9	114.47	62.69
Prescriptions (outpatient)	237	58,965.7	248.80	102.81	114	30,412.5	266.78	123.24	119	31,737.7	266.70	124.00
**Inpatient**	237	3,068,477.3	12947.16	6762.43	114	1,483,712.5	13015.02	7133.10	119	1,486,317.8	12490.07	6871.25
Hospitalizations (>24 hours)	237	2,707,805.0	11425.34	5816.12	114	1,299,000.0	11394.74	6085.33	119	1,329,142.6	11169.27	6464.28
Investigations[3] (within hospital)	237	66,106.7	278.93	185.70	114	35,735.4	313.47	201.17	119	35,813.3	300.95	225.24
Prescriptions (within hospital)	237	293,614.6	1238.88	2207.32	114	148,507.1	1302.69	1974.43	119	120,863.0	1015.66	869.27
**Pharmacy & Investigation (global)**	237	445,420.0	1879.41	2264.42	114	227,993.1	1999.94	2017.84	119	202,036.0	1697.78	930.22
Prescriptions (global)	237	352,580.2	1487.68	2216.55	114	178,919.6	1569.47	1972.71	119	152,600.7	1282.36	882.77
Investigations[3] (global)	237	92,839.7	391.73	219.58	114	49,073.5	430.47	224.18	119	49,435.2	415.42	256.87
**Indirect cost**												
Cost of absence from work	237	205,536.7	867.24	3215.22	114	103,864.5	911.09	3230.37	119	81,666.6	686.27	2691.96
**Total Overall cost**	237	3,972,414.6	16761.24	7704.83	114	1,936,501.8	16986.86	7922.23	119	1,934,140.0	16253.28	7360.29
**Bleeding-related costs**												
**Outpatient**	237	419,294.0	1769.17	893.19	114	213,936.6	1876.64	858.01	119	224,587.7	1887.29	875.87
GPs visits	237	173,572.8	732.37	378.88	114	89,575.6	785.75	379.56	119	91,755.7	771.06	335.00
Specialist visits	237	223,104.7	941.37	553.76	114	111,197.5	975.42	549.82	119	117,348.8	986.12	546.95
Investigations[3] (outpatient)	237	15,949.0	67.30	127.01	114	9,819.2	86.13	127.97	119	12,089.5	101.59	157.60
Prescriptions (outpatient)	237	6,667.5	28.13	16.04	114	3,344.4	29.34	17.08	119	3,393.7	28.52	15.38
**Inpatient - Bleeding episode (hospital+all other costs in bleeding event hospitalization)**	237	2,303,142.7	9717.90	2811.94	114	1,136,978.6	9973.50	4230.84	119	1,198,079.6	10067.90	4872.04
Hospitalizations (>24 hours)	237	2,041,080.7	8612.15	2563.05	114	1,009,775.2	8857.68	3811.77	119	1,062,883.4	8931.79	4497.17
Investigations[3] (within hospital)	237	38,433.5	162.17	113.07	114	20,607.9	180.77	117.46	119	20,359.7	171.09	111.45
Prescriptions (within hospital)	237	223,084.5	941.28	683.09	114	106,320.6	932.64	730.43	119	114,530.4	962.44	723.82
**Pharmacy & Investigation (global)**	237	284,134.5	1198.88	718.64	114	140,092.0	1228.88	754.73	119	150,373.3	1263.64	758.61
Prescriptions	237	229,752.0	969.42	683.45	114	109,665.0	961.97	729.54	119	117,924.1	990.96	724.85
Investigations[3] (global)	237	54,382.5	229.46	194.85	114	30,427.0	266.90	197.71	119	32,449.2	272.68	225.57
**Indirect cost**												
Cost of absence from work	237	163,881.2	691.48	2428.41	114	86,051.4	754.84	2571.99	119	70,978.7	596.46	2051.93
**Total Overall bleeding cost**	237	2,886,318.0	12178.56	3566.37	114	1,436,966.6	12604.97	4814.12	119	1,493,645.9	12551.65	4930.79

Type of FXai indication.

Time window[1]	Venous thromboembolism				Atrial fibrillation				Non-mechanical cardiac-valve replacement			
Cumulative costs	No. Patients	Total cost (€)	Mean costs per patient (€)	SD costs per patient (€)	No. Patients	Total cost (€)	Mean costs per patient (€)	SD costs per patient (€)	No. Patients	Total cost (€)	Mean costs per patient (€)	SD costs per patient (€)
**6 months since index date**												
**All-cause costs**												
**Outpatient**	83	93,818.7	1130.35	555.62	367	401,919.1	1095.15	499.30	20	18,678.0	933.90	619.04
GPs visit	83	37,317.8	449.61	259.30	367	163,506.0	445.52	251.44	20	6,540.2	327.01	268.76
Specialist visit	83	46,371.7	558.70	376.04	367	198,735.9	541.51	323.29	20	10,173.4	508.67	429.27
Investigations[3] (outpatient)	83	4,370.4	52.66	37.38	367	16,573.4	45.16	36.78	20	681.1	34.05	33.96
Prescriptions (outpatient)	83	5,681.8	68.46	36.99	367	22,811.8	62.16	39.99	20	1,271.3	63.57	42.63
**Inpatient (hospital+all other costs in hospitalization)**	83	862,516.9	10391.77	4254.76	367	3,868,451.8	10540.74	4369.62	20	205,686.0	10284.30	3975.81
Hospitalizations (>24 hours)	83	782,270.7	9424.95	4229.21	367	3,512,323.8	9570.36	4335.03	20	183,726.0	9186.30	4063.81
Investigations[3] (within hospital)	83	14,900.1	179.52	136.40	367	54,839.2	149.43	127.16	20	3,959.8	197.99	171.53
Prescriptions (within hospital)	83	65,177.1	785.27	539.11	367	300,602.7	819.08	589.10	20	17,965.3	898.26	552.71
**Pharmacy & Investigation (global)**	83	90,129.3	1085.90	592.08	367	394,827.2	1075.82	610.86	20	23,877.5	1193.87	558.73
Prescriptions (global)	83	70,858.8	853.72	546.33	367	323,414.6	881.24	590.76	20	19,236.6	961.83	564.40
Investigations[3] (global)	83	19,270.5	232.17	143.06	367	71,412.6	194.58	140.25	20	4,640.9	232.04	175.91
**Indirect cost**												
Cost of absence from work	83	78,378.0	944.31	3266.86	367	233,489.7	636.21	2397.34	20	17,265.1	863.25	2113.96
**Total Overall cost**	83	1,034,713.6	12466.43	5560.41	367	4,503,860.5	12272.10	4756.51	20	241,629.1	12081.46	3539.68
**Bleeding-related costs**												
**Outpatient**	83	82,278.4	991.31	520.43	367	351,521.1	957.82	462.33	20	16,317.0	815.85	596.96
GPs visits	83	33,598.9	404.81	229.87	367	146,065.4	398.00	219.63	20	5,899.0	294.95	243.85
Specialist visits	83	45,425.3	547.29	363.77	367	194,950.4	531.20	307.91	20	10,173.4	508.67	429.27
Investigations[3] (outpatient)	83	3,008.2	36.24	43.95	367	9,762.4	26.60	40.40	20	227.0	11.35	29.69
Prescriptions (outpatient)	83	193.0	2.33	4.57	367	570.9	1.56	3.77	20	13.5	0.68	3.02
**Inpatient - Bleeding episode (hospital+all other costs in bleeding event hospitalization)**	83	731,339.0	8811.31	1909.35	367	3,277,064.1	8929.33	2157.59	20	186,390.9	9319.55	2637.38
Hospitalizations (>24 hours)	83	656,676.8	7911.77	1858.60	367	2,941,768.6	8015.72	1996.89	20	165,784.0	8289.20	2639.24
Investigations[3] (within hospital)	83	9,423.7	113.54	92.40	367	34,590.3	94.25	72.77	20	2,627.3	131.37	105.49
Prescriptions (within hospital)	83	65,129.5	784.69	539.33	367	300,232.2	818.07	589.17	20	17,952.6	897.63	552.46
**Pharmacy & Investigation (global)**	83	77,754.4	936.80	553.07	367	345,155.8	940.48	595.35	20	20,820.5	1041.02	515.28
Prescriptions	83	65,322.5	787.02	540.14	367	300,803.1	819.63	589.45	20	17,966.1	898.30	551.49
Investigations[3] (global)	83	12,431.9	149.78	92.83	367	44,352.7	120.85	82.50	20	2,854.4	142.72	107.32
**Indirect cost**												
Cost of absence from work	83	64,127.4	772.62	2313.20	367	204,166.4	556.31	1994.52	20	17,265.1	863.25	2113.96
**Total Overall bleeding cost**	83	877,744.9	10575.24	2652.35	367	3,832,751.6	10443.46	2595.05	20	219,972.9	10998.65	2205.11
**1 year since index date**												
**All-cause costs**												
**Outpatient**	83	147,166.1	1773.09	758.77	367	635,597.9	1731.87	694.58	20	31,213.6	1560.68	909.33
GPs visit	83	55,848.5	672.87	360.97	367	250,773.3	683.31	332.08	20	10,451.6	522.58	361.09
Specialist visit	83	71,923.5	866.55	472.14	367	307,567.4	838.06	433.21	20	16,324.7	816.24	621.12
Investigations[3] (outpatient)	83	7,605.6	91.63	63.20	367	31,217.0	85.06	62.92	20	1,589.2	79.46	53.37
Prescriptions (outpatient)	83	11,654.5	140.42	67.67	367	45,490.2	123.95	63.25	20	2,820.1	141.01	62.82
**Inpatient**	83	924,316.1	11136.34	5005.09	367	4,265,382.7	11622.30	5649.18	20	206,750.2	10337.51	3975.82

Hospitalizations (>24 hours)	83	834,661.3	10056.16	4874.62	367	3,861,116.1	10520.75	5493.48	20	183,726.0	9186.30	4063.81
Investigations[3] (within hospital)	83	19,087.2	229.97	162.67	367	70,098.5	191.00	143.70	20	5,005.9	250.30	189.34
Prescriptions (within hospital)	83	70,332.5	847.38	662.97	367	333,195.1	907.89	718.68	20	17,965.3	898.26	552.71
**Pharmacy & Investigation (global)**	83	108,679.8	1309.40	743.66	367	480,000.7	1307.90	752.41	20	27,380.5	1369.03	567.12
Prescriptions (global)	83	81,987.0	987.80	673.12	367	378,685.3	1031.84	721.65	20	20,785.4	1039.27	564.25
Investigations[3] (global)	83	26,692.8	321.60	181.52	367	101,315.4	276.06	173.44	20	6,595.2	329.76	182.05
**Indirect cost**												
Cost of absence from work	83	78,378.0	944.31	3266.86	367	239,518.7	652.64	2503.54	20	17,265.1	863.25	2113.96
**Total Overall cost**	83	1,149,860.1	13853.74	6115.52	367	5,140,499.3	14006.81	5978.37	20	255,228.9	12761.44	3721.31
**Bleeding-related costs**												
**Outpatient**	83	120,900.1	1456.63	747.02	367	515,997.1	1405.99	701.09	20	25,770.7	1288.53	952.63
GPs visits	83	51,424.2	619.57	401.09	367	221,919.3	604.68	385.80	20	9,105.0	455.25	385.23
Specialist visits	83	61,986.7	746.83	525.77	367	268,056.8	730.40	455.93	20	15,615.0	780.75	716.38
Investigations[3] (outpatient)	83	5,221.7	62.91	79.29	367	16,289.6	44.39	73.43	20	454.1	22.70	59.39
Prescriptions (outpatient)	83	2,175.5	26.21	15.16	367	9,444.4	25.73	13.90	20	588.6	29.43	13.95
**Inpatient - Bleeding episode (hospital+all other costs in bleeding event hospitalization)**	83	739,260.3	8906.75	2123.92	367	3,446,582.5	9391.23	3161.69	20	185,768.8	9288.44	2642.23
Hospitalizations (>24 hours)	83	663,853.6	7998.24	2017.90	367	,092,481.3	8426.38	2900.08	20	165,784.0	8289.20	2639.24
Investigations[3] (within hospital)	83	9,305.9	112.12	79.64	367	38,319.9	104.41	77.88	20	2,005.2	100.26	94.72
Prescriptions (within hospital)	83	65,976.8	794.90	559.01	367	315,239.4	858.96	654.68	20	17,952.6	897.63	552.46
**Pharmacy & Investigation (global)**	83	82,679.9	996.14	557.70	367	379,293.2	1033.50	667.01	20	21,000.5	1050.02	543.46
Prescriptions	83	68,152.2	821.11	559.47	367	324,683.8	884.70	653.87	20	18,541.2	927.06	552.95
Investigations[3] (global)	83	14,527.6	175.03	108.61	367	54,609.5	148.80	114.23	20	2,459.3	122.96	122.38
**Indirect cost**												
Cost of absence from work	83	64,127.4	772.62	2313.20	367	212,113.8	577.97	2103.24	20	17,265.1	863.25	2113.96
**Total Overall bleeding cost**	83	924,287.8	11136.00	2847.06	367	4,174,693.5	11375.19	3470.22	20	228,804.5	11440.23	2271.24
**2 years since index date**												
**All-cause costs**												
**Outpatient**	83	201,765.1	2430.90	1031.24	367	859,053.3	2340.74	946.49	20	40,516.5	2025.82	1132.04
GPs visit	83	79,701.2	960.25	465.94	367	345,606.8	941.71	427.29	20	15,709.4	785.47	546.41
Specialist visit	83	98,185.0	1182.95	628.14	367	405,515.8	1104.95	587.01	20	18,454.0	922.70	719.66
Investigations[3] (outpatient)	83	8,457.0	101.89	59.30	367	35,700.8	97.28	58.06	20	1,816.3	90.81	46.59
Prescriptions (outpatient)	83	15,273.0	184.01	93.08	367	71,600.8	195.10	90.30	20	4,504.8	225.24	115.82
**Inpatient**	83	979,944.4	11806.56	5650.22	367	4,554,464.2	12409.98	6290.65	20	212,522.1	10626.10	3778.79
Hospitalizations (>24 hours)	83	883,463.6	10644.14	5481.54	367	4,102,974.1	11179.77	5961.91	20	188,032.0	9401.60	3902.52
Investigations[3] (within hospital)	83	22,857.5	275.39	193.43	367	84,689.1	230.76	167.17	20	5,892.4	294.62	207.69
Prescriptions (within hospital)	83	73,331.4	883.51	757.25	367	365,580.9	996.13	899.14	20	18,532.6	926.63	549.38
**Pharmacy & Investigation (global)**	83	119,918.7	1444.80	845.94	367	557,571.7	1519.27	946.30	20	30,746.1	1537.30	544.61
Prescriptions (global)	83	88,604.3	1067.52	764.74	367	437,181.7	1191.23	899.72	20	23,037.4	1151.87	565.19
Investigations[3] (global)	83	31,314.4	377.28	218.02	367	120,390.0	328.04	195.64	20	7,708.7	385.43	204.77
**Indirect cost**												
Cost of absence from work	83	84,681.1	1020.25	3454.38	367	271,582.4	740.01	2860.76	20	22,472.0	1123.60	2864.47
**Total Overall cost**	83	1,266,390.5	15257.72	6750.85	367	5,685,099.9	15490.74	6864.71	20	275,510.6	13775.53	4271.26
**Bleeding-related costs**												
**Outpatient**	83	147,691.1	1779.41	830.00	367	614,953.6	1675.62	769.68	20	27,217.7	1360.89	878.54
GPs visits	83	58,798.0	708.41	346.15	367	252,312.2	687.50	320.49	20	10,836.3	541.81	356.99
Specialist visits	83	79,257.8	954.91	505.87	367	329,806.9	898.66	497.92	20	15,141.8	757.09	567.19
Investigations[3] (outpatient)	83	7,151.5	86.16	111.55	367	22,419.4	61.09	104.47	20	681.1	34.05	89.08
Prescriptions (outpatient)	83	2,357.8	28.41	15.27	367	10,020.0	27.30	16.13	20	546.6	27.33	11.48

**Inpatient - Bleeding episode (hospital+all other costs in bleeding event hospitalization)**	83	771,686.5	9297.43	2618.79	367	3,551,673.8	9677.59	3638.45	20	187,016.5	9350.82	2676.50
Hospitalizations (>24 hours)	83	687,537.0	8283.58	2507.00	367	3,164,249.2	8621.93	3330.69	20	165,784.0	8289.20	2639.24
Investigations[3] (within hospital)	83	14,897.8	179.49	127.84	367	54,778.9	149.26	99.96	20	2,651.3	132.56	99.23
Prescriptions (within hospital)	83	69,057.6	832.02	569.24	367	331,837.6	904.19	690.15	20	18,541.2	927.06	552.95
**Pharmacy & Investigation (global)**	83	93,464.8	1126.08	628.60	367	419,056.0	1141.84	713.14	20	22,420.2	1121.01	572.01
Prescriptions	83	71,415.4	860.43	569.54	367	341,857.7	931.49	690.19	20	19,087.8	954.39	552.32
Investigations[3] (global)	83	22,049.4	265.66	199.73	367	77,198.4	210.35	171.56	20	3,332.4	166.62	137.02
**Indirect cost**												
Cost of absence from work	83	71,526.8	861.77	2638.02	367	219,513.2	598.13	2191.87	20	17,265.1	863.25	2113.96
**Total Overall bleeding cost**	83	990,904.3	11938.61	3607.70	367	4,386,140.5	11951.34	3982.08	20	231,499.3	11574.96	2237.80
**3 years since index date**												
**All-cause costs**												
**Outpatient**	83	256,028.5	3084.68	1205.81	367	1,108,277.7	3019.83	1211.64	20	49,174.8	2458.74	1445.05
GPs visit	83	103,489.7	1246.86	580.91	367	464,100.6	1264.58	637.28	20	19,749.0	987.45	690.72
Specialist visit	83	121,370.8	1462.30	671.36	367	506,776.5	1380.86	662.77	20	22,239.5	1111.97	841.21
Investigations[3] (outpatient)	83	9,478.6	114.20	60.44	367	41,887.5	114.13	57.37	20	2,327.1	116.35	46.86
Prescriptions (outpatient)	83	21,522.4	259.31	122.38	367	94,775.2	258.24	112.97	20	4,818.3	240.91	91.23
**Inpatient**	83	1,005,468.4	12114.08	5947.13	367	4,818,369.8	13129.07	7167.78	20	214,669.4	10733.47	3746.03
Hospitalizations (>24 hours)	83	899,252.5	10834.37	5671.67	367	4,248,663.1	11576.74	6198.02	20	188,032.0	9401.60	3902.52
Investigations[3] (within hospital)	83	26,769.0	322.52	221.02	367	103,995.2	283.37	190.26	20	6,891.1	344.56	270.14
Prescriptions (within hospital)	83	79,097.9	952.99	972.77	367	464,219.5	1264.90	2085.97	20	19,667.2	983.36	625.32
**Pharmacy & Investigation (global)**	83	136,867.9	1649.01	1053.94	367	704,877.4	1920.65	2138.84	20	33,703.7	1685.18	594.45
Prescriptions (global)	83	100,620.3	1212.29	973.00	367	558,994.7	1523.15	2093.12	20	24,485.5	1224.27	636.25
Investigations[3] (global)	83	36,247.6	436.72	261.88	367	145,882.7	397.50	219.09	20	9,218.2	460.91	291.54
**Indirect cost**												
Cost of absence from work	83	84,681.1	1020.25	3454.38	367	283,914.6	773.61	3019.62	20	22,472.0	1123.60	2864.47
**Total Overall cost**	83	1,346,178.0	16219.01	7084.37	367	6,210,562.2	16922.51	7909.49	20	286,316.2	14315.81	4383.74
**Bleeding-related costs**												
**Outpatient**	83	161,443.4	1945.10	930.72	367	666,612.3	1816.38	861.18	20	29,762.6	1488.13	960.53
GPs visits	83	65,081.8	784.12	391.35	367	277,896.1	757.21	360.99	20	11,926.3	596.32	385.90
Specialist visits	83	84,935.9	1023.32	555.60	367	350,390.3	954.74	544.49	20	16,324.7	816.24	625.84
Investigations[3] (outpatient)	83	8,967.8	108.05	144.74	367	27,981.7	76.24	134.35	20	908.1	45.41	118.77
Prescriptions (outpatient)	83	2,457.9	29.61	18.42	367	10,344.2	28.19	15.73	20	603.4	30.17	12.86
**Inpatient - Bleeding episode (hospital+all other costs in bleeding event hospitalization)**	83	773,585.4	9320.31	2602.34	367	3,676,548.3	10017.84	4039.74	20	188,067.3	9403.36	2668.88
Hospitalizations (>24 hours)	83	687,537.0	8283.58	2507.00	367	3,260,418.3	8883.97	3663.37	20	165,784.0	8289.20	2639.24
Investigations[3] (within hospital)	83	14,426.0	173.81	113.60	367	61,823.6	168.46	114.47	20	3,151.5	157.58	105.33
Prescriptions (within hospital)	83	71,415.4	860.43	569.54	367	353,432.3	963.03	737.70	20	19,087.8	954.39	552.32
**Pharmacy & Investigation (global)**	83	97,267.1	1171.89	620.98	367	453,581.9	1235.92	767.63	20	23,750.8	1187.54	602.62
Prescriptions	83	73,873.3	890.04	571.36	367	363,776.5	991.22	737.58	20	19,691.2	984.56	554.98
Investigations[3] (global)	83	23,393.8	281.85	225.19	367	89,805.4	244.70	200.02	20	4,059.6	202.98	182.78
**Indirect cost**												
Cost of absence from work	83	71,526.8	861.77	2638.02	367	232,119.4	632.48	2324.60	20	17,265.1	863.25	2113.96
**Total Overall bleeding cost**	83	1,006,555.6	12127.18	3632.71	367	4,575,280.0	12466.70	4470.39	20	235,094.9	11754.75	2248.28

1. Index date: day of the first major bleeding; 2. Laboratory/radiology investigations; Quantitative variables are presented as mean and (Standard deviation): qualitative variables are presented by their absolute frequencies. FXai; Factor Xa inhibitors; GIB: gastrointestinal bleeding; GP: general practitioners; ICH: intracranial bleeding; MB: major bleeding.

Supplementary Table S8. Independent variables associated with outcomes (Fine and Gray regression models in the case of myocardial infarction and stroke and Cox regression models in the case of overall or all-cause mortality).

Acute myocardial infarction

	exp(coef)	2,50%	97,50%	p-value
Medical possesion ratio to FXai (% prescribed doses taken)	0,99	0,91	1,08	0,896
% patients with treatment compliance =/>80%	0,77	0,20	2,90	0,695
% of patients discontinueing after 1st major bleeding	1,60	0,71	3,63	0,258
Switch from 1st FXAI to other FXAI	1,08	0,55	2,09	0,825
**FXai indications**				
v36Atrial fibrillation as reference	-	-	-	-
v36Non-mechanical cardiac-valve replacement	0,82	0,16	4,13	0,808
v36Venous thromboembolism	1,32	0,59	2,93	0,499
**FXai dose as per label (only for "dose as per label" and FXai columns)**				
v38Dose reduction as per label	0,48	0,18	1,29	0,145
v38No dose reduction	1,28	0,56	2,91	0,558
Age at index date (years)	1,01	0,97	1,04	0,750
Gender (Female as reference)	-	-	-	-
Gender (male)	0,86	0,46	1,62	0,650
BMI	0,96	0,90	1,02	0,203
Smoking (no use as reference)	-	-	-	-
Smoking	4,52	0,43	47,72	0,210
Type 1 diabetes	-	-	-	-
Type 2 diabetes	10,13	1,63	63,10	0,013
Chronic kidney disease	2,18	0,67	7,14	0,197
Hypercholesterolemia	0,79	0,36	1,74	0,558
Hypertension	3,05	1,13	8,22	0,027
Coronary artery disease	2,03	0,55	7,41	0,285
Stroke (Ausence as reference)	-	-	-	-
Stroke	3,13	0,70	13,92	0,134
Transitory ischemic attack	1,29	0,21	7,83	0,781
Heart failure	0,58	0,11	3,02	0,513
Updated Charlson comorbidity index	1,01	0,60	1,72	0,957
**Components**				
Myocardial infarction	0,69	0,18	2,57	0,576
Congestive heart failure	2,28	0,39	13,25	0,359
Peripheral vascular disease	1,58	0,51	4,90	0,426
Cerebrovascular disease	0,10	0,01	1,10	0,060
Hemiplegia or paraplegia	2,15	0,43	10,85	0,355
Chronic pulmonary disease	0,90	0,36	2,26	0,825
Rheumatologic disease	4,68	1,32	16,56	0,017
Peptic ulcer disease	0,26	0,02	2,79	0,264
Diabetes without chronic complications	0,15	0,02	0,99	0,049
Diabetes with chronic complications	0,05	0,00	0,55	0,014
Renal disease	0,35	0,10	1,24	0,105
Any malignancy (inc leukaemia and lymphoma)	1,42	0,24	8,54	0,700
Metastatic solid tumour	0,56	0,01	30,21	0,776
**Liver disease**				
Mild liver disease	1,33	0,30	5,90	0,709
Moderate or severe liver disease	2,15	0,14	33,49	0,583
AIDS/HIV	0,30	0,01	11,63	0,519
Major bleeding history >60 days prior to FXa start	0,96	0,34	2,70	0,943
Gastrointestinal diseases	3,44	1,39	8,51	0,007
Anemia	0,48	0,19	1,17	0,107
Urological diseases	0,79	0,23	2,71	0,702
**Lab test**				
Hemoglobin	0,75	0,61	0,93	0,010
HbA1c	1,32	1,03	1,70	0,030
Platelet count	1,00	1,00	1,00	0,996
eGFR	1,01	0,98	1,04	0,484
Creatinine clearance	1,01	1,00	1,03	0,058
**Comedications**				
History of non-FXai AC use	1,01	0,54	1,91	0,967

Lipid lowering therapies	1,06	0,45	2,48	0,902
Nitrates	2,00	0,56	7,15	0,286
Anticancer therapies	0,90	0,16	5,02	0,905
NSAIDs	0,83	0,42	1,62	0,583
Antiplatelet drugs	0,63	0,25	1,58	0,328
Antidepressants	1,66	0,71	3,86	0,240
Antivirals	8,10	1,39	47,18	0,020
Corticosteroids	0,42	0,10	1,76	0,235
Gastroprotective agents	0,80	0,38	1,69	0,562
Hormone therapies	1,77	0,30	10,41	0,526
Macrolides	2,09	0,55	8,00	0,282

Stroke

	exp(coef)	2,50%	97,50%	p-value
Calendar year of first FXai use 2013 as reference	-	-	-	-
2014	0,46	0,03	8,14	0,595
2015	0,27	0,02	4,39	0,355
2016	0,06	0,00	1,76	0,101
2017	0,38	0,03	5,23	0,468
2018	0,29	0,02	3,95	0,355
2019	0,36	0,03	4,74	0,437
2020	0,12	0,01	2,12	0,149
2021	0,60	0,05	7,74	0,699
2022	0,89	0,06	12,48	0,932
Medical possesion ratio to FXai (% prescribed doses taken)	1,00	0,91	1,09	0,984
% patients with treatment compliance =/>80%	1,31	0,26	6,47	0,741
% of patients discontinueing after 1st major bleeding	1,17	0,48	2,85	0,729
Switch from 1st FXai to other FXai	0,71	0,33	1,56	0,399
**FXai dose as per label (only for "dose as per label" and FXai columns)**				
v38Dose reduction as per label	0,22	0,07	0,75	0,015
v38No dose reduction	0,47	0,19	1,14	0,094
Age at index date (years)	1,03	0,93	1,14	0,591
Age groups (45-64) as reference	-	-	-	-
Age groups (65-74)	1,42	0,24	8,31	0,698
Age groups (75-84)	0,66	0,05	8,24	0,744
Age groups (≥85)	0,61	0,02	20,62	0,782
Gender (Female as reference)	-	-	-	-
Gender (male)	0,56	0,27	1,18	0,126
BMI	0,93	0,87	0,99	0,021
Smoking (no use as reference)	-	-	-	-
Smoking	5,53	0,46	66,14	0,177
Type 1 diabetes	3,40	0,25	45,52	0,355
Type 2 diabetes	2,84	0,41	19,57	0,290
Chronic kidney disease	0,54	0,16	1,89	0,335
Hypercholesterolemia	1,46	0,57	3,78	0,430
Hypertension	0,61	0,27	1,38	0,235
Coronary artery disease	2,19	0,50	9,63	0,299
Stroke (Ausence as reference)	-	-	-	-
Stroke	0,20	0,02	1,68	0,139
Transitory ischemic attack	0,44	0,06	3,30	0,421
Heart failure	0,95	0,18	5,10	0,953
Updated Charlson comorbidity index	0,98	0,65	1,48	0,932
**Components**				
Myocardial infarction	0,19	0,03	1,32	0,093
Congestive heart failure	1,53	0,25	9,51	0,646
Peripheral vascular disease	1,19	0,30	4,79	0,805
Cerebrovascular disease	4,06	0,54	30,82	0,175
Hemiplegia or paraplegia	1,00	0,17	5,75	1,000
Dementia	1,30	0,58	2,95	0,524
Chronic pulmonary disease	0,77	0,28	2,10	0,613
Rheumatologic disease	3,68	0,78	17,44	0,100
Peptic ulcer disease	1,62	0,39	6,84	0,510
Diabetes without chronic complications	0,58	0,08	4,33	0,592
Diabetes with chronic complications	0,24	0,02	2,49	0,233
Renal disease	2,59	0,92	7,33	0,073
Any malignancy (inc leukaemia and lymphoma)	3,08	0,55	17,33	0,202
Metastatic solid tumour	2,88	0,05	164,36	0,608
**Liver disease**				
Mild liver disease	3,35	0,91	12,31	0,069
Moderate or severe liver disease	5,94	0,59	59,53	0,130
Major bleeding history >60 days prior to FXa start	4,15	1,44	11,95	0,008
Gastrointestinal diseases	1,60	0,51	5,01	0,419
Anemia	0,70	0,27	1,84	0,474
Urological diseases	1,40	0,36	5,42	0,629
**Lab test**				
Hemoglobin	0,84	0,66	1,07	0,160
HbA1c	1,49	1,11	2,01	0,008

Platelet count	1,00	1,00	1,00	0,409
eGFR	1,01	0,98	1,04	0,639
Creatinine clearance	1,01	0,99	1,02	0,577
**Comedications**				
History of non-FXai AC use	0,76	0,36	1,62	0,483
Lipid lowering therapies	0,48	0,17	1,36	0,168
Nitrates	1,52	0,31	7,49	0,607
Anticancer therapies	0,33	0,04	2,75	0,308
NSAIDs	1,02	0,49	2,15	0,948
Antiplatelet drugs	1,28	0,50	3,28	0,610
Antidepressants	0,54	0,17	1,78	0,312
Corticosteroids	1,34	0,39	4,67	0,641
Gastroprotective agents	1,00	0,43	2,29	0,996
Hormone therapies	21,59	4,23	110,27	0,000
Macrolides	0,22	0,02	2,14	0,190

Cardiovascular death

	exp(coef)	2,50%	97,50%	p-value
Medical possesion ratio to FXai (% prescribed doses taken)	0,98	0,92	1,05	0,610
% patients with treatment compliance =/>80%	1,76	0,54	5,77	0,349
% of patients discontinueing after 1st major bleeding	0,67	0,37	1,21	0,187
Switch from 1st FXAI to other FXai	1,06	0,62	1,80	0,834
**FXai indications**				
v36Atrial fibrillation as reference	-	-	-	-
v36Non-mechanical cardiac-valve replacement	2,42	0,97	6,05	0,059
v36Venous thromboembolism	1,06	0,54	2,07	0,876
**FXai dose as per label (only for "dose as per label" and FXai columns)**				
v38Dose reduction as per label	0,33	0,15	0,72	0,005
v38No dose reduction	0,49	0,26	0,94	0,033
Age at index date (years)	0,98	0,91	1,05	0,530
Age groups (45-64) as reference	-	-	-	-
Age groups (65-74)	0,44	0,04	5,25	0,520
Age groups (75-84)	0,90	0,17	4,61	0,896
Age groups (≥85)	0,86	0,30	2,46	0,785
Gender (Female as reference)	-	-	-	-
Gender (male)	0,93	0,55	1,57	0,785
BMI	1,04	0,99	1,09	0,142
Smoking (no use as reference)	-	-	-	-
Smoking	3,92	0,46	33,67	0,213
Chronic kidney disease	0,54	0,19	1,55	0,253
Hypercholesterolemia	1,26	0,64	2,49	0,501
Hypertension	1,55	0,81	2,97	0,188
Coronary artery disease	1,38	0,51	3,79	0,527
Stroke (Ausence as reference)	-	-	-	-
Stroke	0,85	0,27	2,69	0,781
Transitory ischemic attack	0,42	0,10	1,72	0,227
Heart failure	0,58	0,14	2,33	0,441
Updated Charlson comorbidity index	0,88	0,59	1,32	0,533
**Components**				
Myocardial infarction	0,50	0,15	1,68	0,261
Congestive heart failure	1,52	0,35	6,65	0,578
Peripheral vascular disease	3,46	1,55	7,71	0,002
Cerebrovascular disease	1,67	0,43	6,41	0,456
Hemiplegia or paraplegia	0,99	0,22	4,53	0,990
Chronic pulmonary disease	0,95	0,46	1,98	0,899
Rheumatologic disease	1,31	0,43	3,97	0,632
Peptic ulcer disease	0,37	0,08	1,72	0,205
Diabetes without chronic complications	0,71	0,16	3,28	0,665
Diabetes with chronic complications	0,95	0,17	5,18	0,948
Renal disease	1,06	0,41	2,72	0,908
Any malignancy (inc leukaemia and lymphoma)	0,54	0,06	5,13	0,594
Metastatic solid tumour	2,86	0,13	65,45	0,510
**Liver disease**				
Mild liver disease	0,53	0,07	3,81	0,527
Moderate or severe liver disease	6,80	0,43	107,17	0,173
Major bleeding history >60 days prior to FXa start	1,44	0,66	3,13	0,363
Gastrointestinal diseases	0,68	0,27	1,72	0,415
Anemia	0,82	0,40	1,67	0,579
Urological diseases	0,75	0,22	2,57	0,642
**Lab test**				
Hemoglobin	1,01	0,86	1,19	0,908
HbA1c	1,13	0,92	1,40	0,244
Platelet count	1,00	1,00	1,00	0,489
eGFR	0,99	0,98	1,01	0,368
Creatinine clearance	1,00	0,99	1,01	0,946
**Comedications**				
History of non-FXai AC use	0,49	0,29	0,84	0,009
Lipid lowering therapies	0,82	0,39	1,76	0,615
Antidiabetic drugs	1,64	0,38	7,02	0,503
Nitrates	0,99	0,33	2,99	0,985
Anticancer therapies	3,60	0,37	34,98	0,269
NSAIDs	1,53	0,90	2,61	0,115

Antiplatelet drugs	0,73	0,33	1,59	0,425
Antidepressants	0,41	0,17	0,99	0,047
Corticosteroids	2,05	0,92	4,58	0,079
Gastroprotective agents	1,31	0,72	2,39	0,379
Hormone therapies	2,95	0,88	9,81	0,078
Macrolides	0,64	0,13	3,06	0,578

Overall death

	exp(coef)	lower .95	upper .95	Pr(>|z|)
Medical possesion ratio to FXai (% prescribed doses taken)	0,98	0,93	1,02	0,293
% patients with treatment compliance =/>80%	1,86	0,79	4,36	0,156
% of patients discontinueing after 1st major bleeding	0,87	0,55	1,36	0,534
Switch from 1st FXai to other FXai	1,24	0,85	1,81	0,270
**FXai indications**				
v36Atrial fibrillation as reference				
v36Non-mechanical cardiac-valve replacement	1,50	0,69	3,25	0,302
v36Venous thromboembolism	0,87	0,53	1,42	0,565
**FXai dose as per label (only for "dose as per label" and FXai columns)**				
v38Dose reduction as per label	0,55	0,31	0,97	0,038
v38No dose reduction	0,65	0,39	1,09	0,100
Age at index date (years)	0,98	0,93	1,04	0,521
Age groups (45-64) as reference				
Age groups (65-74)	1,35	0,54	3,40	0,525
Age groups (75-84)	1,70	0,47	6,13	0,419
Age groups (≥85)	1,99	0,33	12,01	0,454
Gender (Female as reference)				
Gender (male)	0,95	0,65	1,39	0,789
BMI	1,01	0,98	1,05	0,577
Smoking (no use as reference)				
Smoking	2,55	0,55	11,92	0,234
Chronic kidney disease	0,28	0,13	0,59	0,001
Hypercholesterolemia	1,17	0,71	1,91	0,542
Hypertension	1,55	0,96	2,49	0,070
Coronary artery disease	2,42	1,21	4,81	0,012
Stroke (Ausence as reference)				
Stroke	1,11	0,48	2,54	0,809
Transitory ischemic attack	0,31	0,09	0,99	0,048
Heart failure	1,01	0,40	2,50	0,990
Updated Charlson comorbidity index	1,07	0,81	1,41	0,645
**Components**				
Myocardial infarction	0,36	0,15	0,87	0,023
Congestive heart failure	0,84	0,31	2,28	0,736
Peripheral vascular disease	1,03	0,55	1,94	0,917
Cerebrovascular disease	1,53	0,57	4,13	0,403
Hemiplegia or paraplegia	0,83	0,30	2,34	0,728
Chronic pulmonary disease	0,90	0,53	1,54	0,699
Rheumatologic disease	2,81	1,34	5,88	0,006
Peptic ulcer disease	0,39	0,15	1,04	0,061
Diabetes without chronic complications	1,12	0,36	3,45	0,842
Diabetes with chronic complications	0,91	0,26	3,20	0,884
Renal disease	1,54	0,82	2,90	0,182
Any malignancy (inc leukaemia and lymphoma)	0,52	0,15	1,79	0,296
Metastatic solid tumour	0,58	0,05	6,42	0,660
**Liver disease**				
Mild liver disease	0,57	0,17	1,91	0,361
Moderate or severe liver disease	0,63	0,09	4,30	0,635
Major bleeding history >60 days prior to FXa start	1,75	1,04	2,94	0,035
Gastrointestinal diseases	0,87	0,46	1,64	0,659
Anemia	0,76	0,46	1,26	0,292
Urological diseases	0,61	0,25	1,47	0,268
**Lab test**				
Hemoglobin	0,97	0,86	1,10	0,623
HbA1c	1,26	1,08	1,47	0,003
Platelet count	1,00	1,00	1,00	0,788
eGFR	1,00	0,98	1,01	0,494
Creatinine clearance	1,01	1,00	1,01	0,169
**Comedications**				
History of non-FXai AC use	0,86	0,60	1,23	0,395
Lipid lowering therapies	0,90	0,53	1,54	0,713
Antidiabetic drugs	0,86	0,30	2,43	0,772
Nitrates	0,85	0,39	1,84	0,678
Anticancer therapies	1,32	0,35	4,92	0,683
NSAIDs	0,83	0,55	1,23	0,346

Antiplatelet drugs	0,86	0,52	1,42	0,553
Antidepressants	0,90	0,53	1,54	0,707
Corticosteroids	1,41	0,77	2,58	0,261
Gastroprotective agents	1,06	0,68	1,65	0,808
Hormone therapies	2,64	1,03	6,77	0,043
Macrolides	0,52	0,17	1,56	0,242

AIDS: acquired immunodeficiency syndrome; BMI: body mass index; eGFR: estimated glomerular filtration rate; FXai; Factor Xa inhibitors; HIV: human immunodeficiency virus; NSAIDs: non-steroidal anti-inflammatory drugs.
